# Supplementary material for: Classical cadherins evolutionary constraints in primates is associated with their expression in the central nervous system
Source: PLoS One. 2024 Nov 21;19(11):e0313428. doi: 10.1371/journal.pone.0313428 (PMC11581309; doi:10.1371/journal.pone.0313428)
Supplement: S1 Table — (PDF) [file pone.0313428.s001.pdf]

S1 Table. Species used in this study and their time of divergence in Millon Years Ago (MYr) based on A. Purvis Phyl Trans Royal Soc 348:405 (1995)						
**Species**	**NCBI Taxid**	**Common name**	**Time of divergence MYr**	**Family**	**Suborder**	**Semiorder**
**Primates**						
Homo sapiens	9606	Human	0	Hominidae (Great Apes)	Anthhropidea	Haplorhini
Pan troglodytes	9598	Chimpamzee	7.04			
Pan paniscus	9597	Bonobo	7.04			
Gorilla gorilla	9595	Gorilla	8.09			
Pongo abelii	9601	Orangutan	14.5			
Nomascus leucogenys	61853	Gibbon - Northern white-cheeked gibbon	17.58	Hylobatidae (Laser Apes)		
Macaca mulatta	9544	Rhesus monkey	27.5	Cercopithecidae (Old World Monkeys)		
Macaca fascicularis	9541	Crab-eating macaque / long-tailed macaque	27.5			
Papio anubis	9555	Baboon anubis	27.5			
Chlorocebus sabaeus	60711	Green monkey	27.5			
Rhinopithecus roxellana	61622	Golden snub-nose monkey	27.5			
Saimiri boliviensis	39432	Bolivian squirrel monkey	39.88	Cabidae (New World Monkeys)		
Callithrix jacchus	9483	Marmoset - white-tufted-ear marmoset	39.88			
Tarsius syrichta (Carlito syrichta)	1868482	Tarsier - Philippine tarsier	49.61	Tarsidea	Tarsiiformes	
Otolemur garnettii	30611	Bushbaby - small-eared galago	57.17	Galagidae	Strepsirhini	Strepsirhini
Microcebus murinus	30608	Mouse lemur - gray mouse lemur	57.17	Cheirogalaidae		
**Non-primates**						
Mus musculus	10090	House mouse	81	Muridae		
