# Supplementary material for: Classical cadherins evolutionary constraints in primates is associated with their expression in the central nervous system
Source: PLoS One. 2024 Nov 21;19(11):e0313428. doi: 10.1371/journal.pone.0313428 (PMC11581309; doi:10.1371/journal.pone.0313428)
Supplement: S2 Table — (PDF) [file pone.0313428.s002.pdf]

S2 Table. Protein and transcript sequence accession numbers used in this study

Protein accession numbers

Species	Common name	Taxid	MYr *	Type I Classical Cadherins			
				**CDH1**	**CDH2**	**CDH3**	**CDH4**
Homo sapiens	Human	9606	0	P12830	P19022	P22223	P55283
Pan troglodytes	Chimp	9598	7.04	A0A2J8Q404	A0A2I3TAH6	H2QBE4	H2QKP9
Pan paniscus	Bonobo	9597	7.04	XP_034796439	A0A2R9BHQ4	A0A2R9BYH8	A0A2R9AIR8
Gorilla gorilla	Gorilla	9595	8.09	G3QT90	G3SCJ6	G3QD40	G3QD69
Pongo pygmaeus abelii	Orangutan	9601	14.5	Q5RAX1	Q5R9X1	A0A2J8VVD5	H2P2H3
Nomascus leucogenys	Gibbon	61853	17.58	G1QZZ0	A0A2I3HIW8	G1QZQ9	XP_030681640
Macaca mulatta	Rhesus macaque	9544	27.5	F7H4U0	F6WI86	F6RPA3	F7DGM2
Macaca fascicularis	Crab-eating macaque	9541	27.5	A0A2K5V299	XP_005587037	A0A2K5WPN9	XP_005569561
Papio anubis	Baboon	9555	27.5	A0A096MNS2	A0A096N721	A0A096NFR1	A0A096NNE2
Chlorocebus sabaeus	Green monkey	60711	27.5	A0A0D9QVR1	A0A0D9RZ03	A0A0D9QVQ9	A0A0D9RT41
Rhinopithecus roxellana	Golden snub-nosed monk	61622	27.5	A0A2K6R725	A0A2K6R4K5	A0A2K6RCS8	A0A2K6P2W3
Saimiri boliviensis	Squirrel monkey	39432	39.88	XP_010345147.1	A0A2K6UQB3	A0A2K6T684	A0A2K6TU79
Callithrix jacchus	Marmoset	9483	39.88	F6WRS2	B0KW95	F6ZMA4	A0A2R8M804
Tarsier - Tarsius syrichta	Tarsier	1868482	49.61	Assembled from: KEKE944053v1-KE947423v1-XM_008070049	A0A3Q0EH64	A0A1U7SWI8	A0A1U7UB76
Otolemur garnettii	Bushbaby	30611	57.17	N/A	B4USZ0	HOXOU7	H0WXC1
Microcebus murinus	Mouse lemur	30608	57.17	A0A8B7ERW7	A0A8C5XAW1	A0A8C5XD28	A0A8B7WPU0
Mus musculus	Mouse	10090	81	A0A0R4IZW5	P15116	P10287	P39038

Species	Common name	Taxid	MYr *	Type II Classical Cadherins - Group A			Type II Classical Cadherins - Group B				
				**CDH6**	**CDH9**	**CDH10**	**CDH7**	**CDH12**	**CDH18**	**CDH20**	**CDH22**
Homo sapiens	Human	9606	0	P55285	Q9ULB4	Q9Y6N8	Q9ULB5	P55289	Q13634	Q9HBT6	Q9UJ99
Pan troglodytes	Chimp	9598	7.04	A0A803KIT6	H2QQP8	A0A6D2WEH8	A0A2J8ND39	A0A6D2WVR8	H2QQP5	A0A2J8NDG6	A0A2J8MJR4
Pan paniscus	Bonobo	9597	7.04	A0A2R9AUM4	A0A2R9AV11	A0A2R9B9J3	A0A2R9C879	Assembled from XM_034960250.1	U3CRG8	A0A2R9ARR9	XP_034803106.1
Gorilla gorilla	Gorilla	9595	8.09	G3RLA5	G3R9J0	G3R9R6	G3R6F1	G3RKX5	A0A2R9C061	G3QVJ7	XP_030860785.1
Pongo pygmaeus abelii	Orangutan	9601	14.5	H2PF91	A0A2J8WAU9	A0A663DD08	A0A2J8W3Q1	A0A2J8USV4	G3R5X3	A0A2J8W3C3	A0A2J8XVD2
Nomascus leucogenys	Gibbon	61853	17.58	G1RUL4	G1RN01	G1RMW8	G1RCP4	XP_030669794.1	H2PF84	G1RAV5	XP_030681465.1
Macaca mulatta	Rhesus macaque	9544	27.5	G7MUQ7	F7GUW1	F7DRD8	F6ZW11	F7HPS8	G1RMU3	G7NKT4	F7GIE7
Macaca fascicularis	Crab-eating macaque	9541	27.5	A0A2K5TW62	G7P7A8	G7P7A7	G7PW74	G7P7A5	H9ERS5	A0A7N9CZ75	XP_045218981.1
Papio anubis	Baboon	9555	27.5	A0A096MV12	A0A096MML6	A0A096MLZ6	A0A096NQS1	A0A096MSE6	A0A2K5UPM6	A0A096MT19	XP_021776679.2
Chlorocebus sabaeus	Green monkey	60711	27.5	A0A0D9RXA5	XP_007959457	A0A0D9RXC1	A0A0D9S039	XP_007959452.1	A0A096MM45	A0A0D9S004	XP_008013599.2
Rhinopithecus roxellana	Golden snub-nosed monk	61622	27.5	A0A2K6P4K0	A0A2K6Q124	A0A2K6P747	A0A2K6RV23	XP_030783714.1	A0A0D9RXE3	A0A2K6PGJ4	XP_010382485.2
Saimiri boliviensis	Squirrel monkey	39432	39.88	A0A2K6U5B8	A0A2K6TH86	A0A2K6TYX9	A0A2K6T198	A0A2K6TJC5	XP_039328127.1	A0A2K6RYM0	XP_039335633.1
Callithrix jacchus	Marmoset	9483	39.88	F7IMY6	F7ITB7	U3ETI0	F7ILV6	U3EPZ9	A0A2K6SGK6	F7GWC6	XP_035154635.1
Tarsier - Tarsius syrichta	Tarsier	1868482	49.61	A0A1U7TGI4	A0A1U7T0R0	A0A1U7TA68	A0A1U7TXY1	Assembled from KE939644v1-KE950865v1	Assembled from KE948787v1	A0A1U7TBK8	N/A
Otolemur garnettii	Bushbaby	30611	57.17	H0WS14	0A8B7GDA7	H0WYM1	H0WWQ7	XP_003789584.1	H0WQ36	H0XAG4	H0X0Z4
Microcebus murinus	Mouse lemur	30608	57.17	A0A8B7XCU5	H0XBT2	0A8B7GDV1	A0A8C5VYT2	A0A8B7XCT6	A0A8C6ECY8	A0A8B7H1L0	A0A8B7EV98
Mus musculus	Mouse	10090	81	P97326	P70407	P70408	B2RUF4	Q5RJH3	E9Q9Q6	Q9Z0M3	Q9WTP5

Species	Common name	Taxid	MYr *	Type II Classical Cadherins - Group C			Type II Classical Cadherins - Ungrouped		
				**CDH8**	**CDH11**	**CDH24**	**CDH5**	**CDH19**	**CDH13**
Homo sapiens	Human	9606	0	P55286	P55287	A8K0L1	P33151	Q9H159	P55290
Pan troglodytes	Chimp	9598	7.04	A0A2J8MVG2	A0A6D2Y107	A0A6D2X7D6	A0A6D2WX74	A0A6D2VVJ1	K7DFV8
Pan paniscus	Bonobo	9597	7.04	XP_034796232.1	A0A2R8ZND1	A0A096NWI7	A0A2R9B8B3	A0A2R9BH09	A0A2R9BUA4
Gorilla gorilla	Gorilla	9595	8.09	G3S1A0	G3QKI4	A0A2I2Z4R1	G3SFI0	G3S948	G3R9F2
Pongo pygmaeus abelii	Orangutan	9601	14.5	A0A2J8RYN6	A0A663DIN4	A0A2J8TRW9	A0A2J8VU83	A0A6D2WSH1	A0A2J8T9S0
Nomascus leucogenys	Gibbon	61853	17.58	G1QUR8	A0A2I3H3C5	A0A2I3GL59 Use XP_030659297	XP_030651787	A0A2I3G1F4	G1RIQ9
Macaca mulatta	Rhesus macaque	9544	27.5	H9FND6	F7GFF0	H9EV23	F6RFA8	H9FT90	A0A5K1URB0
Macaca fascicularis	Crab-eating macaque	9541	27.5	G7Q1A7	A0A2K5TN17	Use XP_045252111.1	XP_005592195	A0A2K5UEG2	A0A2K5W8I7
Papio anubis	Baboon	9555	27.5	A0A8I5MV72	A0A096NJ47	XP_003901628.1	A0A096NJ54	A0A096ML04	A0A096NWW3
Chlorocebus sabaeus	Green monkey	60711	27.5	XP_007991738.1	A0A0D9QXB9	A0A0D9RU91	A0A0D9QXB8	*A0A0D9S040*	A0A0D9S1A4
Rhinopithecus roxellana	Golden snub-nosed monk	61622	27.5	A0A2K6Q0T0	A0A2K6PT09	A0A2K6RJX1	A0A2K6RRI5	*A0A2K6RL41*	A0A2K6R611
Saimiri boliviensis	Squirrel monkey	39432	39.88	A0A2K6S1T6	A0A2K6S7B2	Use XP_039324583.1	A0A2K6TP15	A0A2K6USQ0	A0A2K6SF32
Callithrix jacchus	Marmoset	9483	39.88	U3E3K3	U3ESL0	U3ELY0	U3E989	*F7IA45CDH19*	U3FE75
Tarsier - Tarsius syrichta	Tarsier	1868482	49.61	A0A1U7T559	A0A1U7TMA9	N/A	A0A1U7STJ7	A0A1U7TPH2	N/A
Otolemur garnettii	Bushbaby	30611	57.17	XP_003801364.1	H0WJP8	H0WRC8	H0WYY4	*H0WHC4*	XP_003791346
Microcebus murinus	Mouse lemur	30608	57.17	A0A8B7HKR4	A0A8C5XY65	A0A8B7FIF0	A0A8B7ESJ7	A0A8C5XWB9	A0A8B7EU56
Mus musculus	Mouse	10090	81	P97291	P55288	Q6PFX6	P55284	B2RXP4	Q8VDK4

S2 Table. Continue

Transcript accession numbers

Species	Common name	Taxid	MYr *	Type I Classical Cadherins			
				CDH1	CDH2	CDH3	CDH4
Homo sapiens	Human	9606	0	NM_004360.5	NM_001792.5	NM_001793.6	NM_001794.5
Pan troglodytes	Chimp	9598	7.04	XM_001168150.4	XM_523898.6	XM_001168050.5	XM_016938212.2
Pan paniscus	Bonobo	9597	7.04	XM_034940548.1	XM_034943719.1	XM_003814561.4	XM_034947466.1
Gorilla gorilla	Gorilla	9595	8.09	XM_004057872.3	XM_019014146.2	XM_004057869.3	XM_031004805.1
Pongo pygmaeus abelii	Orangutan	9601	14.5	NM_001133902	NM_001132373.1	XM_002826574.4	XM_024239065.1
Nomascus leucogenys	Gibbon	61853	17.58	XM_030794861.1	XM_030810618.1	XM_004087300.3	XM_030825780.1
Macaca mulatta	Rhesus macaque	9544	27.5	XM_015126485.2	XM_015121712.2	XM_015126483.2	XM_028828556.1
Macaca fascicularis	Crab-eating macaque	9541	27.5	XM_005592359.2	XM_005586980.2	XM_005592357.2	XM_005569504.2
Papio anubis	Baboon	9555	27.5	XM_003917094.4	XM_003914260.5	XM_021932099.2	XM_021920848.2
Chlorocebus sabaeus	Green monkey	60711	27.5	XM_007993837	XM_007974381.2	XM_007993839.2	XM_008012096.1
Rhinopithecus roxellana	Golden snub-nosed monk	61622	27.5	XM_010355036.2	XM_030926084.1	XM_030925018.1	XM_030914248.1
Saimiri boliviensis	Squirrel monkey	39432	39.88	XM_010346845.1	XM_039463602.1	XM_003936335.3	XM_039479762.1
Callithrix jacchus	Marmoset	9483	39.88	XM_035281641.1	XM_002757141.4	XM_003735322.4	XM_035299088.1
Tarsier - Tarsius syrichta	Tarsier	1868482	49.61	Assembled from: KEKE944053v1 - KE947423v1 - XM_008070049	XM_021719311.1	XM_008050332.2	XM_008064945.2
Otolemur garnettii	Bushbaby	30611	57.17	N/A	XM_003784768.3	XM_023518928.1	XM_012803965.1
Microcebus murinus	Mouse lemur	30608	57.17	XM_012742400.2	XM_012737106.1	XM_020282516.1	XM_020281742.1
Mus musculus	Mouse	10090	81	NM_009864.3	NM_007664.5	NM_001037809.5	NM_009867.3

Species	Common name	Taxid	MYr *	Type II Classical Cadherins - Group A			Type II Classical Cadherins - Group B				
				CDH6	CDH9	CDH10	CDH7	CDH12	CDH18	CDH20	CDH22
Homo sapiens	Human	9606	0	NM_004932.4	NM_016279.4	NM_006727.5	NM_001362438.2	NM_004061.5	NM_004934.5	NM_031891.4	NM_021248.3
Pan troglodytes	Chimp	9598	7.04	XM_016953493.2	XM_001135234.5	XM_009449368.3	XM_001149161.4	XM_024356657.1	XM_526963.6	XM_512160.5	XM_016938033.1
Pan paniscus	Bonobo	9597	7.04	XM_034960236.1	XM_003809055.3	XM_003809057.4	XM_003827281.5	XM_034960250.1	XM_014344961.2	XM_003827263.3	XM_034947215.1
Gorilla gorilla	Gorilla	9595	8.09	XM_004058997.3	XM_004059005.3	XM_019012927.2	XM_004059526.3	XM_019012909.2	XM_019013682.2	XM_004059490.3	XM_031004925.1
Pongo pygmaeus abelii	Orangutan	9601	14.5	NM_001131774.1	XM_024247337.1	XM_009240608.2	XM_024236001.1	XM_024246642.1	XM_024247646.1	XM_024236041.1	XM_024238983.1
Nomascus leucogenys	Gibbon	61853	17.58	XM_003274942.3	XM_003263184.3	XM_003263183.2	XM_003264322.3	XM_030813934.1	XM_030813926.1	XM_003264289.4	XM_030825605.1
Macaca mulatta	Rhesus macaque	9544	27.5	XM_015139785.2	XM_015139773.2	XM_028849362.1	XM_001094033.4	XM_015139767.2	XM_001088306.4	XM_015122130.2	XM_015148909.2
Macaca fascicularis	Crab-eating macaque	9541	27.5	XM_005556634.3	XM_005556631	XM_005556626	XM_005586498.3	XM_005556619.3	XM_005556617.2	XM_005586543.3	XM_045363046.1
Papio anubis	Baboon	9555	27.5	XM_003899539.5	XM_017959463.3	XM_003899529.4	XM_021930206.2	XM_031666714.1	XM_009208188.4	XM_003914440.5	XM_021920987.2
Chlorocebus sabaeus	Green monkey	60711	27.5	XM_007961271.2	XM_007961266.2	XM_007961265.2	XM_037988043.1	XM_007961261.2	XM_007961259.2	XM_008013896.2	XM_008015408.2
Rhinopithecus roxellana	Golden snub-nosed monk	61622	27.5	XM_010378023.2	XM_010353177.2	XM_010380197.2	XM_010385602.2	XM_030927854.1	XM_010386932.2	XM_030926096.1	XM_010384183.2
Saimiri boliviensis	Squirrel monkey	39432	39.88	XM_003936203.3	XM_010331608	XM_039468492.1	XM_039463772.1	XM_010350904.2	XM_039472193.1	XM_010336754.2	XM_039479699.1
Callithrix jacchus	Marmoset	9483	39.88	XM_002745091.5	XM_002745093.4	XM_035291745.1	XM_002757315.4	XM_008992219.3	XM_035292295.1	XM_035271276.1	XM_035298744.1
Tarsier - Tarsius syrichta	Tarsier	1868482	49.61	XM_008053896.2	XM_008051757.1	XM_008055097.2	XM_008066663.2	XM_008058681.1	XM_008071385.2	XM_008057024.1	N/A
Otolemur garnettii	Bushbaby	30611	57.17	XM_012807072.1	XM_012805001.1	XM_003789535.2	XM_003788408.3	XM_003789536.2	XM_003789537.1	XM_003788420.2	XM_003787669.2
Microcebus murinus	Mouse lemur	30608	57.17	XM_012738424.1	XM_012765178.2	XM_012765179.2	XM_012748392.2	XM_020290038.1	XM_012738455.2	XM_012774498.2	XM_012742986.1
Mus musculus	Mouse	10090	81	XM_036159108.1	XM_011245337.4	XM_006520100.5	NM_001316743.1	NM_001008420.2	NM_001081299.1	NM_011800.5	XM_036157156.1

Species	Common name	Taxid	MYr *	Type II Classical Cadherins - Group C			Type II Classical Cadherins - Ungrouped		
				CDH8	CDH11	CDH24	CDH5	CDH19	CDH13
Homo sapiens	Human	9606	0	NM_001796.5	NM_001797.4	NM_022478.4	NM_001795.5	NM_021153.4	NM_001257.5
Pan troglodytes	Chimp	9598	7.04	XM_001155344.4	XM_001156354.6	XM_016925878.2	XM_523383.5	XM_009434121.3	XM_016930251.1
Pan paniscus	Bonobo	9597	7.04	XM_034940341.1	XM_034940344.1	XM_034937290.1	XM_003823693.4	XM_003827282.3	XM_003820881.3
Gorilla gorilla	Gorilla	9595	8.09	XM_019012882.2	XM_004057751.3	XM_019009511.2	XM_004057752.3	XM_019013749.2	XM_004058060.3
Pongo pygmaeus abelii	Orangutan	9601	14.5	T0168007	XM_009250776.2	XM_009248928.2	XM_009250782.2	XM_024235962.1	NM_001133193.1
Nomascus leucogenys	Gibbon	61853	17.58	XM_003263129.4	XM_003263130.4	XM_003260631.4	XM_030795927.1	XM_003264324.4	XM_003272479.3
Macaca mulatta	Rhesus macaque	9544	27.5	NM_001257905.1	NM_001266613.1	XM_028851410.1	XM_001082641.4	XM_001094512.4	JU332410
Macaca fascicularis	Crab-eating macaque	9541	27.5	XM_005592125.3	XM_005592134.2	XM_045396176.1	XM_005592138.3	XM_005586494.3	XM_005592650
Papio anubis	Baboon	9555	27.5	XM_031658175.1	XM_003916982.4	XM_003901579.5	XM_003916983.3	XM_003914465.5	XM_017953610.2
Chlorocebus sabaeus	Green monkey	60711	27.5	XM_007993547.2	XM_007993553.2	XM_007990829.2	XM_007993560.2	XM_037987989.1	XM_007994191.2
Rhinopithecus roxellana	Golden snub-nosed monk	61622	27.5	XM_010378203.2	XM_010355192.2	XM_030931568.1	XM_010355191.2	XM_010386337.2	XM_010355650.2
Saimiri boliviensis	Squirrel monkey	39432	39.88	XM_003936250.2	XM_003936252.3	XM_039468649.1	XM_010346732.2	XM_010336734.2	XM_003938055
Callithrix jacchus	Marmoset	9483	39.88	XM_002761039.4	XM_002761041.4	XM_017977001.2	XM_008985989.3	XM_002757317.4	GAMT01008223.1
Tarsier - Tarsius syrichta	Tarsier	1868482	49.61	XM_008057003.1	XM_021711664.1	N/A	XM_008050659.2	XM_008061504.2	XM_008057625.1
Otolemur garnettii	Bushbaby	30611	57.17	XM_003801316.1	XM_003799425.3	XM_003801921.2	XM_003799424.3	XM_003788407.1	XM_023514115.1
Microcebus murinus	Mouse lemur	30608	57.17	XM_012781533.1	XM_020282838.1	XM_012752778.1	XM_012742663.2	XM_012748393.2	XM_012743294.1
Mus musculus	Mouse	10090	81	NM_001039154.2	XM_006530624.3	XM_030247793.2	NM_009868.4	NM_001081386.2	NM_019707.5

* Distance to Homo based on Purvis A. A composite estimate of primate phylogeny. Philos Trans R Soc Lond B Biol Sci. 1995;348(1326):405-21. Epub 1995/06/29. doi: 10.1098/rstb.1995.0078. PubMed PMID: 7480112.
