# Supplementary material for: Classical cadherins evolutionary constraints in primates is associated with their expression in the central nervous system
Source: PLoS One. 2024 Nov 21;19(11):e0313428. doi: 10.1371/journal.pone.0313428 (PMC11581309; doi:10.1371/journal.pone.0313428)
Supplement: S3 Table — Probability of rejecting the null hypothesis of strict neutrality (dS = dN) in favor of negative selection (dN<dS). (PDF) [file pone.0313428.s003.pdf]

S3 Table. Codon-based Z-test of selection for classical cadherins between *H. sapines* and non-human primates. Probability of rejecting the null hypothesis of strict neutrality (dS = dN) in favor of negative selection (dN<dS).

	**Type I**			
***H. sapiens* vs. each species**	**CDH1**	**CDH2**	**CDH3**	**CDH4**
Pan_troglodytes	0.001	0.014	0.006	0.001
Pan_paniscus	0.000	0.014	0.031	0.000
Gorilla_gorilla_gorilla	0.000	0.005	0.000	0.000
Pongo_abelii	0.000	0.002	0.056*	0.000
Nomascus_leucogeny	0.000	0.000	0.000	0.000
Macaca_mulatta	0.000	0.000	0.000	0.000
Macaca_fascicularis	0.000	0.000	0.000	0.000
Papio_anubis	0.000	0.000	0.000	0.000
Chlorocebus_sabaeus	0.000	0.000	0.000	0.000
Rhinopithecus_roxellana	0.000	0.000	0.000	0.000
Saimiri_boliviensis_boliviensis	0.000	0.000	0.000	0.000
Callithrix_jacchus	0.000	0.000	0.000	0.000
Carlito_syrichta	0.000	0.000	0.000	0.000
Otolemur_garnettii		0.000	0.000	0.000
Microcebus_murinus	0.000	0.000	0.000	0.000

	**Type II**													
	**Group A**			**Group B**					**Group C**			**Ungrouped**		
***H. sapiens* vs. each species**	**CDH6**	**CDH9**	**CDH10**	**CDH7**	**CDH12**	**CDH18**	**CDH20**	**CDH22**	**CDH8**	**CDH11**	**CDH24**	**CDH5**	**CDH19**	**CDH13**
Pan_troglodytes	0.004	0.029	0.023	0.002	0.014	0.002	0.002	0.004	0.008	0.013	0.124*	0.008	0.085*	0.012
Pan_paniscus	0.003	0.041	0.066*	0.009	0.009	0.001	0.002	0.007	0.014	0.022	0.016	0.007	0.264*	0.004
Gorilla_gorilla_gorilla	0.002	0.004	0.005	0.004	0.017	0.011	0.001	0.000	0.005	0.020	0.001	0.009	0.062*	0.016
Pongo_abelii	0.003	0.000	0.000	0.000	0.000	0.000	0.000	0.000	0.000	0.000	0.000	0.000	0.073*	0.000
Nomascus_leucogeny	0.002	0.000	0.000	0.000	0.000	0.000	0.000	0.000	0.000	0.000	0.001	0.000	0.113*	0.000
Macaca_mulatta	0.000	0.000	0.000	0.000	0.000	0.000	0.000	0.000	0.000	0.000	0.000	0.000	0.000	0.000
Macaca_fascicularis	0.000	0.000	0.000	0.000	0.000	0.000	0.000	0.000	0.000	0.000	0.000	0.000	0.000	0.000
Papio_anubis	0.000	0.000	0.000	0.000	0.000	0.000	0.000	0.000	0.000	0.000	0.000	0.000	0.000	0.000
Chlorocebus_sabaeus	0.000	0.000	0.000	0.000	0.000	0.000	0.000	0.000	0.000	0.000	0.000	0.000	0.000	0.000
Rhinopithecus_roxellana	0.000	0.000	0.000	0.000	0.000	0.000	0.000	0.000	0.000	0.000	0.000	0.000	0.000	0.000
Saimiri_boliviensis_boliviensis	0.000	0.000	0.000	0.000	0.000	0.000	0.000	0.000	0.000	0.000	0.000	0.000	0.000	0.000
Callithrix_jacchus	0.000	0.000	0.000	0.000	0.000	0.000	0.000	0.000	0.000	0.000	0.000	0.000	0.000	0.000
Carlito_syrichta	0.000	0.000	0.000	0.000	0.000	0.000	0.000		0.000	0.000		0.000	0.000	0.000
Otolemur_garnettii	0.000	0.000	0.000	0.000	0.000	0.000	0.000	0.000	0.000	0.000	0.000	0.000	0.000	0.000
Microcebus_murinus	0.000	0.000	0.000	0.000	0.000	0.000	0.000	0.000	0.000	0.000	0.000	0.000	0.000	0.000

* p > 0.05

S3 Table. Codon-based Z-test of selection of cadherins type I and type II. The probability of rejecting the null hypothesis of strict neutrality (dN = dS) in favor of negative selection (dS < dN) are shown. p values < 0.05 are considered significant at the 5% level. The variance was computed using the analytical method and all positions containing gaps and missing data were eliminated. Evolutionary analyses were conducted using the Kumar's method in MEGA-X. [1][2][3]

The Fisher's Exact Test for sequence pairs evaluates the probability (P) of rejecting the null hypothesis of strict neutrality in favor of the alternative hypothesis of positive selection. In all cases p = 1.00.

1. Nei M. and Kumar S. (2000). Molecular Evolution and Phylogenetics. Oxford University Press, New York.
2. Kumar S., Stecher G., Li M., Knyaz C., and Tamura K. (2018). MEGA X: Molecular Evolutionary Genetics Analysis across computing platforms. Molecular Biology and Evolution 35:1547-1549.
3. Stecher G., Tamura K., and Kumar S. (2020). Molecular Evolutionary Genetics Analysis (MEGA) for macOS. Molecular Biology and Evolution (https://doi.org/10.1093/molbev/msz312).
