# Supplementary material for: Classical cadherins evolutionary constraints in primates is associated with their expression in the central nervous system
Source: PLoS One. 2024 Nov 21;19(11):e0313428. doi: 10.1371/journal.pone.0313428 (PMC11581309; doi:10.1371/journal.pone.0313428)
Supplement: S4 Table — (PDF) [file pone.0313428.s004.pdf]

S4 Table. Spearman's correlation: dN/dS (NWM to human) vs. LOEUF score

	Average dN/dS ratio (NWM to human)	LOEUF score
**CDH1**	0.216596533	0.51
**CDH2**	0.03628571	0.34
**CDH3**	0.168288425	0.82
**CDH4**	0.040968114	0.53
**CDH5**	0.290080421	0.66
**CDH6**	0.045925136	0.51
**CDH7**	0.016524959	0.65
**CDH8**	0.011118231	0.37
**CDH9**	0.057238248	0.58
**CDH10**	0.010413704	0.66
**CDH11**	0.032419177	0.25
**CDH12**	0.045764456	0.67
**CDH13**	0.058089275	0.46
**CDH18**	0.065331533	0.74
**CDH19**	0.252544733	1.1
**CDH20**	0.025425884	0.53
**CDH22**	0.051117277	0.78
**CDH24**	0.104798012	1.15

Spearman's Correlation Coefficient = 0.478, $p < 0.05$ level (2-tailed)
