# Supplementary material for: Classical cadherins evolutionary constraints in primates is associated with their expression in the central nervous system
Source: PLoS One. 2024 Nov 21;19(11):e0313428. doi: 10.1371/journal.pone.0313428 (PMC11581309; doi:10.1371/journal.pone.0313428)
Supplement: S1 File — The content of each Table is described in their heading. (PDF) [file pone.0313428.s005.pdf]

Fig 1A. Plotted Data. Amino acid sequence distance vs *H. sapiens*

	Pan troglodyte	*Pan paniscus*	*Gorilla gorilla*	*Pongo abelii*	*Nomascus leucogenys*	*Macaca mulatta*	*Macaca fascicularis*	*Papio anubis*	*Chlorocebus sabaeus*	*Rhinopithecus roxellana*	*Saimiri boliviensis*	*Callithrix jacchus*	*Tarsius syrichta*	*Otolemur garnettii*	*Microcebus murinus*
**MYr ago**	**7.0**	**7.0**	**8.1**	**14.5**	**17.6**	**27.5**	**27.5**	**27.5**	**27.5**	**27.5**	**39.9**	**39.9**	**49.6**	**57.2**	**57.2**
**CDH1-FL**	0.002681199	0.004024891	0.000001	0.016229734	0.012102827	0.039666238	0.039673481	0.041056708	0.034094199	0.027170787	0.092183046	0.064229474	0.09854966		0.128432096
**CDH2-FL**	0.002607148	0.002607148	0.001302963	0.003915148	0.001303081	0.003915148	0.003915148	0.003915148	0.003915148	0.003915148	0.006533141	0.006533141	0.015754559	0.013113779	0.011796274
**CDH3-FL**	0.004115546	0.008246152	0.009627098	0.029267281	0.034885296	0.036205853	0.037606118	0.033421006	0.036193832	0.03195997	0.055093723	0.057771557	0.08267145	0.085724916	0.076805808
**CDH4-FL**	0.002585568	0.002585568	0.001292064	0.007781984	0.015591408	0.005175333	0.00647156	0.00647156	0.005516312	0.006472136	0.014297395	0.014298591	0.030084505	0.026309851	0.032891178

Fig 1B. Plotted Data - Medians

	CDH1	CDH2	CDH3	CDH4
**FL**	0.036880218	0.003915148	0.036193832	0.006472136
**EC1**	0.018801224	0.000001	0.004653691	0.000001
**EC2**	0.033198942	0.016483099	0.016546054	0.000001
**EC3**	0.052037206	0.008203653	0.039556361	0.012060611
**EC4**	0.027123294	0.004551329	0.06234795	0.018234696
**EC5**	0.078052815	0.000001	0.078662627	0.008919381
**CD**	0.013538271	0.000001	0.027179712	0.006428134

Fig 1B. Raw Data. Amino acid sequence distance vs *H. sapiens*

	Pan troglodyte	*Pan paniscus*	*Gorilla gorilla*	*Pongo abelii*	*Nomascus leucogenys*	*Macaca mulatta*	*Macaca fascicularis*	*Papio anubis*	*Chlorocebus sabaeus*	*Rhinopithecus roxellana*	*Saimiri boliviensis*	*Callithrix jacchus*	*Tarsius syrichta*	*Otolemur garnettii*	*Microcebus murinus*
**MYr ago**	**7.0**	**7.0**	**8.1**	**14.5**	**17.6**	**27.5**	**27.5**	**27.5**	**27.5**	**27.5**	**39.9**	**39.9**	**49.6**	**57.2**	**57.2**
**CDH1-EC1**	0.000001	0.000001	0.000001	0.000001	0.018424201	0.018801224	0.018801224	0.018801224	0.018801224	0.018801224	0.055738975	0.037031086	0.076632975		0.121005769
**CDH2-EC1**	0.000001	0.000001	0.000001	0.000001	0.000001	0.000001	0.000001	0.000001	0.000001	0.000001	0.000001	0.000001	0.008994924	0.008994924	0.008994924
**CDH3-EC1**	0.000001	0.000001	0.000001	0.000001	0.009306382	0.00931242	0.000001	0.000001	0.000001	0.000001	0.037848087	0.037848087	0.077075925	0.086415205	0.086415205
**CDH4-EC1**	0.000001	0.000001	0.000001	0.000001	0.000001	0.000001	0.008925556	0.008925556	0.000001	0.000001	0.000001	0.000001	0.017873622	0.008937923	0.008937923
**CDH1-EC2**	0.000001	0.000001	0.000001	0.008208738	0.016445881	0.033198942	0.033198942	0.041634009	0.041590049	0.016506025	0.106164695	0.087032046	0.111355527		0.104061246
**CDH2-EC2**	0.008178349	0.008178349	0.008178349	0.016483099	0.000001	0.016483099	0.016483099	0.016483099	0.016483099	0.016483099	0.016483099	0.016483099	0.008227267	0.016483099	0.008227267
**CDH3-EC2**	0.016580135	0.016580135	0.008256548	0.025005382	0.016511974	0.016511974	0.016511974	0.016511974	0.016511974	0.008256548	0.04181701	0.058698662	0.042062339	0.059322444	0.077132774
**CDH4-EC2**	0.000001	0.000001	0.000001	0.000001	0.007914773	0.000001	0.000001	0.000001	0.000001	0.000001	0.007960582	0.015970086	0.01592346	0.007956433	0.015961809
**CDH1-EC3**	0.008459517	0.017003092	0.000001	0.025510861	0.025521365	0.052037206	0.052037206	0.052080831	0.043261931	0.034228206	0.097675704	0.089678021	0.123232146		0.091475073
**CDH2-EC3**	0.000001	0.000001	0.000001	0.008203653	0.000001	0.008203653	0.008203653	0.008203653	0.008203653	0.008203653	0.008203653	0.008203653	0.041560901	0.024750558	0.024896179
**CDH3-EC3**	0.000001	0.000001	0.008674281	0.035160943	0.062467082	0.026209429	0.026209429	0.026209429	0.035082653	0.04395178	0.072211621	0.053083038	0.137669087	0.12796698	0.099218103
**CDH4-EC3**	0.008023011	0.008023011	0.008023011	0.016149658	0.03241025	0.008023011	0.008023011	0.008023011	0.008023011	0.016111118	0.01609321	0.008028013	0.024195458	0.032316301	0.03255958
**CDH1-EC4**	0.008936909	0.008936909	0.000001	0.016972565	0.017943438	0.027123294	0.02715636	0.036347149	0.018008661	0.017925028	0.064638242	0.055036022	0.036366555		0.087089254
**CDH2-EC4**	0.009113892	0.009113892	0.000001	0.000001	0.009107431	0.000001	0.000001	0.000001	0.000001	0.000001	0.009101657	0.009101657	0.009115013	0.009115013	0.018267451
**CDH3-EC4**	0.000001	0.000001	0.018744859	0.028448989	0.028401163	0.057498641	0.057498641	0.067904412	0.077119158	0.067197259	0.056999062	0.076730665	0.108328773	0.140953967	0.098975915
**CDH4-EC4**	0.000001	0.000001	0.000001	0.009087555	0.027528303	0.018234696	0.018234696	0.018234696	0.018234696	0.009087555	0.03667358	0.027421865	0.027444907	0.036844534	0.046137638
**CDH1-EC5**	0.000001	0.000001	0.000001	0.038611696	0.000001	0.088354305	0.088354305	0.078052815	0.05792732	0.057933219	0.168885049	0.123240701	0.230089619		0.305453385
**CDH2-EC5**	0.000001	0.000001	0.000001	0.000001	0.000001	0.000001	0.000001	0.000001	0.000001	0.000001	0.008886113	0.008886113	0.026872455	0.00891958	0.008852651
**CDH3-EC5**	0.000001	0.009408329	0.018988895	0.079025404	0.07889952	0.078425733	0.078425733	0.068196253	0.068196253	0.048044244	0.100554386	0.089624234	0.119653698	0.099088879	0.088674819
**CDH4-EC5**	0.000001	0.000001	0.000001	0.008979961	0.008919381	0.000001	0.000001	0.000001	0.000001	0.008919381	0.008951621	0.008919381	0.045032411	0.036533999	0.054286065
**CDH1-CD**	0.000001	0.000001	0.000001	0.006776738	0.000001	0.013538271	0.013538271	0.013538271	0.013538271	0.013538271	0.051056456	0.013519113	0.041123815		0.057183759
**CDH2-CD**	0.000001	0.000001	0.000001	0.000001	0.000001	0.000001	0.000001	0.000001	0.000001	0.000001	0.000001	0.000001	0.006367445	0.012757038	0.006367445
**CDH3-CD**	0.006706485	0.013482191	0.006706485	0.020322578	0.027179712	0.033992889	0.04080348	0.027128282	0.027125131	0.027177415	0.034363667	0.041275791	0.047869185	0.03412218	0.033964323
**CDH4-CD**	0.000001	0.000001	0.000001	0.000001	0.012871201	0.006428134	0.006428134	0.006428134	0.006428134	0.006428134	0.01287862	0.019405122	0.039470866	0.033381208	0.039955833

Table 1. Satistical analysis of amino acid substitutions / site. Kruskal-Wallis followed by Dunn's test

	FL	EC1	EC2	EC3	EC4	EC5	CD
**Fig 1A, B**							
**CDH1 vs. CDH2**	0.001 **	0.006 **	0.154	0.003 *	0.037 *	0.003 **	0.006 **
**CDH1 vs. CDH3**	0.550	0.371	0.382	1	0.118	0.724	0.129
**CDH1 vs. CDH4**	0.045 *	0.018 *	< 0.001 **	0.008 *	0.533	0.029 *	0.49
**CDH2 vs.CDH3**	< 0.001	0.173	0.02 *	0.003 *	< 0.001 **	< 0.001 **	< 0.001 **
**CDH2 vs. CDH4**	0.451	0.679	0.0639	1	0.108	0.724	0.044 *
**CDH3 vs. CDH4**	0.008 **	0.323	< 0.001 **	0.009 *	0.043 *	0.003 **	0.026 *

p Adjusted: *p < 0.05; **p < 0.005

Fig 2 A, B, C, D. Plotted Data

	Pan troglodyte	*Pan paniscus*	*Gorilla gorilla*	*Pongo abelii*	*Nomascus leucogenys*	*Macaca mulatta*	*Macaca fascicularis*	*Papio anubis*	*Chlorocebus sabaeus*	*Rhinopithecus roxellana*	*Saimiri boliviensis*	*Callithrix jacchus*	*Tarsius syrichta*	*Otolemur garnettii*	*Microcebus murinus*
**MYr ago**	**7.0**	**7.0**	**8.1**	**14.5**	**17.6**	**27.5**	**27.5**	**27.5**	**27.5**	**27.5**	**39.9**	**39.9**	**49.6**	**57.2**	**57.2**
**CDH6**	0.00263088	0.00131506	0.00395066	0.01056389	0.00791656	0.00395145	0.00395145	0.00395030	0.00527014	0.00659136	0.01322122	0.00659613	0.00923251	0.03385745	0.02257285
**CDH9**	0.00000100	0.00132654	0.00000100	0.00000100	0.00132591	0.00531745	0.00531426	0.00531539	0.00664595	0.00663938	0.01197129	0.01198747	0.01869474	0.03838767	0.01869835
**CDH10**	0.00000100	0.00132412	0.00000100	0.00000100	0.00132394	0.00264909	0.00264909	0.00132416	0.00132416	0.00132416	0.00662997	0.00132825	0.00264960	0.00264853	0.00264969
**CDH7**	0.00000100	0.00000100	0.00261404	0.00130484	0.00261054	0.00261054	0.00261054	0.00261054	0.00261054	0.00130477	0.00522617	0.00391712	0.01046996	0.01047529	0.00784455
**CDH12**	0.00132403	0.00305459	0.00764975	0.00530315	0.00397709	0.00663639	0.00663639	0.00530637	0.00663578	0.00530637	0.01464245	0.01863743	0.01063751	0.01869704	0.01331424
**CDH18**	0.00263686	0.00131766	0.00263663	0.00660533	0.00793279	0.01192169	0.01192382	0.01326341	0.01326268	0.01325254	0.01325421	0.01325577	0.02605578	0.03208395	0.02271332
**CDH20**	0.00131609	0.00131609	0.00000100	0.00395058	0.00263202	0.00790947	0.00790947	0.00658957	0.00658983	0.00924970	0.00527584	0.00527365	0.00791503	0.01585675	0.01188962
**CDH22**	0.00257277	0.00257277	0.00256244	0.00514633	0.00899188	0.00771137	0.00771137	0.01818435	0.00770492	0.00641953	0.01287375	0.00641548		0.02069834	0.01815050
**CDH8**	0.00000100	0.00000100	0.00000100	0.00132045	0.00264211	0.00000100	0.00000100	0.00000100	0.00000100	0.00132016	0.00132038	0.00264178	0.00396456	0.00264178	0.00396623
**CDH11**	0.00262671	0.00131285	0.00131285	0.00131285	0.00394348	0.00526255	0.00526255	0.00526255	0.00526255	0.00526255	0.01188162	0.01188162	0.00526393	0.00923176	0.00659049
**CDH24**	0.01361116	0.01771146	0.00951423	0.00950866	0.00950962	0.02045756	0.02046428	0.01771146	0.01634122	0.02045885	0.02598330	0.02754246		0.03985444	0.04272716
**CDH5**	0.00265513	0.00265513	0.00532993	0.01199594	0.01469036	0.01332099	0.01332099	0.01466016	0.01065175	0.01198406	0.08202998	0.06193505	0.17814728	0.14822903	0.14643398
**CDH19**	0.00795914	0.00929732	0.00927430	0.02677852	0.02686285	0.02943159	0.02941521	0.02806328	0.03083293	0.03354586	0.07288073	0.05436804	0.11708585	0.18105415	0.11206581
**CDH13**	0.00000100	0.00000100	0.01012748	0.00673970	0.00843054	0.00505209	0.00505209	0.00505209	0.00673822	0.00842847	0.01182325	0.01864215		0.03429874	0.03792251

Fig 2 E, F, G, H. Plotted data - Medians

	FL	EC1	EC2	EC3	EC4	EC5	CD
**CDH6**	0.006591361	0.000001	0.000001	0.008622735	0.000001	0.007901666	0.006387454
**CDH9**	0.005317446	0.000001	0.000001	0.008551227	0.009003634	0.008096576	0.000001
**CDH10**	0.001324164	0.000001	0.000001	0.008436244	0.000001	0.000001	0.000001
**CDH7**	0.002610539	0.000001	0.000001	0.008350858	0.000001	0.000001	0.000001
**CDH12**	0.006636394	0.009645662	0.000001	0.000001	0.000001	0.007807577	0.012648516
**CDH18**	0.013252537	0.000001	0.000001	0.00845347	0.046670133	0.015794887	0.006401127
**CDH20**	0.006589572	0.000001	0.000001	0.008447531	0.009044898	0.016598722	0.006216491
**CDH22**	0.007708143	0.000001	0.000001	0.016413538	0.004594673	0.000001	0.011247622
**CDH8**	0.001320163	0.000001	0.000001	0.000001	0.000001	0.000001	0.000001
**CDH11**	0.00526255	0.000001	0.000001	0.025668303	0.009152518	0.000001	0.000001
**CDH24**	0.019084511	0.000001	0.000001	0.03082711	0.027291009	0.01804844	0.026995951
**CDH5**	0.013320993	0.018683913	0.008727887	0.017125873	0.000001	0.025198903	0.000001
**CDH19**	0.02943159	0.037817532	0.000001	0.025397849	0.057945453	0.033389999	0.025860725
**CDH13**	0.007584085	0.000001	0.000001	0.004208745	0.01815869	0.000001	

Fig 2 A to H. Satistical analysis of amino acid substitutions / site. Kruskal-Wallis followed by Dunn's test

	FL	EC1	EC2	EC3	EC4	EC5	CD
**CDH type II**							
**Fig 2A, E.**							
CDH10 vs. CDH6	0.003 **	0.678	0.444	0.001 **	0.192	0.220	0.002 **
CDH10 vs. CDH9	0.016 *	1	0.939	0.137	0.007 **	0.023 *	0.116
CDH6 vs. CDH9	0.527	0.678	0.939	0.055	0.192	0.286	0.111
**Fig 2B, F.**							
CDH12 vs. CDH18	0.899	< 0.001 **	0.578	1	< 0.001 **	0.489	1
CDH12 vs. CDH20	0.899	< 0.001 **	0.943	1	1	0.518	0.252
CDH12 vs. CDH22	0.899	< 0.001 **	0.731	0.001 **	1	1	1
CDH12 vs. CDH7	0.0549	< 0.001 **	0.578	1	1	1	0.006 *
CDH18 vs. CDH20	0.143	1	1	1	0.003 **	1	0.899
CDH18 vs. CDH22	0.878	1	0.016 *	0.007 **	0.003 **	0.44	1
CDH18 vs. CDH7	< 0.001 **	1	1	1	< 0.001 **	0.121	0.0805
CDH20 vs. CDH22	0.899	1	0.071	0.006 **	1	0.495	0.857
CDH20 vs. CDH7	0.608	1	1	1	0	0.18	0.87
CDH22 vs. CDH7	0.0904	1	0.016 *	0.019 *	1	1	0.046 *
**Fig 2C, G.**							
CDH11 vs CDH24	0.002**	0.249	0.261	0.0655	0.002 **	< 0.001 **	< 0.001 **
CDH11 vs CDH8-FL	0.016 *	0.249	0.451	< 0.001 **	0.012 **	0.313	0.443
CDH24 vs CDH8-FL	< 0.001 **	1	0.63	< 0.001 **	< 0.001 **	<0.001 **	< 0.001 **
**Fig 2D, H.**							
CDH13 vs CDH19-FL	0.003 **	< 0.001 **	0.006 *	< 0.001 **	0.002 **	0.003 **	
CDH13 vs CDH5-FL	0.0579	0.0572	< 0.001 **	< 0.001 **	0.884	0.001 **	
CDH19 vs CDH5-FL	0.274	0.0589	0.318	0.864	0.002 **	0.557	0.057

p Adjusted: *p < 0.05; **p < 0.005

Fig 2 E, F, G H. Raw Data

	Pan troglodyte	*Pan paniscus*	*Gorilla gorilla*	*Pongo abelii*	*Nomascus leucogenys*	*Macaca mulatta*	*Macaca fascicularis*	*Papio anubis*	*Chlorocebus sabaeus*	*Rhinopithecus roxellana*	*Saimiri boliviensis*	*Callithrix jacchus*	*Tarsius syrichta*	*Otolemur garnettii*	*Microcebus murinus*
**MYr ago**	7.0	7.0	8.1	14.5	17.6	27.5	27.5	27.5	27.5	27.5	39.9	39.9	49.6	57.2	57.2
**CDH6_EC1**	0.00953136	0.00000100	0.00000100	0.00000100	0.00000100	0.00000100	0.00000100	0.00000100	0.00000100	0.00000100	0.00000100	0.00000100	0.00000100	0.00000100	0.00000100
**CDH9_EC1**	0.00000100	0.00000100	0.00000100	0.00000100	0.00000100	0.00000100	0.00000100	0.00000100	0.00000100	0.00000100	0.00000100	0.00000100	0.00000100	?	0.00000100
**CDH10_EC1**	0.00000100	0.00000100	0.00000100	0.00000100	0.00000100	0.00000100	0.00000100	0.00000100	0.00000100	0.00000100	0.00000100	0.00000100	0.00000100	0.00000100	0.00000100
**CDH7_EC1**	0.00000100	0.00000100	0.00000100	0.00000100	0.00000100	0.00000100	0.00000100	0.00000100	0.00000100	0.00000100	0.00000100	0.00000100	0.00943178	0.00000100	0.00000100
**CDH12_EC1**	0.00964566	0.05209790	0.05209790	0.00964566	0.00964566	0.00964566	0.00964566	0.00964566	0.00964566	0.00964566	0.00964566	0.00964566	0.00964566	0.01932594	0.01935051
**CDH18_EC1**	0.00000100	0.00000100	0.00000100	0.00000100	0.00000100	0.00000100	0.00000100	0.00000100	0.00000100	0.00000100	0.00000100	0.00000100	0.00000100	0.00000100	0.00000100
**CDH20_EC1**	0.00000100	0.00000100	0.00000100	0.00000100	0.00000100	0.00000100	0.00000100	0.00000100	0.00000100	0.00000100	0.00000100	0.00000100	0.00000100	0.00000100	0.00000100
**CDH22_EC1**	0.00000100	0.00000100	0.00000100	0.00000100	0.00000100	0.00000100	0.00000100	0.00000100	0.00000100	0.00000100	0.00000100	0.00000100		0.00000100	0.00000100
**CDH8_EC1**	0.00000100	0.00000100	0.00000100	0.00000100	0.00000100	0.00000100	0.00000100	0.00000100	0.00000100	0.00000100	0.00000100	0.00000100	0.00000100	0.00000100	0.00000100
**CDH11_EC1**	0.00000100	0.00000100	0.00000100	0.00000100	0.00000100	0.00000100	0.00000100	0.00000100	0.00000100	0.00000100	0.00944843	0.00944843	0.00000100	0.00000100	0.00000100
**CDH24_EC1**	0.00000100	0.00000100	0.00000100	0.00000100	0.00000100	0.00000100	0.00000100	0.00000100	0.00000100	0.00000100	0.00000100	0.00000100		0.00000100	0.00000100
**CDH5_EC1**	0.00000100	0.00000100	0.00000100	0.00000100	0.01868391	0.01868391	0.01868391	0.01868391	0.01868391	0.01868391	0.07673549	0.05721648	0.17413713	0.16037194	0.18323245
**CDH19_EC1**	0.01878269	0.01878269	0.01878269	0.05789288	0.03805698	0.02825051	0.03779577	0.03781753	0.01874192	0.04740277	0.04828597	0.04774341	0.13144157	0.15284023	0.08837328
**CDH13_EC1**	0.00000100	0.00000100	0.00000100	0.00000100	0.00902977	0.00000100	0.00000100	0.00000100	0.00000100	0.00000100	0.01812282	0.02742160		0.03710154	0.03710154
**CDH6_EC2**	0.00000100	0.00000100	0.00000100	0.00870148	0.00000100	0.00000100	0.00000100	0.00000100	0.00000100	0.00000100	0.00000100	0.00000100	0.00000100	0.15189417	0.00000100
**CDH9_EC2**	0.00000100	0.00000100	0.00000100	0.00000100	0.00000100	0.00000100	0.00000100	0.00000100	0.00000100	0.01736324	0.00000100	0.00000100	0.00000100	0.00000100	0.00000100
**CDH10_EC2**	0.00000100	0.00000100	0.00000100	0.00000100	0.00000100	0.00000100	0.00000100	0.00000100	0.00000100	0.00000100	0.00000100	0.00000100	0.00000100	0.00000100	0.00000100
**CDH7_EC2**	0.00000100	0.00000100	0.00000100	0.00000100	0.00000100	0.00000100	0.00000100	0.00000100	0.00000100	0.00000100	0.00000100	0.00000100	0.00000100	0.00000100	0.00000100
**CDH12_EC2**	0.00000100	0.00000100	0.00000100	0.00000100	0.00000100	0.00000100	0.00000100	0.00000100	0.00000100	0.00000100	0.00000100	0.00000100	0.00906673	0.01820869	0.00906673
**CDH18_EC2**	0.00000100	0.00000100	0.00000100	0.00000100	0.00000100	0.00000100	0.00000100	0.00000100	0.00000100	0.00000100	0.00000100	0.00000100	0.00000100	0.00000100	0.00000100
**CDH20_EC2**	0.00000100	0.00000100	0.00000100	0.00000100	0.00000100	0.00000100	0.00000100	0.00000100	0.00000100	0.00000100	0.00000100	0.00000100	0.00000100	0.00880527	0.00000100
**CDH22_EC2**	0.00000100	0.00000100	0.00000100	0.00000100	0.00873519	0.00876265	0.00876265	0.00000100	0.00000100	0.00000100	0.00871808	0.00000100		0.00877370	0.00877370
**CDH8_EC2**	0.00000100	0.00000100	0.00000100	0.00000100	0.00000100	0.00000100	0.00000100	0.00000100	0.00000100	0.00000100	0.00000100	0.00886202	0.00886202	0.00000100	0.00000100
**CDH11_EC2**	0.00000100	0.00000100	0.00000100	0.00000100	0.00000100	0.00000100	0.00000100	0.00000100	0.00000100	0.00000100	0.00000100	0.00000100	0.00000100	0.00000100	0.00000100
**CDH24_EC2**	0.00000100	0.00000100	0.00000100	0.00885920	0.00000100	0.00000100	0.00000100	0.00000100	0.00000100	0.00000100	0.00000100	0.00000100		0.00884612	0.00884612
**CDH5_EC2**	0.00000100	0.00000100	0.00000100	0.00872789	0.00872789	0.00872789	0.00872789	0.00872789	0.00872789	0.00870504	0.10286480	0.09277200	0.16544086	0.14322830	0.15599851
**CDH19_EC2**	0.00000100	0.00000100	0.00000100	0.00000100	0.00899337	0.00000100	0.00000100	0.00000100	0.00000100	0.01807526	0.01830949	0.00911820	0.04580541	0.04579432	0.01803148
**CDH13_EC2**	0.00000100	0.00000100	0.00825482	0.00000100	0.00000100	0.00000100	0.00000100	0.00000100	0.00000100	0.00000100	0.00000100	0.00000100		0.01659379	0.02502431
**CDH6_EC3**	0.00862273	0.00862273	0.01728978	0.04373305	0.01728978	0.00862273	0.00862273	0.01727935	0.01727935	0.01730456	0.00862273	0.01737222	0.02597798	0.01728978	0.00862273
**CDH9_EC3**	0.00000100	0.00000100	0.00000100	0.00000100	0.00000100	0.01721954	0.00855123	0.00855123	0.00855123	0.00000100	0.01707856	0.02572629	0.02571736	0.08877690	0.03456718
**CDH10_EC3**	0.00000100	0.00000100	0.00000100	0.00000100	0.00000100	0.01689406	0.01689406	0.00843067	0.00843067	0.00843067	0.01686158	0.00841710	0.00843624	0.00841741	0.00841741
**CDH7_EC3**	0.00000100	0.00000100	0.00000100	0.00000100	0.00000100	0.00835086	0.00835086	0.00835086	0.00835086	0.00835086	0.00000100	0.00000100	0.01675828	0.00835059	0.01674350
**CDH12_EC3**	0.00000100	0.00000100	0.00000100	0.00858376	0.00000100	0.00000100	0.00000100	0.00000100	0.00000100	0.00000100	0.01724555	0.00860102	0.00000100	0.00860102	0.00000100
**CDH18_EC3**	0.00000100	0.00000100	0.00000100	0.00000100	0.00000100	0.00000100	0.00000100	0.00000100	0.00000100	0.00000100	0.00858656	0.00859906	0.02589837	0.01727713	0.00861755
**CDH20_EC3**	0.00847044	0.00847044	0.00000100	0.00000100	0.00000100	0.00000100	0.00000100	0.00000100	0.00000100	0.00849186	0.00000100	0.00000100	0.01698955	0.00846053	0.00844753
**CDH22_EC3**	0.00818727	0.00818727	0.00818727	0.00818727	0.01638441	0.01641354	0.01641354	0.01641354	0.01641354	0.01641354	0.02468030	0.01633476		0.03301459	0.04133308
**CDH8_EC3**	0.00000100	0.00000100	0.00000100	0.00838562	0.00000100	0.00000100	0.00000100	0.00000100	0.00000100	0.00837460	0.00000100	0.00000100	0.00000100	0.00837460	0.00837460
**CDH11_EC3**	0.00845347	0.00845347	0.00845347	0.00845347	0.00845347	0.02566830	0.02566830	0.02566830	0.02566830	0.02566830	0.05198867	0.05198867	0.02567432	0.02567432	0.02567432
**CDH24_EC3**	0.02636882	0.03532658	0.01751889	0.02636018	0.01751425	0.03532658	0.03532658	0.03532658	0.03532658	0.02636018	0.03530368	0.05358717		0.03525943	0.02639479
**CDH5_EC3**	0.00000100	0.00000100	0.00000100	0.01710494	0.01730194	0.01712587	0.01712587	0.02576904	0.01712587	0.01712587	0.16885027	0.07057023	0.29184693	0.26478234	0.23621351
**CDH19_EC3**	0.00000100	0.00000100	0.00834024	0.04281769	0.01664801	0.02545288	0.02539785	0.01686748	0.01686748	0.03401485	0.06919100	0.05984425	0.08681629	0.16106798	0.10556271
**CDH13_EC3**	0.00000100	0.00000100	0.00843586	0.00000100	0.00844249	0.00000100	0.00000100	0.00000100	0.00841649	0.00000100	0.00000100	0.00000100		0.02552057	0.00000100
**CDH6_EC4**	0.00000100	0.00000100	0.00000100	0.00000100	0.01806110	0.00000100	0.00000100	0.00000100	0.00000100	0.00000100	0.01808345	0.00900490	0.00899966	0.01806180	0.01806180
**CDH9_EC4**	0.00000100	0.00000100	0.00000100	0.00000100	0.00000100	0.00900363	0.01806493	0.00900363	0.02718819	0.00900363	0.00900363	0.01806635	0.06430654	0.05495853	0.07331403
**CDH10_EC4**	0.00000100	0.00000100	0.00000100	0.00000100	0.00000100	0.00000100	0.00000100	0.00000100	0.00000100	0.00000100	0.00000100	0.00000100	0.00904373	0.00000100	0.00906744
**CDH7_EC4**	0.00000100	0.00000100	0.00000100	0.00000100	0.00000100	0.00869964	0.00869964	0.00869964	0.00869964	0.00000100	0.00869964	0.01743281	0.00869964	0.00000100	0.00000100
**CDH12_EC4**	0.00000100	0.00000100	0.00000100	0.00000100	0.00000100	0.00000100	0.00000100	0.00000100	0.00000100	0.00000100	0.00000100	0.02780317	0.00927476	0.01861814	0.00927476
**CDH18_EC4**	0.00916699	0.00000100	0.00916258	0.01837949	0.01837949	0.04667013	0.04672809	0.05651977	0.04672809	0.05619113	0.04667013	0.04667013	0.09660555	0.08551936	0.08738128
**CDH20_EC4**	0.00000100	0.00000100	0.00000100	0.00907009	0.00904490	0.00904496	0.00904496	0.00904496	0.00904496	0.01823645	0.00000100	0.00000100	0.00000100	0.00905357	0.02746094
**CDH22_EC4**	0.00000100	0.00000100	0.00000100	0.00000100	0.00000100	0.00918835	0.00918835	0.01850905	0.00918835	0.00918835	0.00921522	0.00000100		0.00919825	0.00000100
**CDH8_EC4**	0.00000100	0.00000100	0.00000100	0.00000100	0.00000100	0.00000100	0.00000100	0.00000100	0.00000100	0.00000100	0.00000100	0.00000100	0.00000100	0.00000100	0.00887926
**CDH11_EC4**	0.00000100	0.00000100	0.00000100	0.00000100	0.00919991	0.00915252	0.00915252	0.00915252	0.00915252	0.00915252	0.00915415	0.00915415	0.00916652	0.02760768	0.00916652
**CDH24_EC4**	0.01813433	0.02729101	0.01813433	0.01813433	0.02731997	0.03647453	0.02729101	0.02729101	0.02729101	0.03650496	0.02728598	0.02732298		0.06446387	0.08397581
**CDH5_EC4**	0.00000100	0.00000100	0.00000100	0.00000100	0.00000100	0.00000100	0.00000100	0.00000100	0.00000100	0.00950379	0.03862757	0.02883014	0.15652299	0.13365077	0.17676914
**CDH19_EC4**	0.01904508	0.01904508	0.01906283	0.04804177	0.06805319	0.05794545	0.04804177	0.04804177	0.04831128	0.07784405	0.15314582	0.14244160	0.15291845	0.25623391	0.19791717
**CDH13_EC4**	0.00000100	0.00000100	0.00906613	0.02731575	0.01815869	0.01815869	0.01815869	0.01815869	0.01815869	0.02729102	0.02744558	0.02744558		0.02744558	0.04612562
**CDH6_EC5**	0.00000100	0.00000100	0.00000100	0.00000100	0.00000100	0.00790167	0.00790167	0.00000100	0.00790167	0.00790167	0.01583342	0.00790167	0.01583422	0.02394563	0.01587103
**CDH9_EC5**	0.00000100	0.00000100	0.00000100	0.00000100	0.00810895	0.00809658	0.00809658	0.00809658	0.00809658	0.01622909	0.02436880	0.00809837	0.00816416	0.03290532	0.00816416
**CDH10_EC5**	0.00000100	0.00000100	0.00000100	0.00000100	0.00817875	0.00000100	0.00000100	0.00000100	0.00000100	0.00000100	0.00818579	0.00000100	0.00000100	0.00817676	0.00000100
**CDH7_EC5**	0.00000100	0.00000100	0.00803674	0.00000100	0.00801320	0.00000100	0.00000100	0.00000100	0.00000100	0.00000100	0.00801076	0.00801076	0.01602643	0.03225677	0.02415875
**CDH12_EC5**	0.00000100	0.00782139	0.00780524	0.00780758	0.00780758	0.00780758	0.00780758	0.00780758	0.01565346	0.00780758	0.03154360	0.03954822	0.00781724	0.01566379	0.00000100
**CDH18_EC5**	0.00000100	0.00000100	0.00000100	0.01577898	0.01579489	0.01579489	0.01579489	0.01579489	0.01579489	0.01579489	0.02377073	0.01579489	0.03189040	0.02383667	0.02384245
**CDH20_EC5**	0.00000100	0.00000100	0.00000100	0.01659208	0.00000100	0.01659872	0.01659872	0.00828902	0.00828902	0.01665448	0.01670549	0.02510879	0.01661592	0.05031874	0.03344792
**CDH22_EC5**	0.00832361	0.00832361	0.00832361	0.00832361	0.01675759	0.00000100	0.00000100	0.00000100	0.00000100	0.00000100	0.00832361	0.00832361		0.00831723	0.00832361
**CDH8_EC5**	0.00000100	0.00000100	0.00000100	0.00000100	0.01619772	0.00000100	0.00000100	0.00000100	0.00000100	0.00000100	0.00807437	0.00807437	0.01619482	0.00808947	0.00808117
**CDH11_EC5**	0.00780745	0.00000100	0.00000100	0.00000100	0.00000100	0.00000100	0.00000100	0.00000100	0.00000100	0.00000100	0.00000100	0.00000100	0.00000100	0.00786450	0.00786450
**CDH24_EC5**	0.01807573	0.01804844	0.01807804	0.00897343	0.00899756	0.02719461	0.03642371	0.01804844	0.01804844	0.01804844	0.03633820	0.05524807		0.07446107	0.07394251
**CDH5_EC5**	0.01674348	0.01674348	0.02558835	0.03367338	0.02519890	0.02519890	0.02519890	0.02519890	0.02519890	0.02519890	0.08819826	0.07032250	0.16732181	0.19293287	0.16418519
**CDH19_EC5**	0.00828271	0.01669583	0.00000100	0.01660806	0.00000100	0.03339000	0.03339000	0.03339000	0.04231081	0.02517487	0.07671998	0.05937684	0.13478895	0.24225401	0.14143509
**CDH13_EC5**	0.00000100	0.00000100	0.00864575	0.00000100	0.00000100	0.00000100	0.00000100	0.00000100	0.00000100	0.00864575	0.00864575	0.02609512		0.01733860	0.02629282
**CDH6_CD**	0.00000100	0.00000100	0.00638745	0.01280501	0.01284026	0.00638745	0.00638745	0.00638745	0.00638745	0.01279609	0.03231046	0.00638745	0.00638745	0.01921654	0.07911501
**CDH9_CD**	0.00000100	0.00649479	0.00000100	0.00000100	0.00000100	0.00000100	0.00000100	0.00648287	0.00000100	0.00000100	0.01950824	0.01958107	0.00647382	0.01949529	0.00000100
**CDH10_CD**	0.00000100	0.00649773	0.00000100	0.00000100	0.00000100	0.00000100	0.00000100	0.00000100	0.00000100	0.00000100	0.00649748	0.00000100	0.00000100	0.00000100	0.00000100
**CDH7_CD**	0.00000100	0.00000100	0.00633998	0.00000100	0.00000100	0.00000100	0.00000100	0.00000100	0.00000100	0.00000100	0.01266651	0.00000100	0.01268792	0.01911090	0.00631963
**CDH12_CD**	0.00000100	0.00000100	0.01899415	0.00630419	0.00630419	0.01901904	0.01901904	0.01264852	0.01264852	0.01264852	0.01903130	0.01895816	0.01900915	0.03207202	0.03189159
**CDH18_CD**	0.00637104	0.00637104	0.00637041	0.00640113	0.01284242	0.00640113	0.00640113	0.00640113	0.01286804	0.00640113	0.00640113	0.01283019	0.00640113	0.05879669	0.01926241
**CDH20_CD**	0.00000100	0.00000100	0.00000100	0.00000100	0.00621649	0.01872221	0.01872221	0.01871892	0.01872221	0.01246054	0.01245775	0.00620743	0.00621533	0.01243387	0.00000100
**CDH22_CD**	0.00000100	0.00000100	0.00000100	0.01135095	0.01119015	0.01124762	0.01124762	0.05872073	0.01688417	0.01124762	0.02259718	0.01123569		0.04007006	0.03472660
**CDH8_CD**	0.00000100	0.00000100	0.00000100	0.00000100	0.00000100	0.00000100	0.00000100	0.00000100	0.00000100	0.00000100	0.00000100	0.00000100	0.00000100	0.00000100	0.00000100
**CDH11_CD**	0.00000100	0.00000100	0.00000100	0.00000100	0.00644871	0.00000100	0.00000100	0.00000100	0.00000100	0.00000100	0.00645261	0.00645261	0.00000100	0.00000100	0.00000100
**CDH24_CD**	0.01347108	0.02699595	0.00672039	0.00000100	0.00670726	0.02699595	0.02699595	0.02699595	0.02019960	0.04077553	0.02714284	0.02773534		0.04066711	0.04798766
**CDH5_CD**	0.00000100	0.00000100	0.00000100	0.00629292	0.01896004	0.00000100	0.00000100	0.00000100	0.00000100	0.00000100	0.04462304	0.05818669	0.13877460	0.05790665	0.02541798
**CDH19_CD**	0.00000100	0.00000100	0.01282388	0.01286548	0.02666606	0.02586072	0.02586072	0.02586072	0.03240563	0.01286958	0.07319626	0.02594456	0.11702474	0.18230773	0.07341479

Fig 3A, B. Plotted data

		Pan troglodyte	*Pan paniscus*	*Gorilla gorilla*	*Pongo abelii*	*Nomascus leucogenys*	*Macaca mulatta*	*Macaca fascicularis*	*Papio anubis*	*Chlorocebus sabaeus*	*Rhinopithecus roxellana*	*Saimiri boliviensis*	*Callithrix jacchus*	*Tarsius syrichta*	*Otolemur garnettii*	*Microcebus murinus*
	MYr ago	7.0	7.0	8.1	14.5	17.6	27.5	27.5	27.5	27.5	27.5	39.9	39.9	49.6	57.2	57.2
**dS**	CDH1 FL	0.02682385	0.03086406	0.02709894	0.06474863	0.03777578	0.08229064	0.08222200	0.07662265	0.07445917	0.07900289	0.15586454	0.15688798	0.30614072		0.32436012
**dS**	CDH2 FL	0.01228552	0.01228138	0.01259572	0.02142899	0.02770819	0.03881734	0.03645585	0.03915644	0.04354795	0.03544336	0.08240842	0.06425377	0.19655808	0.23732904	0.19096042
**dS**	CDH3 FL	0.01437708	0.01414847	0.01304073	0.04841712	0.05673779	0.08574040	0.08722594	0.09777380	0.08815994	0.08732807	0.13055880	0.13492582	0.28901734	0.28457050	0.33384063
**dS**	CDH4 FL	0.02035576	0.02308479	0.02940854	0.07026581	0.06356709	0.09329301	0.09107558	0.10766721	0.10350209	0.10573389	0.19066250	0.18132799	0.38574665	0.35909314	0.36508594
**dN**	CDH1 FL	0.00128176	0.00185367	0.00000000	0.00815444	0.00613140	0.01793392	0.01793982	0.01831110	0.01495157	0.01248539	0.04270018	0.03365719	0.05053467		0.06903323
**dN**	CDH2 FL	0.00129324	0.00129324	0.00064620	0.00300247	0.00064683	0.00182259	0.00182259	0.00182259	0.00182273	0.00182259	0.00299918	0.00352774	0.00760441	0.00512497	0.00583958
**dN**	CDH3 FL	0.00193537	0.00401340	0.01440368	0.00513040	0.01692908	0.01807661	0.01822618	0.01723053	0.01837403	0.01595123	0.02787817	0.02808764	0.04190465	0.04362395	0.04054623
**dN**	CDH4 FL	0.00129786	0.00129786	0.00064830	0.00379003	0.00772298	0.00259825	0.00325099	0.00325204	0.00277972	0.00313721	0.00618015	0.00683585	0.01504216	0.01606882	0.01861368

Fig 3 C. Plotted data

		Means dN/dS					ST error dN/dS			
		CDH1 FL	CDH2 FL	CDH3 FL	CDH4 FL		CDH1 FL	CDH2 FL	CDH3 FL	CDH4 FL
	MYr ago									
**Apes**	10.85	0.09902346	0.085065403	0.38542583	0.063491339		0.024321967	0.020972673	0.183851582	0.016165174
**OWM**	27.5	0.206787736	0.047354459	0.197417472	0.030151782		0.01360668	0.001651817	0.00741872	0.001768044
**NWM**	48.742	0.216596533	0.03628571	0.168288425	0.040968114		0.019939349	0.00537627	0.018163705	0.003180945

Fig 3C. Raw Data

		Pan troglodyte	*Pan paniscus*	*Gorilla gorilla*	*Pongo abelii*	*Nomascus leucogenys*	*Macaca mulatta*	*Macaca fascicularis*	*Papio anubis*	*Chlorocebus sabaeus*	*Rhinopithecus roxellana*	*Saimiri boliviensis*	*Callithrix jacchus*	*Tarsius syrichta*	*Otolemur garnettii*	*Microcebus murinus*
**dN/dS**	CDH1 FL	0.047784346	0.060059282		0.125939941	0.162310272	0.217933952	0.218187579	0.238977695	0.200802324	0.158037129	0.273957006	0.214530097	0.165070074		0.212828957
**dN/dS**	CDH2 FL	0.105265695	0.105301138	0.051303438	0.140112367	0.02334438	0.04695303	0.04999448	0.04654641	0.041855727	0.051422646	0.036394112	0.054903261	0.038687865	0.026837881	0.024605432
**dN/dS**	CDH3 FL	0.134614842	0.283663207	1.104514548	0.105962621	0.298373929	0.210829535	0.20895365	0.176228519	0.20841695	0.182658705	0.213529625	0.208171021	0.144990091	0.153297528	0.121453861
**dN/dS**	CDH4 FL	0.063758831	0.056221413	0.022044563	0.053938468	0.12149342	0.027850448	0.035695482	0.03020459	0.026856609	0.029670775	0.032414106	0.037698836	0.038994918	0.044748336	0.050984373

Fig 3D. Plotted data

**dN (ED-CD)**	CDH1	0.001632304	0.002365412	0	0.007014263	0.007787687	0.016160515	0.016169531	0.0166437	0.012352423	0.009194609	0.036065043	0.035394367	0.040663913		0.047090663
**dN (ED-CD)**	CDH2	0.00173536	0.00173536	0.000866927	0.00316457	0.000867679	0.00244957	0.00244957	0.00244957	0.002449824	0.00244957	0.004032566	0.004745352	0.007105067	0.003771223	0.004729078
**dN (ED-CD)**	CDH3	-0.000768427	-0.003114927	0.00347407	0.007478279	0.006586747	0.001152261	0.0047516	0.007677794	0.008313312	0.00509875	0.00949929	0.01019558	0.027645836	0.032723191	0.033544772
**dN (ED-CD)**	CDH4	0.000868433	0.000868433	0.000868433	0.003335654	0.001490325	-0.000501728	0.000372388	0.000367543	-0.000250556	0.000224088	0.000197193	-0.002937397	-0.001836389	-0.007738166	-0.007654381

Fig 3D. Raw Data

**dN**	CDH1 ED	0.001632304	0.002365412	0	0.009624489	0.007787687	0.021382377	0.021391393	0.021865562	0.017574285	0.014416471	0.049268223	0.041399542	0.059415133		0.079268893
**dN**	CDH2 ED	0.00173536	0.00173536	0.000866927	0.00316457	0.000867679	0.00244957	0.00244957	0.00244957	0.002449824	0.00244957	0.004032566	0.004745352	0.009524745	0.00618793	0.00714584
**dN**	CDH3 ED	0.001828156	0.002744746	0.006070652	0.015969606	0.018379222	0.018214983	0.019167869	0.018792914	0.020105788	0.016891225	0.030218723	0.030579131	0.049543634	0.047821281	0.052282796
**dN**	CDH4 ED	0.000868433	0.000868433	0.000868433	0.003335654	0.007720935	0.002608702	0.003482818	0.003482818	0.002859874	0.003334518	0.005810476	0.005812153	0.014088524	0.014427195	0.01710314
**dN**	CDH1 CD	0	0	0	0.002610226	0	0.005221862	0.005221862	0.005221862	0.005221862	0.005221862	0.01320318	0.006005175	0.01875122		0.03217823
**dN**	CDH2 CD	0	0	0	0	0	0	0	0	0	0	0	0	0.002419678	0.002416707	0.002416761
**dN**	CDH3 CD	0.002596582	0.005859673	0.002596582	0.008491326	0.011792475	0.017062723	0.014416269	0.01111512	0.011792475	0.011792475	0.020719432	0.020383551	0.021897798	0.01509809	0.018738024
**dN**	CDH4 CD	0	0	0	0	0.00623061	0.00311043	0.00311043	0.003115275	0.00311043	0.00311043	0.005613283	0.00874955	0.015924913	0.02216536	0.024757522

Fig 3. Statistical analysis of pairwise comparisons by Kruskal-Wallis and Dunn's test (p. adjusted)

	dS FL	dN FL		dN/dS FL		dN ED-CD	
	p	p	Sig	p	Sig	p	Sig
**CDH type I**							
**CDH1 vs. CDH2**	0.67	0.001	***	< 0.001	***	0.039	*
**CDH1 vs. CDH3**	1	0.672	ns	1	ns	0.301	ns
**CDH1 vs. CDH4**	0.996	0.052	ns	< 0.001	****	< 0.001	****
**CDH2 vs. CDH3**	0.416	< 0.001	****	< 0.001	***	0.257	ns
**CDH2 vs. CDH4**	0.101	0.239	ns	1.000	ns	0.039	*
**CDH3 vs .CDH4**	1	0.015	*	< 0.001	****	< 0.001	***

* p < 0.05; ** p < 0.005

**Fig 4 A to H. dS, dN, dN/dS analysis between FL protein coding nucleotide sequences. Pairwise comparisons to *H. sapiens***																
		Pan troglodyte	*Pan paniscus*	*Gorilla gorilla*	*Pongo abelii*	*Nomascus leucogenys*	*Macaca mulatta*	*Macaca fascicularis*	*Papio anubis*	*Chlorocebus sabaeus*	*Rhinopithecus roxellana*	*Saimiri boliviensis*	*Callithrix jacchus*	*Tarsius syrichta*	*Otolemur garnettii*	*Microcebus murinus*
	MYr ago	7.0	7.0	8.1	14.5	17.6	27.5	27.5	27.5	27.5	27.5	39.9	39.9	49.6	57.2	57.2
dS	CDH6	0.012837765	0.011542444	0.019200387	0.024369365	0.023052959	0.044228724	0.041444552	0.038981377	0.040273015	0.037871783	0.120932403	0.07188066	0.17981374	0.27844831	0.279640039
dS	CDH9	0.009168718	0.009159373	0.017049213	0.030384122	0.03714962	0.076617131	0.07791113	0.064968244	0.065037608	0.067568956	0.117211288	0.130948703	0.194795281	0.336505479	0.237952906
dS	CDH10	0.007484525	0.004918467	0.011318472	0.022624261	0.036911196	0.058232031	0.058232031	0.067934572	0.066161028	0.059572405	0.092350759	0.10244478	0.199894862	0.346673677	0.226196616
dS	CDH7	0.011524578	0.010224789	0.016425834	0.035175438	0.042546591	0.077486778	0.081347749	0.077496113	0.081719107	0.069602657	0.130742508	0.120973005	0.215632577	0.523396782	0.262526825
dS	CDH12	0.010133702	0.014774198	0.01802062	0.031787768	0.033472586	0.056834824	0.056834824	0.059538923	0.065111234	0.055490403	0.125874845	0.121703183	0.219475735	0.296874447	0.201719648
dS	CDH18	0.01562629	0.01562629	0.015203989	0.029004327	0.039729669	0.067723443	0.063753984	0.070624662	0.068430904	0.067760501	0.115545863	0.111098929	0.185550337	0.283971379	0.172032735
dS	CDH20	0.016507905	0.017820288	0.012965894	0.035935306	0.048538083	0.066271263	0.064849768	0.065533152	0.06638581	0.063703499	0.093035644	0.111486059	0.224300274	0.234876859	0.212316418
dS	CDH22	0.014390446	0.013105298	0.038800607	0.043739022	0.0323887	0.063171776	0.063082436	0.067997649	0.064361191	0.054808922	0.09342666	0.095879672		0.229701028	0.153207493
dS	CDH8	0.010169787	0.008861784	0.008971086	0.024350774	0.034597855	0.056894391	0.056888423	0.05556209	0.050055632	0.056912766	0.091251052	0.100083266	0.192657099	0.228168434	0.18721595
dS	CDH11	0.013747271	0.01112695	0.008842189	0.034532435	0.028210319	0.046937494	0.051124387	0.056698523	0.05294283	0.048083064	0.10384852	0.091585683	0.214877687	0.265593448	0.250000205
dS	CDH24	0.012327635	0.01751227	0.025216319	0.046076681	0.029747765	0.06020327	0.061589151	0.064463886	0.063697315	0.061798493	0.100948666	0.099320851		0.278372478	0.225458157
dS	CDH5	0.013847045	0.015146869	0.018737523	0.044002113	0.063626543	0.094307871	0.094275099	0.100229446	0.08700598	0.088789412	0.119133999	0.124641803	0.264054723	0.302023622	0.285218
dS	CDH19	0.00918374	0.00654174	0.01189067	0.022632144	0.021283643	0.053522275	0.056340604	0.053622597	0.064631888	0.061946216	0.11732646	0.106980352	0.235758108	0.364526118	0.225304
dS	CDH13	0.009974475	0.012929267	0.016787348	0.044724937	0.068765791	0.06297893	0.056506642	0.061341863	0.062583123	0.070646529	0.117014506	0.111384398	0.278559274	0.248045855	0.258749
dN	CDH6	0.00133556	0.000667557	0.001869706	0.005218251	0.003743938	0.001869401	0.001869401	0.001868671	0.003074551	0.003743607	0.005760423	0.003074162	0.004557624	0.020449131	0.011308289
dN	CDH9	0	0.000661813	0	0	0.000523941	0.002096983	0.00157275	0.002097533	0.002898673	0.003039896	0.005566615	0.004900081	0.009359316	0.039555866	0.00849096
dN	CDH10	0	0	0	0	0.000534055	0.001198225	0.001198225	0.000533302	0.000533302	0.000533728	0.002801932	0.000533301	0.001329346	0.001333034	0.00136334
dN	CDH7	0	0.001195072	0.001327581	0.00066335	0.001327141	0.001328463	0.001328463	0.001328463	0.001328463	0.001328022	0.002525276	0.001992035	0.004789528	0.005467317	0.003724149
dN	CDH12	0.00066291	0.000859845	0.003090008	0.002656931	0.001991373	0.003050854	0.003050854	0.002521004	0.00318717	0.002520622	0.006785983	0.008787516	0.009639241	0.008649747	0.00598135
dN	CDH18	0.001204125	0.000536555	0.00120462	0.003214688	0.003215585	0.005638213	0.00563778	0.005638213	0.00563778	0.006310162	0.006316913	0.006313917	0.015251977	0.015656186	0.013388351
dN	CDH20	0.000529628	0.001187902	0	0.001717556	0.00131579	0.003827367	0.003827367	0.003297078	0.003166211	0.004359486	0.00237724	0.00225003	0.004324673	0.008147339	0.005823181
dN	CDH22	0.001304632	0.001304632	0.001297859	0.002613531	0.004557323	0.004079685	0.004079685	0.005942062	0.002895992	0.002896413	0.006042875	0.003666273		0.010435929	0.008597661
dN	CDH8	0	0	0	0.00066313	0.001195519	0	0	0	0	0.00066357	0.00066335	0.001328463	0.001994021	0.001991458	0.00298983
dN	CDH11	0.001185056	0.000528508	0.000528508	0.000528508	0.001714158	0.002499816	0.002499816	0.002499816	0.002499537	0.002498889	0.005943169	0.005942079	0.002536876	0.004224064	0.003069074
dN	CDH24	0.006220924	0.004964157	0.004396641	0.004275448	0.006883218	0.008863837	0.008863837	0.008171604	0.007479768	0.009554988	0.012591204	0.013609651		0.019430248	0.019758407
dN	CDH5	0.001331116	0.001868494	0.003072702	0.006166676	0.008123064	0.006168603	0.00616918	0.006841641	0.004956119	0.005625995	0.041264576	0.031773309	0.090110905	0.076852555	0.072273
dN	CDH19	0.003859219	0.00438781	0.004242563	0.01280547	0.013242354	0.015318156	0.014784613	0.014781316	0.016320158	0.016820547	0.03252465	0.025351253	0.060034879	0.090363717	0.055424
dN	CDH13	0	0	0.002922004	0.003543185	0.003548335	0.002152556	0.002920048	0.002773423	0.002152556	0.003690297	0.004066337	0.010203706	0.011627927	0.015512196	0.015475
dN/dS	CDH6	0.104033694	0.057834965	0.097378552	0.214131579	0.162405964	0.04226667	0.045106071	0.047937537	0.0763427	0.098849514	0.04763341	0.042767582	0.025346361	0.073439595	0.040438732
dN/dS	CDH9		0.07225532			0.014103548	0.027369636	0.020186465	0.032285516	0.04456919	0.044989534	0.047492138	0.037419854	0.048046934	0.117548952	0.035683363
dN/dS	CDH10					0.014468648	0.020576739	0.020576739	0.007850223	0.008060659	0.008959322	0.030340103	0.005205746	0.006650226	0.003845213	0.006027232
dN/dS	CDH7		0.116879883	0.08082276	0.01885833	0.031192647	0.017144384	0.016330668	0.017142319	0.016256456	0.019080048	0.019314881	0.016466769	0.022211524	0.010445837	0.014185786
dN/dS	CDH12	0.065416398	0.058199127	0.171470682	0.083583436	0.059492665	0.0536793	0.0536793	0.04234212	0.048949613	0.04542446	0.053910557	0.072204488	0.043919392	0.029136045	0.029651798
dN/dS	CDH18	0.077057656	0.034336699	0.079230549	0.110834782	0.080936607	0.083253483	0.088430246	0.079833479	0.082386468	0.093124495	0.054670179	0.056831485	0.082198593	0.055132972	0.077824439
dN/dS	CDH20	0.032083267	0.066660106		0.047795776	0.02710841	0.057753036	0.059018972	0.050311597	0.047694091	0.068434006	0.025551924	0.020182166	0.019280728	0.034687705	0.027426898
dN/dS	CDH22	0.09065961	0.099549984	0.033449458	0.059752843	0.140707198	0.064580814	0.064672276	0.087386293	0.044995936	0.052845641	0.064680414	0.038238273		0.045432663	0.056117759
dN/dS	CDH8				0.027232403	0.034554722					0.011659425	0.007269505	0.013273578	0.010350102	0.008728019	0.015969953
dN/dS	CDH11	0.086202996	0.047498032	0.059771197	0.015304689	0.060763512	0.053258406	0.048896745	0.044089616	0.047212	0.051970261	0.057229213	0.064880001	0.011806139	0.015904247	0.012276287
dN/dS	CDH24	0.504632405	0.283467338	0.174356952	0.092789835	0.231386039	0.147231823	0.143918808	0.126762513	0.117426735	0.154615223	0.12472878	0.137027123		0.069799457	0.08763669
dN/dS	CDH5	0.096129941	0.123358448	0.163986549	0.140144988	0.12766785	0.065409202	0.065438065	0.068259789	0.056962966	0.063363351	0.346371117	0.254916956	0.341258447	0.254458756	0.253396832
dN/dS	CDH19	0.420222981	0.670740411	0.35679768	0.565808976	0.622184539	0.286201516	0.262414882	0.275654604	0.252509374	0.271534703	0.27721496	0.236971113	0.254646085	0.24789367	0.245997838
dN/dS	CDH13			0.174059901	0.079221681	0.051600294	0.034178986	0.051676191	0.045212562	0.034395151	0.052236076	0.034750707	0.091608038	0.041743097	0.062537615	0.059806916

Fig 4 I, J, K, L. Plotted Data								
		Average				S.E.		
	MYr ago	10.9	27.5	48.7		10.9	27.5	48.7
dN/dS	CDH6	0.127156951	0.062100499	0.045925136		0.027417198	0.011036556	0.00782151
dN/dS	CDH9	0.043179434	0.033880068	0.057238248		0.018389205	0.004848302	0.015287625
dN/dS	CDH10	0.014468648	0.013204737	0.010413704		0	0.003015367	0.00500367
dN/dS	CDH7	0.061938405	0.017190775	0.016524959		0.020291836	0.000509244	0.002031644
dN/dS	CDH12	0.087632462	0.048814959	0.045764456		0.021444072	0.002244269	0.008077624
dN/dS	CDH18	0.076479258	0.085405634	0.065331533		0.012214887	0.002382489	0.006043577
dN/dS	CDH20	0.04341189	0.05664234	0.025425884		0.007974289	0.00364743	0.002784428
dN/dS	CDH22	0.084823819	0.062896192	0.051117277		0.018223264	0.007166555	0.005209908
dN/dS	CDH11	0.053908085	0.049085406	0.032419177		0.011523119	0.001646807	0.011774165
dN/dS	CDH8	0.030893562	0.011659425	0.011118231		0.002315521		0.001570041
dN/dS	CDH11	0.053908085	0.049085406	0.032419177		0.011523119	0.001646807	0.011774165
dN/dS	CDH24	0.257326514	0.13799102	0.104798012		0.069452402	0.006877007	0.014036526
dN/dS	CDH5	0.130257555	0.063886675	0.290080421		0.011077916	0.001898166	0.021953235
dN/dS	CDH19	0.527150917	0.269663016	0.252544733		0.059832093	0.005743288	0.006782579
dN/dS	CDH13	0.101627292	0.043539793	0.058089275		0.028724893	0.003974127	0.009894776

Fig 4 M, N, L, O, P. Raw data																
		Pan troglodyte	*Pan paniscus*	*Gorilla gorilla*	*Pongo abelii*	*Nomascus leucogenys*	*Macaca mulatta*	*Macaca fascicularis*	*Papio anubis*	*Chlorocebus sabaeus*	*Rhinopithecus roxellana*	*Saimiri boliviensis*	*Callithrix jacchus*	*Tarsius syrichta*	*Otolemur garnettii*	*Microcebus murinus*
	Myr ago	7.0	7.0	8.1	14.5	17.6	27.5	27.5	27.5	27.5	27.5	39.9	39.9	49.6	57.2	57.2
dN ED	CDH6	0.001755158	0.000877193	0.001756699	0.0052791	0.003518044	0.001755928	0.001755928	0.001755158	0.003341767	0.003338954	0.004221585	0.003340852	0.005291055	0.023856006	0.004047038
dN ED	CDH9	0	0	0	0	0.000694149	0.002776835	0.002082663	0.002082663	0.00383487	0.0040205	0.004714375	0.004716968	0.009725008	0.049112621	0.010354977
dN ED	CDH10	0	0	0	0	0.000709785	0.001589265	0.001589265	0.000708158	0.000708158	0.000708659	0.002126987	0.000708408	0.00176134	0.001767517	0.001808062
dN ED	CDH7	0	0.001597465	0.000884565	0	0.000884956	0.001772266	0.001772266	0.001772266	0.001772266	0.000884956	0.001770697	0.002657225	0.004801702	0.004807497	0.004087138
dN ED	CDH12	0.000882223	0.001269036	0.001013173	0.002650183	0.001765227	0.001765227	0.001765227	0.001765227	0.002650183	0.001764448	0.005666727	0.008147617	0.009427319	0.007088722	0.003181579
dN ED	CDH18	0.000870322	0	0.000701204	0.003319138	0.003320279	0.005778665	0.005778665	0.005779158	0.005778665	0.00665547	0.007367878	0.006660565	0.018607251	0.012806435	0.013661433
dN ED	CDH20	0.000709161	0.001594021	0	0.002303183	0.000883392	0.002654873	0.002654873	0.002654873	0.001768348	0.004076374	0.001592876	0.002303186	0.004210085	0.008447451	0.00693832
dN ED	CDH22	0.001784123	0.001784123	0.001784123	0.001784919	0.004472302	0.003247019	0.003247019	0.003086667	0.002355059	0.002355453	0.005041866	0.002517621		0.00580574	0.005950714
dN ED	CDH8	0	0	0	0.000879508	0.001589612	0	0	0	0	0.000880282	0.000879895	0.001762116	0.002646676	0.002646244	0.003972869
dN ED	CDH11	0.001573672	0.000702681	0.000702681	0.000702681	0.001406233	0.003318335	0.003318335	0.003318335	0.003318088	0.003318088	0.007018217	0.007018217	0.003370205	0.0056145	0.004078783
dN ED	CDH24	0.005723437	0.004969854	0.004970137	0.005725603	0.008202042	0.008333206	0.008333206	0.007407532	0.007407532	0.007575971	0.010353874	0.011882637		0.017771806	0.017754847
dN ED	CDH5	0.001792117	0.002509568	0.003225985	0.005937894	0.008205172	0.006659181	0.006659181	0.007565141	0.006659181	0.007562698	0.049572144	0.034061177	0.096800837	0.092461262	0.090958098
dN ED	CDH19	0.004506565	0.005227671	0.004138018	0.015575753	0.011932486	0.015487192	0.014756141	0.014756141	0.014216684	0.020113231	0.032501752	0.029121163	0.055607582	0.082216027	0.053481048
dN CD	CDH6	0	0	0.002531007	0.005778499	0.00506719	0.002531007	0.002531007	0.002531007	0.002531007	0.005778499	0.012331141	0.002531007	0.002534211	0.011259823	0.040487434
dN CD	CDH9	0	0.003205139	0	0	0	0	0	0.002490067	0	0	0.009693357	0.006441313	0.003210284	0.013076385	0
dN CD	CDH10	0	0	0	0	0	0	0	0	0	0	0.002524624	0	0	0	0
dN CD	CDH7	0	0	0.00312501	0	0	0	0	0	0	0.00312501	0.005578051	0	0.005573135		0.00312501
dN CD	CDH12	0	0	0.008770678	0.003139728	0.003139728	0.0080662	0.0080662	0.00560053	0.005603533	0.00560053	0.008768817	0.009478957	0.008751737	0.015584079	0.01373735
dN CD	CDH18	0.002532822	0.002532822	0.003236257	0.003241502	0.003241502	0.003241502	0.003241502	0.003241502	0.003241502	0.003241502	0.003241502	0.0057879	0.004878087	0.025309436	0.01145713
dN CD	CDH20	0	0	0	0	0.00304879	0.008620427	0.008620427	0.006116284	0.008620596	0.006116284	0.005539053	0.00250088	0.002480576	0.005548461	0
dN CD	CDH22	0	0	0	0.005665783	0.005540223	0.007269708	0.007269708	0.015950808	0.005011702	0.005011702	0.010073407	0.007797377		0.020845379	0.01548339
dN CD	CDH8	0	0	0	0	0	0	0	0	0	0	0	0	0	0	0
dN CD	CDH11	0	0	0	0	0.003144664	0	0	0	0	0	0.003144664	0.003144664	0	0	0
dN CD	CDH24	0.008662741	0.005601385	0.003039523	0	0.003039523	0.011731273	0.011731273	0.011731273	0.0086445	0.017389562	0.012251762	0.01793887		0.018492092	0.020032912
dN CD	CDH5	0	0	0	0.005542072	0.009216851	0	0	0	0	0	0.017403664	0.026320502	0.070724479	0.033806615	0.012348936
dN CD	CDH19	0	0		0.006536041	0.014881816	0.012364661	0.012380989	0.012364661	0.015717839	0.006536041	0.031030051	0.012348709	0.065300555	0.037158255	0.089873081

Fig 4 M, N, O, P Plotted Data dN (ED-CD)																
		Pan troglodyte	*Pan paniscus*	*Gorilla gorilla*	*Pongo abelii*	*Nomascus leucogenys*	*Macaca mulatta*	*Macaca fascicularis*	*Papio anubis*	*Chlorocebus sabaeus*	*Rhinopithecus roxellana*	*Saimiri boliviensis*	*Callithrix jacchus*	*Tarsius syrichta*	*Otolemur garnettii*	*Microcebus murinus*
	Myr ago	7.0	7.0	8.1	14.5	17.6	27.5	27.5	27.5	27.5	27.5	39.9	39.9	49.6	57.2	57.2
dN (ED-CD)	CDH6	0.00175516	0.00087719	-0.0007743	-0.0004994	-0.0015491	-0.0007751	-0.0007751	-0.0007758	0.00081076	-0.0024395	-0.0081096	0.00080985	0.00275684	0.01259618	-0.0364404
dN (ED-CD)	CDH9	0	-0.0032051	0	0	0.00069415	0.00277684	0.00208266	-0.0004074	0.00383487	0.0040205	-0.004979	-0.0017243	0.00651472	0.03603624	0.01035498
dN (ED-CD)	CDH10	0	0	0	0	0.00070979	0.00158926	0.00158926	0.00070816	0.00070816	0.00070866	-0.0003976	0.00070841	0.00176134	0.00176752	0.00180806
dN (ED-CD)	CDH7	0	0.001597465	-0.002240446	0	0.000884956	0.001772266	0.001772266	0.001772266	0.001772266	-0.002240054	-0.003807354	0.002657225	-0.000771433	0.004807497	0.000962128
dN (ED-CD)	CDH12	0.000882223	0.001269036	-0.007757505	-0.000489545	-0.001374501	-0.006300973	-0.006300973	-0.003835303	-0.00295335	-0.003836082	-0.00310209	-0.00133134	0.000675582	-0.008495357	-0.01055577
dN (ED-CD)	CDH18	-0.0016625	-0.002532822	-0.002535053	7.76353E-05	7.8777E-05	0.002537163	0.002537163	0.002537655	0.002537163	0.003413967	0.004126376	0.000872665	0.013729163	-0.012503001	0.002204303
dN (ED-CD)	CDH20	0.000709161	0.001594021	0	0.002303183	-0.002165397	-0.005965553	-0.005965553	-0.003461411	-0.006852248	-0.00203991	-0.003946176	-0.000197694	0.001729508	0.002898989	0.011517898
dN (ED-CD)	CDH22	0.001784123	0.001784123	0.001784123	-0.003880864	-0.001067921	-0.004022689	-0.004022689	-0.012864141	-0.002656642	-0.002656249	-0.005031541	-0.005279756	0	-0.015039639	-0.012760073
dN (ED-CD)	CDH8	0	0	0	0.000879508	0.001589612	0	0	0	0	0.000880282	0.000879895	0.001762116	0.002646676	0.002646244	0.003972869
dN (ED-CD)	CDH11	0.001573672	0.000702681	0.000702681	0.000702681	-0.001738431	0.003318335	0.003318335	0.003318335	0.003318088	0.003318088	0.003873552	0.003873552	0.003370205	0.0056145	0.004078783
dN (ED-CD)	CDH24	-0.002939304	-0.000631531	0.001930614	0.005725603	0.005162519	-0.003398067	-0.003398067	-0.004323741	-0.001236968	-0.009813591	-0.001897888	-0.006056233	0	-0.000720287	-0.002278065
dN (ED-CD)	CDH5	0.001792117	0.002509568	0.003225985	0.000395822	-0.001011679	0.006659181	0.006659181	0.007565141	0.006659181	0.007562698	0.03216848	0.007740675	0.026076358	0.058654647	0.078609162
dN (ED-CD)	CDH19	0.004506565	0.005227671	0.004138018	0.009039713	-0.002949329	0.00312253	0.002375152	0.002391479	-0.001501155	0.013577191	0.001471701	0.016772453	-0.009692974	0.045057771	-0.036392033

Fig 4. Statistical analysis of pairwise comparisons Kruskal-Wallis followed by Dunn's test (p. adjusted).							
	**dS FL**	**dN FL**		**dN/dS FL**		**dN ED-CD**	
	p	p	Sig	p	Sig	p	Sig
CDH10 vs. CDH6	1	< 0.001	***	< 0.001	****	0.268	ns
CDH10 vs. CDH9	1	0.02	*	0.0234	*	0.889	ns
CDH6 vs. CDH9	1	0.2	ns	0.0234	*	0.268	ns
CDH12 vs. CDH18	1	1	ns	0.281	ns	0.00829	**
CDH12 vs. CDH20	1	1	ns	0.446	ns	0.558	ns
CDH12 vs. CDH22	1	1	ns	0.589	ns	1	ns
CDH12 vs. CDH7	1	0.267	ns	0.0138	*	0.0262	*
CDH18 vs. CDH20	1	0.3	ns	0.0037	**	0.558	ns
CDH18 vs. CDH22	1	1	ns	0.589	ns	0.0133	*
CDH18 vs. CDH7	1	0.0116	*	< 0.001	****	1	ns
CDH20 vs. CDH22	1	1	ns	0.1	ns	0.558	ns
CDH20 vs. CDH7	1	1	ns	0.446	ns	0.622	ns
CDH22 vs. CDH7	1	0.208	ns	0.000984	***	0.0383	*
CDH11 vs. CDH24	1	0.00134	**	0.00265	**	0.000346	***
CDH11 vs. CDH8	1	0.0273	*	0.00481	**	0.0781	ns
CDH24 vs. CDH8	1	< 0.001	****	< 0.001	****	0.0781	ns
CDH13 vs. CDH19	0.889	0.00112	**	< 0.001	****		
CDH13 vs. CDH5	0.889	0.0452	*	0.00586	**		
CDH19 vs. CDH5	0.56	0.201	ns	0.0119	*	0.101	ns
* p < 0.05; ** p < 0.005							

Fig 5A. CDH Type I dS, dN, and dN/dS values, dS amd dN were calculated in MEGA using Kumar - Kimura's model and analytical method						
		**MYr ago**	**dN/dS**			
Compared to *H. sapiens*			**CDH1**	**CDH2**	**CDH3**	**CDH4**
Pan troglodyte		7.0	0.047784346	0.105265695	0.13461484	0.063758831
Pan paniscus		7.0	0.060059282	0.105301138	0.28366321	0.056221413
Gorilla gorilla		8.1	0	0.051303438	1.10451455	0.022044563
Pongo abelii		14.5	0.125939941	0.140112367	0.10596262	0.053938468
Nomascus leucogenys		17.6	0.162310272	0.02334438	0.29837393	0.12149342
Macaca mulatta		27.5	0.217933952	0.04695303	0.21082953	0.027850448
Macaca fascicularis		27.5	0.218187579	0.04999448	0.20895365	0.035695482
Papio anubis		27.5	0.238977695	0.04654641	0.17622852	0.03020459
Chlorocebus sabaeus		27.5	0.200802324	0.041855727	0.20841695	0.026856609
Rhinopithecus roxellana		27.5	0.158037129	0.051422646	0.18265871	0.029670775
Saimiri boliviensis		39.9	0.273957006	0.036394112	0.21352963	0.032414106
Callithrix jacchus		39.9	0.214530097	0.054903261	0.20817102	0.037698836
Tarsius syrichta		49.6	0.165070074	0.038687865	0.14499009	0.038994918
Otolemur garnettii		57.2		0.021594377	0.15329753	0.044748336
Microcebus murinus		57.2	0.212828957	0.030580073	0.12145386	0.050984373

**Fig 5A. Gene: *SLC7A6***					
Solute carrier family 7 member 6 transporter. Located upstream of CDH3/CDH1					
	Accession #	Myr ago			
Compared to *H. sapiens*	NM_001076785.3		**dS**	**dN**	**dN/dS**
Pan troglodyte	XM_016928940.2	7.0	0.00743508	0.0000000000	0
Pan paniscus	XM_034940536.1	7.0	0.00743508	0.0000000000	0
Gorilla gorilla	XM_019011811.2	8.1	0.00743508	0.0000000000	0
Pongo abelii	XM_009250848.2	14.5	0.01809547	0.0045753570	0.252845491
Nomascus leucogenys	XM_004087302.3	17.6	0.03083622	0.0028395722	0.092085611
Macaca mulatta	XM_015126467.2	27.5	0.05365203	0.0088493709	0.164940087
Macaca fascicularis	XM_045382704.1	27.5	0.05164987	0.0088493709	0.171333843
Papio anubis	XM_009196751.3	27.5	0.05288551	0.0088493709	0.167330714
Chlorocebus sabaeus	XM_038007991.1	27.5	0.05362350	0.0115678418	0.215723375
Rhinopithecus roxellana	XM_030924666.1	27.5	0.05164987	0.0107653860	0.208430064
Saimiri boliviensis	XM_039478183.1	39.9	0.07695230	0.0169023864	0.21964758
Callithrix jacchus	XM_008986114.3	39.9	0.06786091	0.0159255795	0.234679715
Tarsius syrichta	XM_021716910.1	49.6	0.16289029	0.0394935964	0.242455186
Otolemur garnettii					
Microcebus murinus	XM_012742361.2	57.2	0.12145888	0.0490349580	0.40371652

**Fig 5A. Gene: *SNTB2***					
Syntrophin beta 2. Located downstream of CDH3/CDH1					
	Accession #				
Compared to *H. sapiens*	NP_006741.1	Myr ago	**dS**	**dN**	**dNdS**
Pan troglodyte	XP_024205663.1_	7.0	0.0084895156	0.0007603815	0.089567128
Pan paniscus	XP_034796453.1	7.0	0.0084895156	0.0007603815	0.089567128
Gorilla gorilla	XP_030858485.1	8.1	0.0235123372	0.0018544296	0.078870491
Pongo abelii	XP_002826628.3	14.5	0.0282489822	0.0007638669	0.027040509
Nomascus leucogenys	XP_003262984.3	17.6	0.0341300654		
Macaca mulatta	XP_001100304.2	27.5	0.0485100452	0.0018656738	0.038459536
Macaca fascicularis	XP_045237454.1	27.5	0.0522274433	0.0026307885	0.050371766
Papio anubis	XP_003917151.1	27.5	0.0484622443	0.0018656738	0.03849747
Chlorocebus sabaeus	XP_007992010.1	27.5	0.0521345504	0.0018656738	0.035785747
Rhinopithecus roxellana	XP_030780731.1	27.5	0.0484799262	0.0018656738	0.038483429
Saimiri boliviensis	XP_039333625.1	39.9	0.0983220668	0.0054142455	0.055066433
Callithrix jacchus	XP_035137513.1	39.9	0.0874592561	0.0052471048	0.059994848
Tarsius syrichta					
Otolemur garnettii					
Microcebus murinus	XP_020138411.1	57.2	0.2408213103	0.0617201779	0.25629035

**Fig 5A. Gene: *HRH3***					
Histamine receptor 3. Located downstream of CDH4					
	Accession #				
Compared to *H. sapiens*	XP_005260323.1	Myr ago	**dS**	**dN**	**dNdS**
Pan troglodyte	XP_016793724.1	7.0	0.0087390780	0.001113586	0.127426051
Pan paniscus	XP_008961447.2	7.0	0.0087390780	0.001113586	0.127426051
Gorilla gorilla					
Pongo abelii	XP_024094744.1	8.1	0.0218665178	0.005403735	0.247123715
Nomascus leucogenys	XP_030682402.1	14.5	0.0307307652	0.004561035	0.148419181
Macaca mulatta	XP_015004121.1	17.6	0.1102223574	0.007553757	0.068531985
Macaca fascicularis	XP_005569577.1	27.5	0.1039201621	0.007622126	0.073345976
Papio anubis	XP_009214078.1	27.5	0.1073423891	0.007621207	0.070999047
Chlorocebus sabaeus	XP_008010145.1	27.5	0.1101940560	0.007621207	0.069161691
Rhinopithecus roxellana	XP_030771317.1	27.5	0.1152205050	0.006336672	0.054996043
Saimiri boliviensis	XP_039335170.1	27.5	0.1501660730	0.0107439	0.071546784
Callithrix jacchus					
Tarsius syrichta					
Otolemur garnettii	XP_003787806.1	57.2	0.2823583995	0.024662822	0.087345807
Microcebus murinus	XP_012628357.1	57.2	0.2227882049	0.019921284	0.089418035

**Fig 5A. Gene: *TMEM241***					
Transmembrane protein 241. Located upstream of CDH2					
	Accession #				
Compared to *H. sapiens*	NM_001318834.2	Myr ago	**dS**	**dN**	**dNdS**
Pan troglodyte	XM_016933440.2	7.0	0.03348	0.02565	0.766171624
Pan paniscus	XM_034943451.1	7.0	0.02989	0.02780	0.930197981
Gorilla gorilla	XM_031003667.1	8.1	0.01782	0.01838	1.031672231
Pongo abelii	XM_024236170.1	14.5	0.03649	0.01349	0.369695083
Nomascus leucogenys					
Macaca mulatta	XM_015121665.2	17.6	0.07098	0.04493	0.632936812
Macaca fascicularis	XM_005587038.2	27.5	0.06351	0.04689	0.738420654
Papio anubis	XM_009192520.3	27.5	0.06128	0.04057	0.661963112
Chlorocebus sabaeus	XM_007974462.1	27.5	0.10276	0.09649	0.938975281
Rhinopithecus roxellana	XM_010383238.1	27.5	0.06094	0.03319	0.544643769
Saimiri boliviensis	XM_010335622.1	27.5	0.12605	0.07917	0.628084173
Callithrix jacchus					
Tarsius syrichta	XM_008060775.2	39.9	0.15861	0.06044	0.381046094
Otolemur garnettii	XM_023510172.1	57.2	0.17377	0.05685	0.327133828
Microcebus murinus	XM_012746101.1	39.9	0.14690	0.03273	0.222808233

Fig 5B. CDH Type II dS, dN, and dN/dS values, dS amd dN were calculated in MEGA using Kumar - Kimura's model and analytical method

			dN/dS											
Compared to *H. sapiens*		MYr ago	**CDH7**	**CDH19**	**CDH20**	**CDH22**	**CDH5**	**CDH8**	**CDH11**	**CDH13**	**CDH6**	**CDH9**	**CDH12**	**CDH18**
Pan troglodyte		7.0	0	0.420222981	0.032083267	0.132487993	0.096129941	0	0.086202996	0	0.104033694	0	0.065416398	0.077057656
Pan paniscus		7.0	0.116879883	0.670740411	0.066660106	0.152689807	0.123358448	0	0.047498032	0	0.057834965	0.07225497	0.058199127	0.034336699
Gorilla gorilla		8.1	0.08082276	0.35679768	0	0.041727486	0.163986549	0	0.059771197	0.174059901	0.097378552	0	0.171470682	0.079230549
Pongo abelii		14.5	0.01885833	0.565808976	0.047795776	0.039206799	0.140144988	0.027232403	0.015304689	0.079221681	0.214131579	0	0.083583436	0.110834782
Nomascus leucogenys		17.6	0.031192647	0.622184539	0.02710841	0.139515522	0.12766785	0.034554722	0.060763512	0.051600294	0.162405964	0.014103509	0.059492665	0.080936607
Macaca mulatta		27.5	0.017144384	0.286201516	0.057753036	0.058371188	0.065409202	0	0.053258406	0.034178986	0.04226667	0.027369597	0.0536793	0.083253483
Macaca fascicularis		27.5	0.016330668	0.262414882	0.059018972	0.058523754	0.065438065	0	0.048896745	0.051676191	0.045106071	0.020186461	0.0536793	0.088430246
Papio anubis		27.5	0.017142319	0.275654604	0.050311597	0.055526434	0.068259789	0	0.044089616	0.045212562	0.047937537	0.032285467	0.04234212	0.079833479
Chlorocebus sabaeus		27.5	0.016256456	0.252509374	0.047694091	0.039013838	0.056962966	0	0.047212	0.034395151	0.0763427	0.044569135	0.048949613	0.082386468
Rhinopithecus roxellana		27.5	0.019080048	0.271534703	0.068434006	0.051482643	0.063363351	0.011659425	0.051970261	0.052236076	0.098849514	0.044989593	0.04542446	0.093124495
Saimiri boliviensis		39.9	0.019314881	0.27721496	0.025551924	0.054449367	0.346371117	0.007269505	0.057229213	0.034750707	0.04763341	0.047492097	0.053910557	0.054670179
Callithrix jacchus		39.9	0.016466769	0.236971113	0.020182166	0.026220554	0.254916956	0.013273578	0.064880001	0.091608038	0.042767582	0.037419845	0.072204488	0.056831485
Tarsius syrichta		49.6	0.022211524	0.254646085	0.019280728		0.341258447	0.010350102	0.011806139	0.041743097	0.025346361	0.048046955	0.043919392	0.082198593
Otolemur garnettii		57.2	0.010445837	0.245997838	0.034687705	0.035158728	0.254458756	0.008728019	0.015904247	0.062537615	0.073439595	0.035683363	0.029136045	0.055132972
Microcebus murinus		57.2	0.014185786	0.24789367	0.027426898	0.025672058	0.253396832	0.015969953	0.012276287	0.059806916	0.040438732	0.117548962	0.029651798	0.077824439

Fig 5B. Gene: *CD226*

CD226 Molecule. Located near CDH7/CDH19/CDH20

	Accession #				
Compared to *Homo sapiens*	NM_001303618.2	MYr ago	**dS**	**dN**	**dN/dS**
Pan troglodyte	XM_016933866.2	7.0	0.055833855	0.058561812	1.048858486
Pan paniscus	XM_034944013.1	7.0	0.006698211	0.010230403	1.527333513
Gorilla gorilla	XM_031003580.1	8.1	0.003330243	0.022098373	
Pongo abelii	XM_024235979.1	14.5	0.010081463	0.02900824	2.877384034
Nomascus leucogenys	XM_003264329.3	17.6	0.017035519	0.023764632	1.395004843
Macaca mulatta	NM_001042643.1	27.5	0.045792766	0.040660107	0.887915494
Macaca fascicularis	XM_045377785.1	27.5	0.097477021	0.093809277	0.962373249
Papio anubis	XM_009192900.4	27.5	0.053523519	0.04206073	0.785836408
Chlorocebus sabaeus	XM_008013813.2	27.5	0.048299264	0.045975371	0.951885542
Rhinopithecus roxellana		27.5			
Saimiri boliviensis	XM_003925999.2	39.9	0.110782928	0.102666854	0.926738948
Callithrix jacchus	XM_035271323.1	39.9	0.109055859	0.109765249	1.006504836
Tarsius syrichta	XM_008067863.1	49.6	0.165770017	0.212845236	1.283979093
Otolemur garnettii	XM_012779996.1	57.2	0.209374221	0.218547152	1.043811176
Microcebus murinus	XM_003788458.3	57.2	0.213096432	0.2637038	1.237485762

Fig 5B. Gene: *OCSTAMP*

Osteoclast stimulatory transmembrane protein. Located downstream CDH22

	Accession #				
Compared to *H. sapiens*	NM_080721.3	MYr ago	**dS**	**dN**	**dN/dS**
Pan troglodyte	XM_003954472.3	7.0	0.007901512	0.006188088	0.783152363
Pan paniscus	XM_003826004.3	7.0	0.007896835	0.007142331	0.904454938
Gorilla gorilla	XM_004062303.3	8.1	0.0114896	0.013388759	1.165293735
Pongo abelii	XM_003780655.3	14.5	0.015473209	0.03301773	2.133864397
Nomascus leucogenys	XM_003253691.4	17.6	0.014195529	0.038850455	2.736809173
Macaca mulatta	XM_015148869.2	27.5	0.0187137	0.055854268	2.98467267
Macaca fascicularis	XM_015429916.2	27.5	0.018676824	0.055876128	2.991736098
Papio anubis	XM_021920976.2	27.5	0.030825033	0.054166796	1.75723398
Chlorocebus sabaeus	XM_008015192.2	27.5	0.026138024	0.051899354	1.985588317
Rhinopithecus roxellana	XM_010384169.2	27.5	0.0279959	0.062885606	2.24624343
Saimiri boliviensis	XM_003936482.3	39.9	0.051554309	0.094368357	1.830464979
Callithrix jacchus	XM_017973102.2	39.9	0.058851365	0.104671146	1.77856785
Tarsius syrichta	XM_008071693.2	49.6	0.103989784	0.187678078	1.80477419
Otolemur garnettii	XM_012742893.1	57.2	0.063057363	0.165425502	2.623412944
Microcebus murinus	XM_023511980.1	57.2	0.104826262	0.197549198	1.884539185

**Fig 5B. Gene: *CMTM1***					
CKLF like MARVEL transmembrane domain containing 1 Located downstream of CDH5,CDH8,CDH11					
	Accession #				
Compared to *H. sapiens*	NM_052999.4_	MYr ago	**dS**	**dN**	**dN/dS**
Pan troglodyte	XM_009430941.4	7.0	0.0102251400	0.0047638870	0.4658994427
Pan paniscus	XM_008977345.6	7.0	0.0102305956	0.0032848163	0.3210777163
Gorilla gorilla	XM_019012442.1	8.1	0.0136236606	0.0128754998	0.9450837143
Pongo abelii	XM_054534026.1	14.5	0.0324997655	0.0202450870	0.6229302476
Nomascus leucogenys	XM_030795839.1	17.6	0.0433596729	0.0176788864	0.4077264698
Macaca mulatta	XM_015126329.2	27.5	0.0562395100	0.0626232477	1.1135098397
Macaca fascicularis		27.5			
Papio anubis	XM_017953467.3	27.5	0.0502970028	0.0646190693	1.2847499003
Chlorocebus sabaeus	XM_007993565.2	27.5	0.0503486672	0.0597888053	1.1874952929
Rhinopithecus roxellana		27.5			
Saimiri boliviensis	XM_003936257.3	39.9	0.1110968531	0.0774444943	0.6970899010
Callithrix jacchus	XM_035281577.2	39.9	0.1391263603	0.0827521414	0.5947984349
Tarsius syrichta		49.6			
Otolemur garnettii		57.2			
Microcebus murinus		57.2			

**Fig 5B. Gene: *KCNG4***					
Potassium voltage-gated channel modifier subfamily G member 4. Located downstream CDH13					
	Accession #				
Compared to *H. sapiens*	NM_172347.3	MYr ago	**dS**	**dN**	**dN/dS**
Pan troglodyte	XM_523443.7	7.0	0.0242445363	0.0066972684	0.276238255
Pan paniscus	XM_003820866.4	7.0	0.0295970662	0.0079279835	0.267863831
Gorilla gorilla	XM_004058074.4	8.1	0.0200846947	0.0077185384	0.384299514
Pongo abelii					
Nomascus leucogenys	XM_015126715.2	17.6	0.0483231993	0.0151180023	0.31285185
Macaca mulatta		27.5	0.0821009635	0.0126474044	0.154046967
Macaca fascicularis	XM_045381907.1	27.5	0.0821071911	0.0144959642	0.176549264
Papio anubis	XM_021932225.2	27.5	0.0801961153	0.0138826645	0.17310894
Chlorocebus sabaeus	XM_007994219.2	27.5	0.0632858053	0.0116294597	0.183760949
Rhinopithecus roxellana	XM_010355635.2	27.5	0.0768769237	0.0134661244	0.175164714
Saimiri boliviensis	XM_010333659.2	39.9	0.1019304200	0.0234330327	0.229892437
Callithrix jacchus	XM_035281884.2	39.9	0.1030424444	0.0240371935	0.233274683
Tarsius syrichta					
Otolemur garnettii	XM_003791294.2	57.2	0.2391465349	0.0708800083	0.296387352
Microcebus murinus	XM_012738475.1	57.2	0.1791927730	0.0814963635	0.454797156

**Fig 5B. Gene: *PRLR***					
Prolactin receptor - Located downstream CDH6					
	Accession #				
Compared to *H. sapiens*	NM_000949.7_	MYr ago	**dS**	**dN**	**dN/dS**
Pan troglodyte	XM_001150064.7	7.0	0.0148266130	0.0000000000	0
Pan paniscus	XM_034960180.2	7.0	0.0114493601	0.0016920490	0.147785465
Gorilla gorilla	XM_004058975.4	8.1	0.0164572454	0.0052076515	0.316435185
Pongo abelii	XM_054555266.1	14.5	0.0337204385	0.0071001495	0.210559228
Nomascus leucogenys	XM_003274943.4	17.6	0.0353445894	0.0077757314	0.219997786
Macaca mulatta	XM_001092241.4	27.5	0.0599873553	0.0180298426	0.300560718
Macaca fascicularis	XM_005556706.3	27.5	0.0545710902	0.0173472089	0.317882763
Papio anubis					
Chlorocebus sabaeus	XM_007961343.2	27.5	0.0440113050	0.0173439756	0.394080012
Rhinopithecus roxellana	XM_010384082.2	27.5	0.0458663111	0.0144606235	0.315277666
Saimiri boliviensis	XM_003925930.3	39.9	0.0920537018	0.0525686063	0.571064555
Callithrix jacchus	XM_008992156.4	39.9	0.0917008312	0.0580977236	0.633557219
Tarsius syrichta	XM_008062674.2	49.6	0.2158771647	0.1337864964	0.61973436
Otolemur garnettii	XM_023514994.1	57.2	0.3460889611	0.1486205985	0.42942889
Microcebus murinus	XM_012758132.2	57.2	0.2462127505	0.1100119198	0.446816502

**Fig 5B. Gene: *TAS2R1***					
Taste 2 receptor member 1- Located upstream of CDH9, CDH12, CDH18					
	Accession #				
Compared to *H. sapiens*	NM_019599.3	MYr ago	**dS**	**dN**	**dN/dS**
Pan troglodyte	AB713189.1	7.0	0.010444559	0.01061505	1.016323447
Pan paniscus	XM_003809222.4	7.0	0.010414635	0.008702719	0.835623991
Gorilla gorilla	XM_055367630.1	8.1	0.017713835	0.006395256	0.361031685
Pongo abelii	XM_002815422.3	14.5	0.051411446	0.022499339	0.437632874
Nomascus leucogenys	XM_003263158.4	17.6	0.051642518	0.037167423	0.719705857
Macaca mulatta	XM_015139731.2	27.5	0.059330629	0.069105136	1.164746407
Macaca fascicularis	XM_005556591.3	27.5	0.066891656	0.06988511	1.044750785
Papio anubis	XM_003899492.5	27.5	0.067553758	0.064645555	0.956949784
Chlorocebus sabaeus	XM_007961196.2	27.5	0.063407845	0.064416834	1.015912686
Rhinopithecus roxellana	XM_010356696.1	27.5	0.063908292	0.071722844	1.122277593
Saimiri boliviensis	XM_003932500.3	39.9	0.14595642	0.133518948	0.9147864
Callithrix jacchus	XM_002745124.6	39.9	0.142115099	0.118145766	0.831338587
Tarsius syrichta	XM_008064818.1	49.6	0.382903283	0.253251641	0.661398458
Otolemur garnettii	XM_003789570.1	57.2	0.391650605	0.278767563	0.711776159
Microcebus murinus	XM_012791123.1	57.2	0.359379056	0.245112232	0.682043731

Fig 5A, B. Independent-Samples Mann-Whitney U Test

CDHs Type I	Exact Sig. (2-sided test)	Standardized Test Statistic
SLC7A6 vs SLC7A6	1.000[c]	0.000
SLC7A6 vs CDH1	.701[c]	0.391
SLC7A6 vs CDH3	.780[c]	-0.306
SNTB2 vs SNTB2	1.000[c]	0.000
SNTB2 vs CDH1	.004[c]	-2.791
SNTB2 vs CDH3	<.001[c]	-3.939
TMEM241 vs TMEM24	1.000[c]	0.000
TMEM241 vs CDH2	<.001[c]	4.491
HRH3 vs HRH3	1.000[c]	0.000
HRH3 vs CDH4	<.001[c]	3.904
SNTB2 vs SNTB2	1.000[c]	0.000
SNTB2 vs CDH2	.892[c]	0.161
SNTB2 vs CDH4	.217[c]	1.267

CDHs Type II	Exact Sig. (2-sided test)	Standardized Test Statistic
CD226 vs CD226	1.000[c]	0.000
CD226 vs CDH7	<.001[c]	4.491
CD226 vs CDH19	<.001[c]	4.491
CD226 vs CDH20	<.001[c]	4.491
CMTM1 vs CMTM1	1.000[c]	0.000
CMTM1 vs CDH5	<.001[c]	4.049
CMTM1 vs CDH8	<.001[c]	4.206
CMTM1 vs CDH11	<.001[c]	4.160
KCNG4 vs KCNG4	1.000[c]	0.000
KCNG4 vs CDH13	<.001[c]	4.400
PRLR vs PRLR	1.000[c]	0.000
PRLR vs CDH6	<.001[c]	3.797
TAS2R1 vs TAS2R1	1.000[c]	0.000
TAS2R1 vs CDH9	<.001[c]	4.668
TAS2R1 vs CDH10	<.001c	4.672
TAS2R1 vs CDH12	<.001[c]	4.667
TAS2R1 vs CDH18	<.001[c]	4.666

Fig 6A. Sperman's correlation analysis of classical cadherins dN and dN/dS values between *H. sapiens* and non-human primate vs. mRNA expression in 52 human tissues (GTEx)																													
		Pan t.		***Pan p.***		***Gorilla g. g.***		***Pongo a.***		***Nomascus l.***		***Macaca m.***		***Macaca f.***		***Papio a.***		***Rhinopithecus r.***		***Saimiri b. b.***		***Callithrix j.***		***Tarsius s.***		***Otolemur g.***		***Microcebus m.***	
**Tissue**	**Sample #**	**dN**	**dN/dS**	**dN**	**dN/dS**	**dN**	**dN/dS**	**dN**	**dN/dS**	**dN**	**dN/dS**	**dN**	**dN/dS**	**dN**	**dN/dS**	**dN**	**dN/dS**	**dN**	**dN/dS**	**dN**	**dN/dS**	**dN**	**dN/dS**	**dN**	**dN/dS**	**dN**	**dN/dS**	**dN**	**dN/dS**
Brain - Amygdala	1	0.070	0.078	-0.009	-0.082	-0.424	-0.335	-0.194	-0.157	-0.176	-0.276	-0.185	-0.158	-0.139	-0.135	-0.117	-0.104	-0.207	-0.201	-0.311	-0.212	-0.261	-0.257	-0.276	-0.329	-0.228	-0.147	-0.158	-0.174
Brain - Anterior cingulate cortex (BA24)	2	-0.116	-0.064	-0.189	-0.195	-.499*	-0.347	-0.372	-0.291	-0.346	-0.453	-0.282	-0.261	-0.249	-0.220	-0.228	-0.214	-0.313	-0.313	-0.430	-0.333	-0.364	-0.373	-0.353	-0.382	-0.314	-0.211	-0.292	-0.288
Brain - Caudate	3	0.278	0.276	0.146	0.065	-0.346	-0.287	-0.019	0.010	-0.053	-0.084	-0.098	-0.055	-0.061	-0.102	-0.073	-0.036	-0.065	-0.040	-0.170	-0.079	-0.183	-0.137	-0.262	-0.291	-0.086	-0.037	-0.077	-0.133
Brain - Cerebellar Hemisphere	4	0.205	0.270	0.156	0.133	-0.018	0.049	-0.141	-0.042	-0.009	-0.024	0.049	0.123	0.088	0.141	0.042	0.137	-0.112	0.007	-0.104	-0.003	-0.249	-0.164	-0.185	-0.265	-0.270	-0.221	-0.158	-0.121
Brain - Cerebellum	5	0.222	0.283	0.173	0.129	-0.006	0.022	-0.127	-0.024	-0.001	-0.015	0.079	0.152	0.115	0.164	0.067	0.150	-0.104	0.013	-0.057	0.036	-0.216	-0.137	-0.162	-0.232	-0.248	-0.199	-0.137	-0.102
Brain - Cortex	6	-0.055	-0.039	-0.135	-0.185	-0.440	-0.345	-0.285	-0.202	-0.245	-0.362	-0.203	-0.193	-0.164	-0.143	-0.139	-0.135	-0.259	-0.263	-0.344	-0.249	-0.300	-0.317	-0.282	-0.318	-0.252	-0.147	-0.191	-0.162
Brain - Frontal Cortex (BA9)	7	-0.224	-0.162	-0.336	-0.342	-.555*	-0.391	-0.426	-0.292	-0.430	-.511*	-0.360	-0.319	-0.325	-0.261	-0.309	-0.267	-0.379	-0.377	-.486*	-0.406	-0.443	-0.451	-0.394	-0.424	-0.390	-0.289	-0.356	-0.317
Brain - Hippocampus	8	0.101	0.120	-0.001	-0.098	-0.400	-0.312	-0.104	-0.065	-0.135	-0.228	-0.185	-0.131	-0.135	-0.123	-0.135	-0.127	-0.185	-0.187	-0.261	-0.181	-0.216	-0.172	-0.250	-0.324	-0.243	-0.176	-0.143	-0.170
Brain - Hypothalamus	9	0.016	0.057	-0.117	-0.185	-0.393	-0.237	-0.174	-0.094	-0.199	-0.323	-0.218	-0.170	-0.160	-0.127	-0.172	-0.154	-0.205	-0.222	-0.329	-0.263	-0.280	-0.243	-0.291	-0.362	-0.257	-0.169	-0.179	-0.183
Brain - Nucleus accumbens	10	0.053	0.114	-0.022	-0.034	-.510*	-0.389	-0.339	-0.262	-0.329	-0.360	-0.243	-0.222	-0.243	-0.267	-0.251	-0.259	-0.282	-0.298	-0.302	-0.251	-0.327	-0.344	-0.426	-0.412	-0.250	-0.225	-0.307	-0.348
Brain - Putamen	11	0.260	0.247	0.140	0.044	-0.338	-0.301	0.009	0.030	-0.051	-0.082	-0.117	-0.061	-0.071	-0.100	-0.077	-0.030	-0.067	-0.038	-0.185	-0.094	-0.189	-0.133	-0.256	-0.303	-0.098	-0.049	-0.069	-0.127
Brain - Spinal cord (cervical c-1)	12	0.255	0.358	0.100	0.090	-0.175	-0.026	0.057	0.187	-0.009	0.007	-0.057	0.100	-0.018	0.082	-0.057	0.079	-0.067	0.073	-0.152	0.026	-0.141	0.063	-0.229	-0.274	-0.199	-0.103	-0.139	-0.119
Brain - Substantia nigra	13	0.193	0.276	0.016	-0.018	-0.245	-0.093	0.009	0.110	-0.065	-0.075	-0.115	0.026	-0.063	0.026	-0.104	0.015	-0.088	0.018	-0.216	-0.061	-0.199	-0.024	-0.288	-0.341	-0.260	-0.162	-0.164	-0.158
Adipose - Subcutaneous	14	.527*	.543*	0.356	0.267	0.468	.509*	.549*	.482*	.497*	0.461	0.343	0.398	0.392	0.411	0.373	0.422	0.416	.499*	0.328	0.436	.470*	.628**	0.215	0.169	0.314	0.410	0.440	0.386
Adipose - Visceral (Omentum)	15	.526*	.505*	0.328	0.221	0.446	.482*	.554*	0.438	.551*	.506*	0.386	0.411	0.437	0.433	0.429	0.458	0.464	.535*	0.355	.473*	.497*	.626**	0.239	0.195	0.342	0.455	.487*	0.440
Adrenal Gland	16	.495*	.608**	0.377	0.280	0.408	.474*	0.382	0.415	0.352	0.257	0.284	0.364	0.319	0.370	0.259	0.255	0.185	0.232	0.370	0.393	0.437	.567*	0.253	0.185	0.130	0.174	0.286	0.207
Artery - Aorta	17	0.324	0.379	0.108	0.035	0.171	0.276	0.360	0.362	0.246	0.219	0.020	0.117	0.089	0.143	0.081	0.183	0.137	0.230	0.048	0.178	0.239	0.404	0.040	-0.033	0.067	0.181	0.223	0.172
Artery - Coronary	18	0.420	.474*	0.296	0.269	0.329	0.436	0.464	0.447	0.373	0.297	0.173	0.259	0.246	0.289	0.230	0.338	0.265	0.373	0.142	0.283	0.334	.510*	0.168	0.087	0.172	0.280	0.332	0.264
Artery - Tibial	19	0.427	0.434	0.192	0.091	0.304	0.399	.474*	0.411	0.412	0.357	0.145	0.197	0.215	0.219	0.199	0.255	0.261	0.338	0.178	0.279	0.342	.491*	0.144	0.063	0.196	0.297	0.345	0.268
Bladder	20	.562*	.547*	0.386	0.229	0.395	0.389	.617**	.522*	.554*	.548*	0.406	.478*	0.451	.474*	0.439	.478*	0.453	.525*	.472*	.562*	.556*	.695**	0.365	0.306	0.299	0.380	.507*	0.447
Breast - Mammary Tissue	21	.546*	.486*	.480*	0.300	.581*	0.440	.685**	.527*	.633**	.582*	.545*	.548*	.589*	.568*	.583*	.561*	.563*	.610**	.543*	.584*	.682**	.758**	0.427	0.378	0.392	.483*	.626**	.560*
Cervix - Ectocervix	22	.593**	.589*	0.406	0.278	.536*	.564*	.612**	.515*	.628**	.567*	0.414	0.441	.474*	.474*	0.457	.480*	0.445	.509*	0.441	.540*	.587*	.707**	0.374	0.303	0.358	0.456	.556*	.513*
Cervix - Endocervix	23	.516*	.554*	0.307	0.198	0.446	.529*	.520*	0.436	.521*	.472*	0.360	0.418	0.406	0.434	0.381	0.404	0.375	0.451	0.424	.519*	.544*	.686**	0.306	0.241	0.248	0.336	0.439	0.366
Colon - Sigmoid	24	.602**	.602**	0.398	0.278	0.346	0.387	.593**	.551*	.490*	.486*	0.288	0.391	0.348	0.399	0.337	0.439	0.387	0.451	0.395	.507*	0.457	.606**	0.326	0.268	0.338	0.402	.490*	0.434
Colon - Transverse	25	.591**	.535*	0.462	0.253	0.365	0.251	.663**	.562*	.546*	.540*	0.453	.531*	.503*	.536*	.511*	.558*	.490*	.544*	.552*	.624**	.598**	.690**	0.415	0.362	0.363	0.439	.598**	.529*
Esophagus - Gastroesophageal Junction	26	.552*	.579*	0.313	0.229	0.237	0.328	.504*	.516*	0.391	0.377	0.176	0.282	0.238	0.278	0.212	0.319	0.307	0.364	0.232	0.352	0.315	.482*	0.200	0.144	0.287	0.360	0.366	0.325
Esophagus - Mucosa	27	.628**	.549*	.563*	0.374	.624**	0.436	.703**	.531*	.710**	.704**	.655**	.657**	.694**	.675**	.700**	.700**	.644**	.719**	.595**	.667**	.702**	.760**	0.478	0.434	0.462	.558*	.669**	.640**
Esophagus - Muscularis	28	.543*	.564*	0.330	0.245	0.261	0.351	.517*	.505*	0.416	0.389	0.193	0.282	0.251	0.284	0.234	0.321	0.311	0.356	0.278	0.391	0.381	.529*	0.259	0.206	0.309	0.385	0.410	0.360
Fallopian Tube	29	.660**	.639**	.495*	0.342	.608**	.552*	.648**	.533*	.692**	.659**	.556*	.569*	.600**	.575*	.579*	.585*	.529*	.616**	.569*	.659**	.666**	.769**	0.421	0.365	0.414	.507*	.606**	.556*
Heart - Atrial Appendage	30	.490*	.526*	0.335	0.270	0.269	0.317	0.410	0.435	0.338	0.295	0.123	0.249	0.195	0.280	0.193	0.332	0.162	0.280	0.164	0.328	0.233	0.413	0.133	0.050	0.172	0.263	0.295	0.253
Heart - Left Ventricle	31	.541*	.568*	0.379	0.294	0.325	0.370	.531*	.552*	0.422	0.376	0.226	0.328	0.297	0.349	0.295	0.418	0.291	0.395	0.262	0.408	0.387	.552*	0.302	0.230	0.273	0.380	0.435	0.389
Kidney - Cortex	32	.560*	.502*	0.442	0.313	0.358	0.208	.582*	.504*	0.434	0.439	0.389	0.414	0.418	0.389	0.432	.488*	0.463	.515*	.509*	.585*	.569*	.616**	0.365	0.394	.490*	.581*	.593**	.490*
Kidney - Medulla	33	.583*	.506*	.479*	0.322	0.392	0.235	.588*	0.451	.515*	.503*	0.461	0.451	.488*	0.428	.505*	.509*	.507*	.536*	.548*	.610**	.622**	.641**	0.382	0.388	.498*	.581*	.624**	.525*
Liver	34	.653**	.612**	.579*	0.400	0.394	0.258	.723**	.675**	.527*	.518*	0.441	.535*	.501*	.520*	.493*	.588*	.501*	.577*	.518*	.575*	.567*	.671**	0.453	0.403	0.405	0.458	.610**	.514*
Lung	35	.490*	0.436	0.343	0.156	0.405	0.319	.604**	0.461	.545*	.510*	0.415	0.444	0.462	0.454	0.464	0.466	0.448	.512*	.479*	.549*	.588*	.679**	0.348	0.298	0.295	0.396	.539*	0.456
Minor Salivary Gland	36	.637**	.577*	.559*	0.336	.725**	.522*	.717**	.545*	.761**	.738**	.692**	.678**	.715**	.680**	.699**	.628**	.604**	.649**	.746**	.742**	.808**	.849**	.535*	0.491	0.458	.507*	.680**	.624**
Muscle - Skeletal	37	0.383	0.305	0.254	0.091	0.175	0.182	0.408	0.295	0.404	0.293	0.216	0.195	0.285	0.224	0.299	0.272	0.301	0.308	0.112	0.180	0.274	0.333	0.176	0.078	0.238	0.333	0.370	0.326
Nerve - Tibial	38	.554*	.529*	.508*	0.423	0.469	0.414	.627**	.544*	.531*	.534*	0.329	0.418	0.377	0.393	0.362	0.453	0.381	.484*	0.439	.517*	0.453	.612**	0.344	0.274	0.309	0.324	0.465	0.366
Ovary	39	.602**	.568*	0.427	0.208	0.349	0.331	.499*	0.348	.608**	.538*	0.414	0.391	0.467	0.375	0.441	0.377	0.354	0.387	0.449	.470*	.531*	.558*	0.294	0.176	0.238	0.279	0.463	0.364
Pancreas	40	.738**	.629**	.618**	0.327	0.448	0.231	.629**	.493*	.700**	.707**	.632**	.656**	.667**	.634**	.673**	.654**	.502*	.569*	.711**	.761**	.620**	.654**	0.489	0.412	0.384	0.428	.633**	.586*
Pituitary	41	0.322	0.397	0.247	0.078	0.122	0.018	0.282	0.377	0.179	0.181	0.422	.521*	0.441	.496*	0.381	0.426	0.247	0.356	0.408	0.443	0.362	0.441	0.224	0.203	-0.098	0.000	0.238	0.218
Prostate	42	.566*	.520*	.504*	0.338	0.451	0.310	.562*	0.423	.569*	.562*	.509*	.556*	.548*	.564*	.540*	.567*	0.453	.542*	.538*	.624**	.554*	.635**	0.335	0.274	0.257	0.333	.509*	0.455
Skin - Not Sun Exposed (Suprapubic)	43	.589*	.485*	.528*	0.280	.640**	0.433	.687**	.478*	.779**	.715**	.707**	.655**	.744**	.680**	.752**	.662**	.622**	.655**	.666**	.684**	.773**	.779**	.562*	0.485	0.451	.532*	.705**	.670**
Skin - Sun Exposed (Lower leg)	44	.612**	.516*	.531*	0.295	.668**	.470*	.732**	.544*	.787**	.732**	.723**	.692**	.761**	.715**	.765**	.699**	.666**	.703**	.699**	.728**	.796**	.827**	.582*	.518*	.500*	.586*	.738**	.705**
Small Intestine - Terminal Ileum	45	.585*	.522*	.489*	0.344	0.346	0.239	.644**	.527*	.505*	.513*	0.461	.517*	.494*	.498*	.513*	.569*	.560*	.598**	.527*	.616**	.593**	.672**	0.426	0.412	0.480	.556*	.614**	.534*
Spleen	46	.615**	.601**	.501*	0.333	0.394	0.375	.568*	0.444	.611**	.553*	.524*	.524*	.568*	.499*	.547*	.526*	.491*	.541*	.510*	.574*	.615**	.675**	0.431	0.339	0.322	0.388	.551*	.468*
Stomach	47	.652**	.640**	.535*	0.341	0.428	0.297	.678**	.636**	.533*	.532*	.496*	.596**	.547*	.584*	.527*	.606**	.489*	.592**	.565*	.639**	.599**	.720**	0.418	0.377	0.323	0.406	.573*	.491*
Testis	48	.477*	.579*	0.359	0.237	0.281	0.341	0.250	0.326	0.337	0.226	0.387	0.437	0.426	0.445	0.375	0.370	0.205	0.251	0.340	0.399	0.358	0.437	0.347	0.241	0.086	0.159	0.290	0.282
Thyroid	49	.594**	.528*	0.462	0.277	0.422	0.295	.661**	.534*	.574*	.599**	.478*	.532*	.523*	.521*	.527*	.579*	.535*	.622**	.506*	.600**	.575*	.679**	0.330	0.296	0.377	0.470	.576*	.512*
Uterus	50	.631**	.627**	0.421	0.288	.546*	.560*	.618**	.533*	.622**	.577*	0.425	0.451	.483*	.471*	0.461	.493*	0.463	.538*	0.449	.546*	.580*	.701**	0.347	0.291	0.381	0.482	.559*	.506*
Vagina	51	.560*	.500*	0.462	0.255	.527*	0.383	.681**	.541*	.639**	.593**	.550*	.579*	.606**	.614**	.612**	.622**	.548*	.614**	.556*	.631**	.678**	.754**	0.471	0.397	0.365	0.478	.647**	.600**
Whole Blood	52	.505*	0.406	.522*	0.276	0.369	0.226	.617**	0.431	.537*	0.417	.486*	0.451	.550*	.485*	.562*	.493*	.516*	.487*	0.428	0.407	.602**	.587*	0.465	0.361	0.342	0.411	.622**	.525*

2-tailed * $p < 0.05$, ** $p < 0.01$

Possitive correlation

Negative correlation

S4 Table. Spearman's correlation analysis of nucleotide substitutions versus gene expression in human tissues. Data plotted in Fig 6 A. Spearman's correlation coefficients between human and mouse evolutionary distance (dN/dS values) versus the expression level of cadherin orthologs in 52 human tissues samples from GTEx.

Fig 6A. Raw data. GTEx Human CDHs transcript expression																
**Gene**	Brain - Amygdala	Brain - Anterior cingulate cortex	Brain - Caudate (basal ganglia)	Brain - Cerebellar Hemisphere	Brain - Cerebellum	Brain - Cortex	Brain - Frontal Cortex (BA9)	Brain - Hippocampu s	Brain - Hypothalam us	Brain - Nucleus accumbens (basal	Brain - Putamen (basal ganglia)	Brain - Spinal cord (cervical c-1)	Brain - Substantia nigra	Adipose - Subcutaneo us	Adipose - Visceral (Omentum)	Adrenal Gland
CDH1	0.980677	0.308492	1.08387	0.211803	0.354075	0.723381	0.464353	0.974248	0.483642	0.624238	1.09492	0.672694	0.496714	0.145581	0.811697	0.0929811
CDH2	17.4951	19.9826	16.0667	20.6207	15.9982	19.4702	25.5783	13.4917	18.9592	16.9755	11.8383	11.1079	10.6992	0.17908	0.569949	66.0567
CDH3	1.00111	1.17954	1.09121	3.0678	3.01191	1.55092	1.41144	0.898285	1.02198	1.41373	0.955109	0.73538	0.648332	0.16139	3.11784	0.194819
CDH4	11.9898	10.4681	10.9782	1.62313	2.4464	11.9498	10.6675	5.6845	6.67941	14.3402	7.0884	1.99546	1.90334	0.101084	0.232662	0.709701
CDH5	4.58947	4.6212	6.64864	3.42945	4.66699	6.90993	5.48063	5.48609	6.25968	4.70807	6.89824	9.34145	8.51557	103.355	92.3825	12.7525
CDH6	1.5617	2.31986	4.74948	0.672177	0.758578	2.11164	2.74622	1.55794	2.78519	2.03927	3.91538	2.44039	2.47266	4.33139	3.85759	0.144475
CDH7	0.118552	0.360421	0.593557	6.2711	4.50796	0.458056	0.834361	0.0409174	0.855825	0.568695	0.588946	0.102318	0.111561	0.00211515	0	0.00517819
CDH8	1.81845	2.44206	3.89864	0.152214	0.202566	4.63086	6.31738	3.05325	3.29249	4.67678	3.08187	3.19791	2.74484	0.0483438	0.0182485	0.00707047
CDH9	2.1873	5.05615	2.20934	0	0	5.89061	6.59465	1.2223	1.5291	5.57437	1.53379	0.0269178	0.0975315	0	0	0
CDH10	7.61745	12.6483	4.47777	40.3293	34.7252	13.7382	20.7022	5.20391	6.9474	5.6649	3.20177	2.59164	4.05132	0.0176275	0.0209033	0.148187
CDH11	2.97759	3.40082	5.64862	2.87968	3.00335	3.08509	3.49961	3.45583	3.70584	5.12006	4.88184	6.74211	3.84506	8.08544	6.87398	2.9452
CDH12	0.189122	0.741212	0.593688	0.409188	0.396709	1.31546	1.73724	0.25986	0.913757	1.8809	0.288107	0.910449	0.232832	0.100445	0.0090602	2.16721
CDH13	4.14881	10.1687	0.984885	0.416492	0.3828	7.93104	13.5381	2.3464	5.08062	0.852356	0.974202	1.59553	1.68258	9.36522	7.50979	0.588215
CDH18	1.32265	2.98556	0.941836	24.7827	20.2385	5.80707	8.54152	1.11068	4.39485	1.38794	0.791042	0.520502	0.611312	0	0	0.0186026
CDH19	1.55834	0.568231	1.91288	0.293342	0.329523	0.835217	0.892115	2.32496	1.19671	1.0008	2.66589	4.51356	2.48571	1.68968	1.35822	1.28321
CDH20	16.2369	14.7586	17.4867	6.94827	10.6006	15.7064	14.6195	11.9203	11.3432	15.8229	14.4742	20.5644	15.3414	0.921779	0.841864	0.155149
CDH22	8.15901	11.8605	3.48933	78.6886	78.4857	21.1438	19.412	2.63795	4.40514	2.55509	2.79441	2.3866	0.879347	0.0555146	0.148251	0.080565
CDH24	4.67814	5.39149	6.373	25.1305	26.3144	6.53706	6.32922	3.94194	6.03063	5.83976	5.56281	2.59283	3.59031	10.0041	7.18472	5.71897

Fig 6A. Raw data. GTEx Human CDHs transcript expression																
**Gene**	Artery - Aorta	Artery - Coronary	Artery - Tibial	Bladder	Breast - Mammary Tissue	Cervix - Ectocervix	Cervix - Endocervix	Colon - Sigmoid	Colon - Transverse	Esophagus - Gastroesoph ageal Junction	Esophagus - Mucosa	Esophagus - Muscularis	Fallopian Tube	Heart - Atrial Appendage	Heart - Left Ventricle	Kidney - Cortex
CDH1	0.134844	0.199497	0.106238	6.34936	29.2679	0.942261	1.90353	0.810131	62.4285	0.185783	140.349	0.19265	4.8786	0.0899651	0.0526798	20.7794
CDH2	32.9052	41.47	3.92904	0.93729	0.377933	5.42222	5.09182	6.95878	1.91244	11.1916	0.291524	8.19019	1.65876	52.4394	67.453	4.43723
CDH3	0.11417	0.21554	0.125688	0.523067	6.65018	18.8873	10.4182	0.242481	0.195841	0.236099	24.4695	0.21043	26.3158	0.0838591	0.0228282	3.53841
CDH4	0.219234	0.211135	0.265331	0.517509	0.359062	0.794042	0.923524	0.646947	0.256786	0.737814	0.155982	0.948543	0.821846	0.149919	0.0689591	0.42558
CDH5	12.4898	29.8228	17.8233	22.8562	80.436	28.8484	29.246	19.3047	12.7754	27.4926	8.34066	29.109	30.8603	21.9792	27.5264	13.1865
CDH6	1.55747	6.22294	16.6516	2.50692	2.73052	5.25497	3.60922	1.73139	0.944408	3.06258	1.18737	1.58983	3.66415	0.699964	0.622222	7.93911
CDH7	0	0.0172076	0.00530625	0.0112243	0.00539715	0.0103926	0.0060908	0.0108519	0.00501081	0.00594597	0	0.00658902	0.00465828	0.00815146	0	0.00275715
CDH8	1.53264	0.0148774	0.0734184	0.10106	0.0487961	0.106545	0.0690836	0.104743	0.0311104	0.270103	0.0506122	0.287014	0.0490986	0.00993412	0.0126259	0.0099199
CDH9	0	0	0	0	0	0	0	0.0318155	0.0145464	0.0261207	0	0.0191814	0	0	0	0.863728
CDH10	0.0239416	0.0133887	0.0232078	0.0561895	0.0230666	0.0729694	0.337594	0.232959	0.144717	0.0717091	0.016093	0.0627298	0.0350794	0.112323	0	0.0720157
CDH11	24.9243	26.4645	16.9586	14.0072	6.5724	17.9252	30.1509	5.32058	3.97836	5.59309	1.54118	4.889	6.38894	2.29048	0.915314	1.62765
CDH12	0	0.00787875	0.00699804	0.0341137	0.168277	0.0373672	0.211741	0.00868358	0.0130446	0.0120868	0.0162641	0.00969634	0.164959	0	0	0.0515649
CDH13	131.264	55.437	55.2144	3.16879	7.29011	23.9838	21.8448	3.65521	1.81959	2.4088	3.18892	2.12854	5.56431	11.3764	12.0687	0.886156
CDH18	0	0	0	0.0168052	0	0.0113341	0.0146746	0.0336592	0.0176285	0.0392389	0	0.0189967	0.0264867	0	0	0.00657842
CDH19	1.87022	22.0965	2.58293	7.52164	1.62898	10.627	14.1641	28.1615	8.90083	19.1778	1.79723	19.0991	3.48629	27.7084	7.56032	0.250945
CDH20	0.2002	0.30928	0.223991	0.431851	0.578347	0.103649	0.263458	0.0716159	0.0714047	0.240507	0.317048	0.162067	0.7011	0.108226	0.0521451	0.0915201
CDH22	0.0315047	0.0604712	0.0240261	0.0396747	0.120097	0.241033	0.160759	0.0846415	0.0795319	0.0692262	1.06725	0.0624398	1.3864	0.824318	0.129163	0.155544
CDH24	4.94473	8.78484	5.2155	6.78262	9.11069	19.7485	18.2229	7.81071	6.5761	6.84525	9.35127	6.66006	12.4836	2.8496	1.75303	6.25316

Fig 6A. Raw data. GTEx Human CDHs transcript expression

Gene	Kidney - Medulla	Liver	Lung	Minor Salivary Gland	Muscle - Skeletal	Nerve - Tibial	Ovary	Pancreas	Pituitary	Prostate	Skin - Not Sun Exposed (Suprapubic)	Skin - Sun Exposed (Lower leg)	Small Intestine - Terminal Ileum	Spleen	Stomach	Testis
CDH1	35.5463	24.5229	31.9114	87.1158	0.0722464	15.5049	2.84119	53.4698	24.2401	57.7253	108.785	107.784	59.5937	8.67377	61.0324	0.753834
CDH2	3.41153	13.9009	0.808859	0.371817	0.16768	7.27717	6.25963	0.19292	11.1835	2.55818	0.249869	0.223213	1.0645	7.93956	6.28866	37.3268
CDH3	6.42587	0.0309817	2.18736	16.2289	0.0300018	1.53549	50.1519	0.327592	5.73174	16.4474	20.8305	17.0498	0.529892	10.6143	0.771604	2.26217
CDH4	0.545913	0.137568	0.501317	0.542717	0.675364	1.58011	12.0679	0.389101	0.823849	0.623035	1.02024	0.830854	0.321291	6.15915	0.358632	2.44739
CDH5	15.052	3.96184	128.521	11.9971	14.1595	51.3909	8.87297	3.34521	8.66126	14.0435	12.4653	19.6788	17.5706	18.8548	10.7743	14.4617
CDH6	5.69964	0.204209	5.50359	0.800784	0.576418	10.4396	0.683461	0.227598	0.983167	1.76839	0.681693	1.05235	1.2824	0.490024	0.927886	0.234216
CDH7	0	0	0	0.00766655	0	3.36217	0.0166121	0	0.275593	0.13217	0.0104342	0.00773286	0.00768058	0.0044296	0	0.251708
CDH8	0.0279481	0	0.0453705	0.056658	0.050089	0.0381472	0.306457	0.00418473	0.354041	0.174811	0.0732234	0.094155	0.0346336	0.0067936	0.009914	0.159752
CDH9	0.2954	0	0	0	0	0	0	0	0	0	0	0	0.121971	0	0	0.0998366
CDH10	0.0346906	0	0.164498	0.0280617	0	0.102163	0.0342466	0.127733	1.74797	3.22243	0.0275661	0.0618318	0.121149	0	0.153172	0.763331
CDH11	3.95273	0.182571	27.1929	6.05358	0.449809	20.1057	74.7302	0.504071	5.93315	8.89154	4.26931	5.89705	3.19047	22.7162	2.92167	3.07269
CDH12	0.0211811	0	0.013748	1.40839	0	0.0509849	0.0168514	0.0104787	2.04892	0.0238913	0.436523	0.330855	0	0	0.0290594	0.910701
CDH13	3.13412	0.125581	13.0064	3.07379	6.78559	2.80732	1.53719	0.0622211	0.420408	2.53414	12.1033	14.5437	1.0613	1.15039	0.470062	1.77645
CDH18	0.0132768	0	0	0.0129098	0	0.03365	0.0190988	0.0435609	6.7725	0.0545768	0.0552719	0.114002	0.0142482	0.00913285	0.0165667	4.93506
CDH19	0.288808	0.906758	1.98687	4.80676	0.15098	161.296	0.902778	0.46633	0.194333	5.12358	2.65087	2.59872	3.43294	1.0058	2.56141	0.894918
CDH20	0.0985636	0.0249404	0.176045	0.218194	1.59971	0.628414	0.138077	0.0303202	2.35317	0.258201	0.633548	0.616981	0.258804	0.116499	0.20329	1.29769
CDH22	0.244952	0.0124001	0.123186	0.399514	0.300692	0.230738	0.468723	0.982793	1.72825	1.59839	2.98694	1.37942	0.112136	0.0185768	0.17028	2.94771
CDH24	9.25669	0.576602	10.381	7.40566	0.719452	9.91564	7.11393	2.35857	4.82134	10.9029	6.80148	6.50598	6.34413	9.05076	4.2435	6.58375

Fig 6A. Raw data. GTEx Human CDHs transcript expression

Gene	Thyroid	Uterus	Vagina	Whole Blood
CDH1	130.134	0.583424	90.655	0.214911
CDH2	1.11457	1.91159	4.66257	0.0723074
CDH3	1.62623	18.5564	12.5301	0.0241779
CDH4	0.430516	0.424944	0.442318	0.0744885
CDH5	59.8335	35.7299	17.6066	0.116705
CDH6	3.24556	3.16654	2.30492	0.0147642
CDH7	0	0	0.00126455	0
CDH8	0.0548694	0.0384419	0.0735295	0
CDH9	0	0	0	0
CDH10	0.0259846	0.0271043	0.132801	0
CDH11	5.86723	18.0715	10.265	0.0184156
CDH12	0	0.0312891	0.0144938	0
CDH13	1.77604	28.5168	16.9112	0.102544
CDH18	0	0.00252682	0.028811	0
CDH19	1.83034	1.33327	5.81013	0.0130628
CDH20	0.669607	0.121625	0.150242	0.027324
CDH22	0.350686	0.141014	0.905166	0.0124056
CDH24	16.9501	28.517	13.7516	0.368511

Fig 6A. Raw data.

Gene	*Pan troglodyte*	*Pan paniscus*	*Gorilla gorilla*	*Pongo abelii*	*Nomascus leucogenys*	*Macaca mulatta*	*Macaca fascicularis*	*Papio anubis*	*Chlorocebus sabaeus*	*Rhinopithecus roxellana*	*Saimiri boliviensis*	*Callithrix jacchus*	*Tarsius syrichta*	*Otolemur garnettii*	*Microcebus murinus*	*Mus musculus*
	**dN**	**dN**	**dN**	**dN**	**dN**	**dN**	**dN**	**dN**	**dN**	**dN**	**dN**	**dN**	**dN**	**dN**	**dN**	**dN**
CDH1	0.00128176	0.00185367	0	0.00815444	0.0061314	0.01793392	0.01793982	0.0183111	0.01495157	0.01248539	0.04270018	0.03365719	0.05053467		0.06903323	0.08955178
CDH2	0.00129324	0.00129324	0.0006462	0.00300247	0.00064683	0.00182259	0.00182259	0.00182259	0.00182273	0.00182259	0.00299918	0.00352774	0.00760441	0.00512497	0.00583958	0.00711759
CDH3	0.00193537	0.0040134	0.01440368	0.0051304	0.01692908	0.01807661	0.01822618	0.01723053	0.01837403	0.01595123	0.02787817	0.02808764	0.04190465	0.04362395	0.04054623	0.06906127
CDH4	0.00129786	0.00129786	0.0006483	0.00379003	0.00772298	0.00259825	0.00325099	0.00325204	0.00277972	0.00313721	0.00618015	0.00683585	0.01504216	0.01606882	0.01861368	0.026476
CDH5	0.00133112	0.00186849	0.0030727	0.00616668	0.00812306	0.0061686	0.00616918	0.00684164	0.00495612	0.00562599	0.04126458	0.03177331	0.0901109	0.07685255	0.07227345	0.14198955
CDH6	0.00133556	0.00066756	0.00186971	0.00521825	0.00374394	0.0018694	0.0018694	0.00186867	0.00307455	0.00374361	0.00576042	0.00307416	0.00455762	0.02044913	0.01130829	0.01081731
CDH7	0	0.00119507	0.00132758	0.00066335	0.00132714	0.00132846	0.00132846	0.00132846	0.00132846	0.00132802	0.00252528	0.00199203	0.00478953	0.00546732	0.00372415	0.01008863
CDH8	0	0	0	0.00066313	0.00119552	0	0	0	0	0.00066357	0.00066335	0.00132846	0.00199402	0.00199146	0.00298983	0.00696671
CDH9	0	0.00066181	0	0	0.00052394	0.00209698	0.00157275	0.00209753	0.00289867	0.0030399	0.00556661	0.00490008	0.00935932	0.03955587	0.00849096	0.029965
CDH10	0	0	0	0	0.00053406	0.00119823	0.00119823	0.0005333	0.0005333	0.00053373	0.00280193	0.0005333	0.00132935	0.00133303	0.00136334	0.00728321
CDH11	0.00118506	0.00052851	0.00052851	0.00052851	0.00171416	0.00249982	0.00249982	0.00249982	0.00249954	0.00249889	0.00594317	0.00594208	0.00253688	0.00422406	0.00306907	0.0099742
CDH12	0.00066291	0.00085985	0.00309001	0.00265693	0.00199137	0.00305085	0.00305085	0.002521	0.00318717	0.00252062	0.00678598	0.00878752	0.00963924	0.00864975	0.00598135	0.01830551
CDH13	0	0	0.002922	0.00354318	0.00354834	0.00215256	0.00292005	0.00277342	0.00215256	0.0036903	0.00406634	0.01020371	0.01162793	0.0155122	0.015475	0.02233581
CDH18	0.00120413	0.00053656	0.00120462	0.00321469	0.00321558	0.00563821	0.00563778	0.00563821	0.00563778	0.00631016	0.00631691	0.00631392	0.01525198	0.01565619	0.01338835	0.01868455
CDH19	0.00385922	0.00438781	0.00424256	0.01280547	0.01324235	0.01531816	0.01478461	0.01478132	0.01632016	0.01682055	0.03252465	0.02535125	0.06003488	0.09036372	0.05542422	0.13368967
CDH20	0.00052963	0.0011879	0	0.00171756	0.00131579	0.00382737	0.00382737	0.00329708	0.00316621	0.00435949	0.00237724	0.00225003	0.00432467	0.00814734	0.00582318	0.01088022
CDH22	0.00130463	0.00130463	0.00129786	0.00261353	0.00455732	0.00407968	0.00407968	0.00594206	0.00289599	0.00289641	0.00604287	0.00366627		0.01043593	0.00859766	0.01965897
CDH24	0.00622092	0.00496416	0.00439664	0.00427545	0.00688322	0.00886384	0.00886384	0.0081716	0.00747977	0.00955499	0.0125912	0.01360965		0.01943025	0.01975841	0.03435224
	**dN/dS**	**dN/dS**	**dN/dS**	**dN/dS**	**dN/dS**	**dN/dS**	**dN/dS**	**dN/dS**	**dN/dS**	**dN/dS**	**dN/dS**	**dN/dS**	**dN/dS**	**dN/dS**	**dN/dS**	**dN/dS**
CDH1	0.04778435	0.06005928	0	0.12593994	0.16231027	0.21793395	0.21818758	0.23897769	0.20080232	0.15803713	0.27395701	0.2145301	0.16507007		0.21282896	0.14311364
CDH2	0.1052657	0.10530114	0.05130344	0.14011237	0.02334438	0.04695303	0.04999448	0.04654641	0.04185573	0.05142265	0.03639411	0.05490326	0.03868786	0.02683788	0.02460543	0.01349688
CDH3	0.13461484	0.28366321	1.10451455	0.10596262	0.29837393	0.21082953	0.20895365	0.17622852	0.20841695	0.18265871	0.21352963	0.20817102	0.14499009	0.15329753	0.12145386	0.12757646
CDH4	0.06375883	0.05622141	0.02204456	0.05393847	0.12149342	0.02785045	0.03569548	0.03020459	0.02685661	0.02967078	0.03241411	0.03769884	0.03899492	0.04474834	0.05098437	0.04221051
CDH5	0.09612994	0.12335845	0.16398655	0.14014499	0.12766785	0.0654092	0.06543806	0.06825979	0.05696297	0.06336335	0.34637112	0.25491696	0.34125845	0.25445876	0.25339683	0.31318863
CDH6	0.10403369	0.05783496	0.09737855	0.21413158	0.16240596	0.04226667	0.04510607	0.04793754	0.0763427	0.09884951	0.04763341	0.04276758	0.02534636	0.07343959	0.04043873	0.02872784
CDH7	0	0.11687988	0.08082276	0.01885833	0.03119265	0.01714438	0.01633067	0.01714232	0.01625646	0.01908005	0.01931488	0.01646677	0.02221152	0.01044584	0.01418579	0.02078627
CDH8	0	0	0	0.0272324	0.03455472	0	0	0	0	0.01165942	0.00726951	0.01327358	0.0103501	0.00872802	0.01596995	0.02094874
CDH9	0	0.07225532	0	0	0.01410355	0.02736964	0.02018646	0.03228552	0.04456919	0.04498953	0.04749214	0.03741985	0.04804693	0.11754895	0.03568336	0.05837858
CDH10	0	0	0	0	0.01446865	0.02057674	0.02057674	0.00785022	0.00806066	0.00895932	0.0303401	0.00520575	0.00665023	0.00384521	0.00602723	0.01477175
CDH11	0.086203	0.04749803	0.0597712	0.01530469	0.06076351	0.05325841	0.04889675	0.04408962	0.047212	0.05197026	0.05722921	0.06488	0.01180614	0.01590425	0.01227629	0.02521983
CDH12	0.0654164	0.05819913	0.17147068	0.08358344	0.05949266	0.0536793	0.0536793	0.04234212	0.04894961	0.04542446	0.05391056	0.07220449	0.04391939	0.02913604	0.0296518	0.04583632
CDH13	0	0	0.1740599	0.07922168	0.05160029	0.03417899	0.05167619	0.04521256	0.03439515	0.05223608	0.03475071	0.09160804	0.0417431	0.06253762	0.05980692	0.05012203
CDH18	0.07705766	0.0343367	0.07923055	0.11083478	0.08093661	0.08325348	0.08843025	0.07983348	0.08238647	0.0931245	0.05467018	0.05683148	0.08219859	0.05513297	0.07782444	0.04164073
CDH19	0.42022298	0.67074041	0.35679768	0.56580898	0.62218454	0.28620152	0.26241488	0.2756546	0.25250937	0.2715347	0.27721496	0.23697111	0.25464609	0.24789367	0.24599784	0.2391169
CDH20	0.03208327	0.06666011	0	0.04779578	0.02710841	0.05775304	0.05901897	0.0503116	0.04769409	0.06843401	0.02555192	0.02018217	0.01928073	0.03468771	0.0274269	0.02621629
CDH22	0.09065961	0.09954998	0.03344946	0.05975284	0.1407072	0.06458081	0.06467228	0.08738629	0.04499594	0.05284564	0.06468041	0.03823827		0.04543266	0.05611776	0.05003382
CDH24	0.5046324	0.28346734	0.17435695	0.09278983	0.23138604	0.14723182	0.14391881	0.12676251	0.11742674	0.15461522	0.12472878	0.13702712		0.06979946	0.08763669	0.08919972

S5 File. Fig 6B. Plotted data. Spearman's correlation coefficients of cadherins dN and dN/dS values between *H. sapiens* and non-humna primates vs. mRNA expression levels in 127 cell types from human cerebral cortex (scRNA seq, Allen Institute).

			Pan troglodyte		*Pan paniscus*		*Gorilla gorilla*		*Pongo abelii*		*Nomascus leucogenys*		*Macaca mulatta*		*Macaca fascicularis*		*Papio anubis*		*Chlorocebus sabaeus*		*Rhinopithecus roxellana*		*Saimiri boliviensis*		*Callithrix jacchus*		*Tarsius syrichta*		*Otolemur garnettii*		*Microcebus murinus*	
**Cell type**	**Cell type**	**Sample #**	**dN**	**dN/dS**	**dN**	**dN/dS**	**dN**	**dN/dS**	**dN**	**dN/dS**	**dN**	**dN/dS**	**dN**	**dN/dS**	**dN**	**dN/dS**	**dN**	**dN/dS**	**dN**	**dN/dS**	**dN**	**dN/dS**	**dN**	**dN/dS**	**dN**	**dN/dS**	**dN**	**dN/dS**	**dN**	**dN/dS**	**dN**	**dN/dS**
Lamp5_Lhx6_1		1	-0.398	-0.377	-0.375	-0.377	-0.429	-0.419	-0.272	-0.184	-0.369	-.506*	-0.261	-0.297	-0.233	-0.228	-0.211	-0.257	-0.321	-0.411	-0.189	-0.288	-0.455	-.508*	-0.235	-0.325	-0.105	-0.146	-0.212	-0.162	-0.202	-0.169
Lamp5_1	GABAergic	2	-0.226	-0.420	-0.302	-0.420	-0.379	-0.342	-0.074	-0.014	-0.192	-0.313	-0.176	-0.186	-0.111	-0.130	-0.141	-0.182	-0.236	-0.295	-0.053	-0.166	-0.360	-.478*	-0.204	-0.270	-0.051	-0.169	-0.224	-0.221	-0.119	-0.106
Lamp5_2	GABAergic	3	-0.358	-.511*	-0.447	-.511*	-0.348	-0.270	-0.116	-0.041	-0.253	-0.350	-0.257	-0.250	-0.194	-0.165	-0.210	-0.216	-0.320	-0.352	-0.102	-0.181	-0.449	-.513*	-0.219	-0.243	-0.122	-0.213	-0.287	-0.232	-0.168	-0.116
Lamp5_3	GABAergic	4	-.489*	-.555*	-.576*	-.555*	-.537*	-0.422	-0.330	-0.259	-0.426	-.500*	-0.361	-0.374	-0.339	-0.329	-0.324	-0.349	-0.409	-0.435	-0.213	-0.299	-.527*	-.540*	-0.273	-0.319	-0.247	-0.292	-0.318	-0.252	-0.297	-0.258
Lamp5_4	GABAergic	5	-0.375	-0.182	-0.392	-0.182	-.720**	-.665**	-.639**	-0.434	-.625**	-.546*	-0.277	-0.224	-0.330	-0.277	-0.312	-0.208	-0.268	-0.268	-0.303	-0.244	-.515*	-0.336	-.493*	-.491*	-0.459	-0.350	-0.360	-0.269	-.535*	-0.429
Lamp5_5	GABAergic	6	-0.451	-0.294	-.488*	-0.294	-.809**	-.734**	-.690**	-0.462	-.735**	-.657**	-0.346	-0.256	-0.400	-0.306	-0.376	-0.261	-0.325	-0.307	-0.361	-0.310	-.526*	-0.357	-.545*	-.527*	-0.476	-0.369	-0.422	-0.334	-.586*	-.498*
Lamp5_6	GABAergic	7	-.572*	-0.380	-.589*	-0.380	-.758**	-.662**	-.750**	-.542*	-.821**	-.760**	-0.436	-0.351	-.510*	-0.397	-.504*	-0.449	-0.348	-0.340	-.476*	-0.455	-.502*	-0.408	-.521*	-.505*	-.594*	-0.468	-.510*	-0.454	-.683**	-.641**
Pax6_1	GABAergic	8	-0.330	-.596**	-0.436	-.596**	-0.238	-0.255	-0.297	-0.235	-0.275	-0.375	-0.269	-0.275	-0.235	-0.195	-0.269	-0.388	-0.360	-0.426	-0.367	-0.433	-0.292	-0.452	-0.224	-0.288	-0.221	-0.316	-0.479	-.486*	-0.329	-0.320
Pax6_2	GABAergic	9	-0.302	-0.400	-0.314	-0.400	-.630**	-.709**	-0.468	-0.377	-.476*	-.523*	-0.332	-0.347	-0.328	-0.336	-0.343	-0.423	-0.336	-0.451	-0.376	-.498*	-0.403	-.529*	-.483*	-.616**	-0.342	-0.385	-0.406	-0.460	-0.423	-0.398
Pax6_3	GABAergic	10	-0.306	-.567*	-.535*	-.567*	-0.344	-0.211	-0.312	-0.257	-0.312	-0.328	-0.344	-0.332	-0.321	-0.287	-0.332	-0.348	-0.411	-0.393	-0.273	-0.327	-0.320	-0.340	-0.168	-0.197	-0.233	-0.286	-0.358	-0.310	-0.262	-0.253
Pax6_4	GABAergic	11	-0.439	-.755**	-.668**	-.755**	-0.477	-0.354	-0.421	-0.342	-0.409	-0.413	-0.393	-0.350	-0.373	-0.311	-0.407	-0.454	-.472*	-0.446	-0.397	-0.418	-0.427	-.481*	-0.328	-0.322	-0.439	-.519*	-.620**	-.582*	-.479*	-0.449
Sncg_3	GABAergic	12	-.624**	-.551*	-.696**	-.551*	-.567*	-0.467	-.637**	-0.452	-.681**	-.694**	-.590**	-.560*	-.620**	-.541*	-.594**	-.579*	-.599**	-.599**	-.601**	-.630**	-.479*	-0.460	-0.386	-0.418	-0.340	-0.290	-0.455	-0.413	-.518*	-.497*
Sncg_6	GABAergic	13	-.684**	-.623**	-.685**	-.623**	-.619**	-.568*	-.671**	-.644**	-.618**	-.679**	-.579*	-.667**	-.628**	-.677**	-.609**	-.784**	-.552*	-.637**	-.596**	-.756**	-0.467	-.605**	-0.390	-.537*	-0.415	-0.406	-0.435	-.499*	-.556*	-.586*
Sncg_2	GABAergic	14	-.501*	-0.342	-0.444	-0.342	-0.441	-0.446	-.602**	-.500*	-.573*	-.611**	-.516*	-.559*	-.568*	-.585*	-.590*	-.702**	-0.430	-.514*	-.614**	-.728**	-0.320	-.470*	-0.380	-.499*	-0.350	-0.311	-0.401	-.505*	-.531*	-.577*
Sncg_1	GABAergic	15	-0.409	-0.335	-0.430	-0.335	-.491*	-0.454	-.560*	-0.390	-.610**	-.628**	-0.321	-0.286	-0.382	-0.322	-0.399	-0.443	-0.230	-0.273	-0.425	-.480*	-0.225	-0.286	-0.291	-0.344	-0.313	-0.229	-0.348	-0.372	-.474*	-.505*
Sncg_8	GABAergic	16	-.655**	-0.432	-.558*	-0.432	-0.452	-0.434	-.629**	-.521*	-.673**	-.743**	-.653**	-.680**	-.691**	-.666**	-.691**	-.784**	-.584*	-.654**	-.704**	-.816**	-0.442	-.583*	-0.424	-.520*	-0.379	-0.350	-0.470	-.551*	-.567*	-.619**
Sncg_4	GABAergic	17	-.667**	-0.380	-.508*	-0.380	-.632**	-.653**	-.715**	-.635**	-.717**	-.759**	-.625**	-.686**	-.681**	-.714**	-.678**	-.804**	-.537*	-.643**	-.674**	-.809**	-.541*	-.684**	-.555*	-.688**	-.500*	-0.461	-.488*	-.592*	-.659**	-.684**
Sncg_5	GABAergic	18	-.576*	-0.392	-.478*	-0.392	-.484*	-.504*	-.627**	-.554*	-.601**	-.636**	-.596**	-.658**	-.649**	-.687**	-.654**	-.793**	-.517*	-.607**	-.676**	-.810**	-0.383	-.555*	-0.433	-.566*	-0.381	-0.356	-0.427	-.551*	-.563*	-.610**
Vip_14	GABAergic	19	-.625**	-.615**	-.646**	-.615**	-.572*	-.523*	-.475*	-0.420	-.583*	-.709**	-.480*	-.513*	-.475*	-0.429	-0.451	-.533*	-0.455	-.555*	-0.374	-.541*	-.495*	-.594**	-0.365	-.484*	-0.292	-0.295	-0.325	-0.307	-0.369	-0.361
Vip_15	GABAergic	20	-.587*	-.525*	-.604**	-.525*	-.655**	-.599*	-.518*	-0.351	-.696**	-.766**	-.499*	-0.455	-.497*	-0.396	-.497*	-.482*	-0.449	-.510*	-0.416	-.510*	-.555*	-.596**	-.473*	-.526*	-0.387	-0.359	-0.436	-0.400	-.475*	-0.442
Vip_16	GABAergic	21	-.709**	-.630**	-.743**	-.630**	-.533*	-0.440	-.632**	-.547*	-.608**	-.689**	-.579*	-.614**	-.587*	-.554*	-.575*	-.633**	-.606**	-.656**	-.554*	-.652**	-.554*	-.610**	-0.439	-.527*	-0.359	-0.375	-.483*	-0.476	-.520*	-.497*
Vip_4	GABAergic	22	-.671**	-.674**	-.737**	-.674**	-.802**	-.721**	-.642**	-.505*	-.734**	-.759**	-.571*	-.537*	-.579*	-.503*	-.579*	-.592**	-.558*	-.595**	-.511*	-.578*	-.672**	-.688**	-.577*	-.617**	-.596*	-.587*	-.612**	-.569*	-.637**	-.583*
Vip_5	GABAergic	23	-.803**	-.674**	-.841**	-.674**	-.780**	-.657**	-.784**	-.658**	-.820**	-.822**	-.654**	-.645**	-.701**	-.635**	-.675**	-.705**	-.618**	-.639**	-.611**	-.681**	-.652**	-.643**	-.550*	-.599**	-.603*	-.540*	-.580*	-.544*	-.692**	-.654**
Vip_6	GABAergic	24	-.804**	-.693**	-.849**	-.693**	-.732**	-.609**	-.706**	-.624**	-.754**	-.783**	-.615**	-.631**	-.642**	-.603**	-.620**	-.649**	-.555*	-.585*	-.487*	-.584*	-.680**	-.702**	-.578*	-.639**	-.548*	-.519*	-.484*	-0.462	-.624**	-.595**
Vip_9	GABAergic	25	-.760**	-.576*	-.711**	-.576*	-.638**	-.517*	-.638**	-.589*	-.664**	-.762**	-.482*	-.507*	-.497*	-0.462	-.479*	-.539*	-.486*	-.533*	-0.403	-.507*	-.633**	-.671**	-.492*	-.558*	-0.439	-0.460	-.482*	-0.461	-.556*	-.541*
Vip_2	GABAergic	26	-.495*	-.603**	-.548*	-.603**	-0.406	-0.350	-0.299	-0.211	-0.435	-.568*	-0.389	-0.353	-0.336	-0.242	-0.364	-0.397	-0.431	-.477*	-0.333	-0.401	-.498*	-.597**	-0.350	-0.370	-0.317	-0.397	-.499*	-0.456	-0.375	-0.360
Vip_11	GABAergic	27	-.498*	-.512*	-.503*	-.512*	-0.262	-0.184	-0.341	-0.268	-0.418	-.572*	-0.420	-0.389	-0.380	-0.299	-0.416	-.469*	-.477*	-.493*	-0.449	-.488*	-0.411	-.530*	-0.292	-0.297	-0.282	-0.381	-.537*	-.536*	-0.411	-.469*
Vip_13	GABAergic	28	-.485*	-0.458	-0.439	-0.458	-0.166	-0.128	-0.266	-0.206	-0.367	-.539*	-0.400	-0.391	-0.355	-0.280	-0.389	-0.455	-0.431	-.482*	-0.407	-.484*	-0.374	-.537*	-0.265	-0.297	-0.195	-0.289	-0.449	-0.470	-0.330	-0.377
Vip_1	GABAergic	29	-.681**	-.594**	-.697**	-.594**	-0.434	-0.342	-.537*	-0.427	-.634**	-.730**	-.630**	-.624**	-.633**	-.552*	-.622**	-.663**	-.614**	-.648**	-.592**	-.687**	-.481*	-.564*	-0.355	-0.410	-0.316	-0.317	-0.457	-0.452	-.470*	-.496*
Vip_12	GABAergic	30	-.650**	-.598**	-.664**	-.598**	-0.450	-0.386	-.472*	-0.355	-.603**	-.693**	-.641**	-.627**	-.623**	-.537*	-.621**	-.641**	-.623**	-.669**	-.566*	-.671**	-.519*	-.619**	-0.416	-.473*	-0.322	-0.344	-0.468	-0.470	-0.444	-0.442
Vip_18	GABAergic	31	-.721**	-.507*	-.651**	-.507*	-0.331	-0.273	-.510*	-0.415	-.616**	-.716**	-.581*	-.607**	-.599**	-.524*	-.577*	-.634**	-.517*	-.590**	-.521*	-.652**	-0.449	-.555*	-0.325	-0.407	-0.239	-0.203	-0.336	-0.345	-0.407	-0.407
Vip_19	GABAergic	32	-.725**	-.579*	-.723**	-.579*	-.570*	-.486*	-.646**	-.486*	-.755**	-.790**	-.748**	-.711**	-.760**	-.662**	-.756**	-.749**	-.714**	-.728**	-.721**	-.777**	-.597**	-.644**	-.519*	-.546*	-0.494	-0.471	-.597*	-.597*	-.623**	-.623**
Vip_21	GABAergic	33	-0.433	-.608**	-.623**	-.608**	-.671**	-.617**	-.526*	-0.311	-.625**	-.554*	-0.446	-0.354	-0.458	-0.383	-.518*	-.473*	-0.348	-0.336	-0.432	-0.421	-.499*	-.540*	-.583*	-.559*	-.589*	-.568*	-.582*	-.582*	-.639**	-.597**
Vip_23	GABAergic	34	-.545*	-0.414	-.571*	-0.414	-.732**	-.657**	-.657**	-0.410	-.785**	-.731**	-0.411	-0.301	-0.455	-0.319	-.477*	-0.394	-0.325	-0.326	-0.426	-0.406	-.540*	-.480*	-.566*	-.535*	-.559*	-0.471	-.557*	-.504*	-.663**	-.587*
Sst.Chodl_1	GABAergic	35	-.527*	-.552*	-.539*	-.552*	-.518*	-0.455	-.578*	-.529*	-.520*	-.511*	-.491*	-.489*	-.524*	-.526*	-.547*	-.647**	-.503*	-.504*	-.620**	-.671**	-0.295	-0.430	-0.408	-.481*	-0.445	-0.494	-.618**	-.712**	-.581*	-.638**
Sst_1	GABAergic	36	-.487*	-0.323	-.493*	-0.323	-0.215	-0.134	-0.349	-0.164	-.540*	-.599**	-0.337	-0.291	-0.365	-0.226	-0.345	-0.306	-0.243	-0.274	-0.284	-0.343	-0.235	-0.241	-0.108	-0.123	-0.063	0.037	-0.135	-0.075	-0.220	-0.207
Sst_4	GABAergic	37	-.667**	-.568*	-.702**	-.568*	-.491*	-0.375	-.562*	-.474*	-.611**	-.692**	-.624**	-.647**	-.635**	-.594**	-.616**	-.662**	-.620**	-.652**	-.562*	-.658**	-.516*	-.562*	-0.337	-0.407	-0.349	-0.340	-0.421	-0.401	-0.465	-.482*
Sst_5	GABAergic	38	-.698**	-.592**	-.703**	-.592**	-.507*	-0.418	-.566*	-.480*	-.649**	-.736**	-.684**	-.704**	-.693**	-.647**	-.665**	-.726**	-.675**	-.715**	-.626**	-.732**	-.537*	-.607**	-0.364	-0.438	-0.364	-0.356	-0.444	-0.437	-.485*	-.513*
Sst_7	GABAergic	39	-.643**	-.518*	-.618**	-.518*	-0.337	-0.275	-0.427	-0.281	-.583*	-.667**	-.614**	-.607**	-.607**	-.526*	-.605**	-.627**	-.577*	-.625**	-.550*	-.643**	-.491*	-.592**	-0.323	-0.369	-0.249	-0.243	-0.411	-0.408	-0.407	-0.400
Sst_3	GABAergic	40	-.770**	-.549*	-.721**	-.549*	-0.408	-0.320	-.614**	-.544*	-.643**	-.722**	-.667**	-.711**	-.692**	-.647**	-.671**	-.745**	-.633**	-.686**	-.630**	-.733**	-.543*	-.618**	-0.375	-0.452	-0.397	-0.367	-0.456	-0.458	-.526*	-.531*
Sst_19	GABAergic	41	-.684**	-.541*	-.714**	-.541*	-.546*	-0.402	-.602**	-.556*	-.610**	-.666**	-.647**	-.672**	-.658**	-.625**	-.621**	-.639**	-.668**	-.672**	-.552*	-.621**	-.586*	-.565*	-0.401	-0.452	-0.436	-0.427	-0.439	-0.399	-.496*	-.502*
Sst_9	GABAergic	42	-.626**	-.560*	-.663**	-.560*	-0.310	-0.245	-.545*	-0.407	-.543*	-.612**	-.489*	-.507*	-.515*	-0.453	-.515*	-.584*	-.482*	-.531*	-.549*	-.619**	-0.362	-0.448	-0.263	-0.328	-0.209	-0.182	-0.414	-0.412	-0.445	-0.432
Sst_10	GABAergic	43	-.496*	-.631**	-.744**	-.631**	-.551*	-0.436	-.597**	-0.438	-.576*	-.521*	-.497*	-.478*	-.539*	-.495*	-.538*	-.529*	-0.444	-0.426	-.486*	-.502*	-0.369	-0.346	-0.347	-0.377	-0.406	-0.328	-0.372	-0.335	-.495*	-.472*
Sst_13	GABAergic	44	-.564*	-.565*	-.680**	-.565*	-.488*	-0.365	-.509*	-0.415	-.543*	-.590*	-.497*	-.516*	-.517*	-.478*	-.497*	-.535*	-.484*	-.512*	-0.430	-.518*	-0.418	-0.429	-0.246	-0.315	-0.296	-0.257	-0.317	-0.269	-0.382	-0.373
Sst_12	GABAergic	45	-.605**	-0.455	-.600**	-0.455	-0.435	-0.335	-.505*	-0.439	-.548*	-.656**	-.545*	-.592**	-.552*	-.539*	-.535*	-.586*	-.531*	-.586*	-0.462	-.590**	-0.462	-.528*	-0.303	-0.403	-0.227	-0.227	-0.296	-0.298	-0.373	-0.401
Sst.Chodl_2	GABAergic	46	-.630**	-.549*	-.648**	-.549*	-0.333	-0.296	-.502*	-0.439	-.517*	-.572*	-.608**	-.689**	-.635**	-.670**	-.639**	-.757**	-.510*	-.582*	-.559*	-.697**	-0.420	-.591**	-0.328	-0.443	-0.295	-0.277	-0.315	-0.392	-0.446	-.480*
Sst_11	GABAergic	47	-.487*	-0.380	-.544*	-0.380	-0.390	-0.377	-.545*	-0.347	-.613**	-.541*	-.588*	-.573*	-.635**	-.611**	-.677**	-.658**	-0.390	-0.416	-.571*	-.613**	-0.386	-.486*	-.488*	-.526*	-0.418	-0.319	-0.351	-0.433	-.558*	-.558*
Sst_20	GABAergic	48	-.681**	-.502*	-.694**	-.502*	-0.424	-0.357	-.646**	-.477*	-.700**	-.692**	-.675**	-.670**	-.719**	-.661**	-.736**	-.753**	-.554*	-.578*	-.677**	-.726**	-.490*	-.568*	-0.458	-.495*	-0.470	-0.393	-.491*	-.529*	-.620**	-.623**
Sst_22	GABAergic	49	-.613**	-.593**	-.686**	-.593**	-0.419	-0.382	-.558*	-0.422	-.593**	-.593**	-.626**	-.649**	-.656**	-.642**	-.685**	-.748**	-.510*	-.557*	-.612**	-.707**	-0.431	-.580*	-0.435	-.510*	-0.399	-0.366	-0.442	-.516*	-.549*	-.554*
Sst_23	GABAergic	50	-.558*	-0.333	-.489*	-0.333	-0.394	-0.383	-.615**	-.479*	-.591**	-.585*	-.495*	-.529*	-.557*	-.555*	-.583*	-.661**	-0.380	-0.456	-.571*	-.667**	-0.343	-0.468	-0.400	-.495*	-0.338	-0.269	-0.391	-.483*	-.546*	-.538*
Sst_25	GABAergic	51	-.543*	-0.457	-.564*	-0.457	-0.313	-0.304	-0.454	-0.303	-.537*	-.560*	-.526*	-.552*	-.550*	-.526*	-.582*	-.626**	-0.380	-0.458	-.486*	-.605**	-0.363	-.524*	-0.369	-0.458	-0.251	-0.200	-0.298	-0.372	-0.422	-0.414
Sst_2	GABAergic	52	-.753**	-.515*	-.681**	-.515*	-0.306	-0.235	-.598**	-.525*	-.609**	-.692**	-.616**	-.664**	-.650**	-.605**	-.641**	-.739**	-.567*	-.628**	-.628**	-.728**	-.469*	-.577*	-0.333	-0.409	-0.358	-0.325	-0.451	-0.476	-.522*	-.535*
Pvalb_6	GABAergic	53	-.759**	-.523*	-.682**	-.523*	-0.308	-0.236	-.586*	-.495*	-.634**	-.717**	-.608**	-.637**	-.641**	-.578*	-.628**	-.711**	-.554*	-.607**	-.613**	-.715**	-0.446	-.557*	-0.336	-0.402	-0.307	-0.275	-0.432	-0.460	-.504*	-.522*
Pvalb_5	GABAergic	54	-.683**	-0.458	-.612**	-0.458	-0.415	-0.374	-.614**	-0.462	-.664**	-.687**	-.593**	-.611**	-.641**	-.606**	-.648**	-.713**	-.500*	-.559*	-.618**	-.721**	-0.427	-.551*	-0.403	-.479*	-0.316	-0.263	-0.429	-.498*	-.545*	-.541*
Pvalb_8	GABAergic	55	-.545*	-.556*	-.732**	-.556*	-0.438	-0.326	-.576*	-0.327	-.687**	-.572*	-.563*	-.473*	-.623**	-.499*	-.634**	-.559*	-0.424	-0.367	-.559*	-.510*	-0.376	-0.336	-0.356	-0.299	-0.470	-0.335	-0.432	-0.389	-.572*	-.541*
Pvalb_7	GABAergic	56	-.631**	-.566*	-.654**	-.566*	-0.379	-0.303	-.538*	-.473*	-.539*	-.622**	-0.461	-.518*	-.503*	-.493*	-.488*	-.616**	-0.416	-.484*	-0.464	-.613**	-0.299	-0.430	-0.221	-0.337	-0.180	-0.154	-0.282	-0.320	-0.386	-0.416
Pvalb_1	GABAergic	57	-.696**	-.573*	-.773**	-.573*	-0.411	-0.278	-.593**	-0.431	-.655**	-.617**	-.520*	-.507*	-.571*	-.519*	-.580*	-.573*	-0.393	-0.375	-0.451	-.471*	-.493*	-.521*	-0.410	-0.402	-0.418	-0.338	-0.392	-0.385	-.570*	-.549*
Pvalb_3	GABAergic	58	-.712**	-.494*	-.702**	-.494*	-0.282	-0.170	-.575*	-0.400	-.630**	-.616**	-.495*	-.477*	-.552*	-0.455	-.550*	-.561*	-0.413	-0.418	-.506*	-.541*	-0.383	-0.416	-0.286	-0.288	-0.267	-0.187	-0.396	-0.387	-.499*	-0.453
Pvalb_2	GABAergic	59	-.594**	-.517*	-.704**	-.517*	-0.304	-0.156	-0.456	-0.307	-.556*	-.548*	-.579*	-.558*	-.596**	-.550*	-.599**	-.550*	-.484*	-0.437	-0.454	-.475*	-0.437	-0.462	-0.333	-0.318	-0.272	-0.236	-0.293	-0.291	-0.424	-0.450
Pvalb_15	GABAergic	60	-.526*	-0.463	-.618**	-0.463	-0.272	-0.129	-0.425	-0.315	-.499*	-.550*	-0.463	-0.458	-.485*	-0.441	-.475*	-.476*	-0.404	-0.380	-0.392	-0.438	-0.311	-0.346	-0.192	-0.207	-0.181	-0.151	-0.229	-0.216	-0.332	-0.393
Pvalb_14	GABAergic	61	-.546*	-0.303	-.525*	-0.303	-0.212	-0.068	-0.404	-0.185	-.542*	-.552*	-0.431	-0.367	-0.451	-0.344	-0.460	-0.378	-0.387	-0.347	-0.392	-0.380	-0.372	-0.357	-0.221	-0.171	-0.134	-0.099	-0.329	-0.297	-0.368	-0.349
Pvalb_12	GABAergic	62	-.551*	-.554*	-.664**	-.554*	-0.434	-0.304	-.497*	-0.359	-.579*	-.627**	-0.460	-0.442	-.491*	-0.416	-.471*	-.497*	-0.435	-0.442	-0.435	-.504*	-0.325	-0.343	-0.184	-0.224	-0.221	-0.169	-0.308	-0.261	-0.369	-0.387
Pvalb_10	GABAergic	63	-.606**	-0.439	-.538*	-0.439	-0.261	-0.250	-0.462	-0.334	-.538*	-.644**	-.506*	-.532*	-.519*	-0.458	-.505*	-.569*	-.478*	-.560*	-.527*	-.633**	-0.369	-.489*	-0.275	-0.363	-0.135	-0.111	-0.317	-0.338	-0.362	-0.363
Pvalb_13	GABAergic	64	-.576*	-0.366	-.497*	-0.366	-0.182	-0.126	-0.372	-0.278	-.489*	-.617**	-0.410	-0.440	-0.426	-0.364	-0.410	-.469*	-0.344	-0.419	-0.364	-.496*	-0.284	-0.402	-0.159	-0.241	-0.050	-0.017	-0.175	-0.188	-0.245	-0.273
Pvalb_9	GABAergic	65	-.666**	-.754**	-.812**	-.754**	-.583*	-0.452	-.621**	-.530*	-.595**	-.598**	-.593**	-.592**	-.616**	-.576*	-.613**	-.674**	-.608**	-.603**	-.583*	-.652**	-.472*	-.523*	-0.407	-0.451	-0.451	-0.465	-.556*	-.554*	-.565*	-.550*
Chandelier_1	GABAergic	66	-0.339	-0.296	-0.433	-0.296	-0.429	-0.386	-0.435	-0.220	-.573*	-.518*	-0.329	-0.262	-0.369	-0.324	-0.417	-0.350	-0.132	-0.122	-0.288	-0.275	-0.339	-0.377	-0.427	-0.405	-0.390	-0.291	-0.257	-0.279	-.482*	-.505*
Chandelier_2	GABAergic	67	-0.379	-0.268	-0.413	-0.268	-0.452	-0.447	-0.459	-0.279	-.612**	-.581*	-0.431	-0.383	-0.464	-0.430	-.501*	-0.447	-0.216	-0.230	-0.368	-0.377	-0.413	-.472*	-.506*	-.509*	-0.462	-0.364	-0.264	-0.307	-.514*	-.559*

S5 File. Fig 6B. Plotted data Continue

			Pan t.		*Pan p.*		*Gorilla g.*		*Pongo a.*		*Nomascus l.*		*Macaca m.*		*Macaca f.*		*Papio a.*		*Chlorocebus s.*		*Rhinopithecus r.*		*Saimiri b.*		*Callithrix j.*		*Tarsius s.*		*Otolemur g.*		*Microcebus m.*	
**Cell type**	**Cell type**	**Sample #**	**dN**	**dN/dS**	**dN**	**dN/dS**	**dN**	**dN/dS**	**dN**	**dN/dS**	**dN**	**dN/dS**	**dN**	**dN/dS**	**dN**	**dN/dS**	**dN**	**dN/dS**	**dN**	**dN/dS**	**dN**	**dN/dS**	**dN**	**dN/dS**	**dN**	**dN/dS**	**dN**	**dN/dS**	**dN**	**dN/dS**	**dN**	**dN/dS**
L2.3.IT_1	GLUTAMAergic	68	-.549*	-.566*	-.649**	-.566*	-0.432	-0.383	-.556*	-0.348	-.575*	-.582*	-0.377	-0.352	-0.416	-0.324	-0.403	-0.418	-0.382	-0.414	-0.452	-.475*	-0.350	-0.354	-0.280	-0.316	-0.255	-0.195	-0.383	-0.328	-0.437	-0.365
L2.3.IT_2	GLUTAMAergic	69	-.602**	-0.408	-.602**	-0.408	-0.322	-0.217	-.560*	-0.347	-.627**	-.657**	-0.333	-0.269	-0.376	-0.231	-0.373	-0.333	-0.302	-0.299	-0.393	-0.376	-0.355	-0.322	-0.280	-0.254	-0.221	-0.147	-0.391	-0.325	-0.462	-0.419
L2.3.IT_6	GLUTAMAergic	70	-.541*	-.638**	-.724**	-.638**	-0.423	-0.380	-.548*	-0.337	-.577*	-.554*	-0.458	-0.433	-.489*	-0.406	-.491*	-.485*	-0.404	-0.424	-.483*	-.493*	-0.389	-0.406	-0.347	-0.374	-0.333	-0.258	-0.382	-0.335	-.474*	-0.412
L2.3.IT_7	GLUTAMAergic	71	-.600**	-.661**	-.764**	-.661**	-0.474	-0.413	-.603**	-0.401	-.623**	-.602**	-0.454	-0.429	-.493*	-0.401	-.493*	-.497*	-0.401	-0.424	-.485*	-.497*	-0.424	-0.431	-0.370	-0.395	-0.375	-0.293	-0.432	-0.375	-.525*	-0.454
L2.3.IT_5	GLUTAMAergic	72	-.525*	-.553*	-.583*	-.553*	-0.444	-0.446	-.488*	-0.305	-.541*	-.582*	-0.390	-0.384	-0.403	-0.329	-0.424	-0.467	-0.354	-0.443	-0.428	-.511*	-0.399	-.494*	-0.369	-0.445	-0.264	-0.231	-0.421	-0.415	-0.439	-0.360
L2.3.IT_13	GLUTAMAergic	73	-.568*	-.581*	-.670**	-.581*	-.624**	-.634**	-.686**	-0.463	-.682**	-.646**	-.520*	-.500*	-.559*	-.506*	-.571*	-.574*	-0.461	-.508*	-.588*	-.611**	-.494*	-.533*	-.555*	-.612**	-0.472	-0.410	-.501*	-.506*	-.627**	-.560*
L2.3.IT_10	GLUTAMAergic	74	-.501*	-.595**	-.655**	-.595**	-.547*	-.551*	-.625**	-0.410	-.610**	-.570*	-0.460	-0.439	-.502*	-0.443	-.512*	-.527*	-0.408	-0.449	-.545*	-.564*	-0.391	-0.427	-0.443	-.497*	-0.407	-0.338	-0.449	-0.441	-.554*	-.491*
L2.3.IT_8	GLUTAMAergic	75	-.585*	-.655**	-.751**	-.655**	-.502*	-0.457	-.616**	-0.407	-.631**	-.608**	-0.447	-0.416	-.485*	-0.387	-.485*	-.479*	-0.397	-0.424	-.485*	-.497*	-0.422	-0.424	-0.406	-0.435	-0.396	-0.314	-0.434	-0.380	-.529*	-0.452
L2.3.IT_12	GLUTAMAergic	76	-.564*	-.661**	-.728**	-.661**	-.510*	-.486*	-.599**	-0.405	-.606**	-.591**	-0.462	-0.447	-.495*	-0.420	-.497*	-.514*	-0.412	-0.456	-.500*	-.537*	-0.410	-0.445	-0.410	-0.464	-0.375	-0.308	-0.419	-0.390	-.512*	-0.443
L6.IT_1	GLUTAMAergic	77	-.587*	-.547*	-.680**	-.547*	-0.323	-0.255	-.564*	-0.372	-.585*	-.608**	-.550*	-.525*	-.573*	-.481*	-.575*	-.570*	-.533*	-.537*	-.610**	-.606**	-0.408	-0.431	-0.326	-0.339	-0.304	-0.262	-0.460	-0.427	-.502*	-.487*
L6.IT_2	GLUTAMAergic	78	-0.447	-.589*	-.687**	-.589*	-0.358	-0.283	-.490*	-0.259	-.557*	-.511*	-0.468	-0.409	-.499*	-0.403	-.521*	-.478*	-0.368	-0.350	-.481*	-.471*	-0.289	-0.308	-0.299	-0.293	-0.296	-0.203	-0.345	-0.315	-0.447	-0.430
L4.IT_2	GLUTAMAergic	79	-.530*	-0.423	-.540*	-0.423	-.491*	-0.473	-.660**	-0.430	-.666**	-.626**	-.531*	-.477*	-.579*	-.501*	-.607**	-.600**	-.477*	-.493*	-.666**	-.677**	-0.368	-0.429	-.496*	-.520*	-0.378	-0.325	-.554*	-.607**	-.626**	-.600**
L4.IT_3	GLUTAMAergic	80	-.611**	-.589*	-.666**	-.589*	-.553*	-.543*	-.705**	-.483*	-.679**	-.655**	-.547*	-.506*	-.585*	-.498*	-.597**	-.615**	-.551*	-.578*	-.693**	-.694**	-0.464	-.502*	-.513*	-.543*	-0.463	-0.433	-.633**	-.638**	-.663**	-.599**
L4.IT_4	GLUTAMAergic	81	-.631**	-.572*	-.699**	-.572*	-0.480	-0.383	-.617**	-0.454	-.575*	-.564*	-.620**	-.566*	-.623**	-.516*	-.623**	-.579*	-.689**	-.658**	-.662**	-.637**	-.558*	-.533*	-.518*	-.497*	-0.434	-0.456	-.650**	-.630**	-.612**	-.543*
L5.IT_1	GLUTAMAergic	82	-.492*	-.568*	-.612**	-.568*	-.650**	-.617**	-.667**	-0.433	-.670**	-.610**	-0.438	-0.362	-.482*	-0.388	-.514*	-.510*	-0.410	-0.421	-.570*	-.562*	-0.406	-0.425	-.512*	-.522*	-0.491	-0.442	-.622**	-.620**	-.660**	-.597**
L5.IT_3	GLUTAMAergic	83	-0.289	-.597**	-.578*	-.597**	-.483*	-0.466	-.516*	-0.287	-.482*	-0.375	-0.415	-0.330	-0.435	-0.377	-.505*	-.469*	-0.357	-0.320	-.536*	-0.461	-0.316	-0.353	-.489*	-0.458	-.519*	-0.490	-.573*	-.591*	-.612**	-.585*
L2.3.IT_3	GLUTAMAergic	84	-.553*	-.602**	-.625**	-.602**	-.587*	-.562*	-.498*	-0.360	-.550*	-.613**	-0.424	-0.416	-0.409	-0.335	-0.409	-0.420	-0.450	-.524*	-0.388	-0.465	-.545*	-.579*	-0.462	-.533*	-0.346	-0.352	-0.458	-0.407	-0.443	-0.354
L4.IT_1	GLUTAMAergic	85	-.630**	-.606**	-.635**	-.606**	-.535*	-.487*	-.577*	-0.413	-.632**	-.647**	-.598**	-.537*	-.591**	-.504*	-.649**	-.667**	-.597**	-.606**	-.661**	-.666**	-.607**	-.709**	-.604**	-.589*	-.596*	-.645**	-.797**	-.830**	-.724**	-.692**
L5.IT_2	GLUTAMAergic	86	-.654**	-.699**	-.762**	-.699**	-.665**	-.602*	-.703**	-.509*	-.731**	-.704**	-.696**	-.633**	-.706**	-.633**	-.750**	-.744**	-.662**	-.650**	-.744**	-.739**	-.623**	-.693**	-.679**	-.675**	-.640**	-.658**	-.779**	-.814**	-.787**	-.773**
L5.IT_5	GLUTAMAergic	87	-.581*	-.621**	-.679**	-.621**	-.764**	-.727**	-.672**	-.506*	-.671**	-.644**	-.696**	-.633**	-.669**	-.622**	-.707**	-.638**	-.720**	-.693**	-.688**	-.636**	-.795**	-.797**	-.845**	-.824**	-.758**	-.814**	-.811**	-.818**	-.801**	-.763**
L5.IT_6	GLUTAMAergic	88	-.513*	-.624**	-.626**	-.624**	-.690**	-.654**	-.577*	-0.435	-.560*	-.552*	-.620**	-.562*	-.579*	-.539*	-.627**	-.579*	-.683**	-.658**	-.629**	-.579*	-.737**	-.758**	-.764**	-.741**	-.683**	-.773**	-.800**	-.805**	-.731**	-.691**
L5.IT_7	GLUTAMAergic	89	-.541*	-.608**	-.664**	-.608**	-.655**	-.630**	-.683**	-0.462	-.673**	-.619**	-.601**	-.525*	-.607**	-.537*	-.668**	-.628**	-.573*	-.558*	-.688**	-.634**	-.616**	-.656**	-.725**	-.703**	-.684**	-.687**	-.786**	-.811**	-.799**	-.757**
L5.ET_1	GLUTAMAergic	90	-.518*	-.505*	-.529*	-.505*	-.584*	-.569*	-.566*	-0.420	-.504*	-.531*	-0.423	-0.379	-0.394	-0.331	-0.424	-0.395	-.543*	-.555*	-.505*	-0.458	-.655**	-.644**	-.643**	-.636**	-.522*	-.609*	-.725**	-.692**	-.635**	-.539*
L5.ET_2	GLUTAMAergic	91	-0.387	-.661**	-.633**	-.661**	-.499*	-0.442	-0.388	-0.238	-0.387	-0.373	-0.355	-0.284	-0.309	-0.224	-0.333	-0.251	-0.441	-0.399	-0.330	-0.234	-.584*	-.515*	-.505*	-0.444	-.531*	-.582*	-.563*	-0.449	-.490*	-0.396
L6.CT_1	GLUTAMAergic	92	-0.332	-.534*	-.593**	-.534*	-0.407	-0.300	-0.390	-0.188	-0.465	-0.393	-.584*	-.518*	-.576*	-.564*	-.643**	-.570*	-.493*	-0.416	-.523*	-.491*	-0.397	-0.460	-0.431	-0.392	-0.419	-0.437	-0.447	-.494*	-.505*	-.552*
L6.CT_2	GLUTAMAergic	93	-0.437	-.601**	-.689**	-.601**	-.566*	-0.458	-.506*	-0.316	-.550*	-.495*	-.639**	-.591**	-.633**	-.610**	-.650**	-.568*	-.610**	-.550*	-.560*	-.539*	-.506*	-.495*	-.485*	-.477*	-0.410	-0.407	-0.440	-0.432	-.512*	-.520*
L6.CT_3	GLUTAMAergic	94	-.490*	-.528*	-.678**	-.528*	-.618**	-.508*	-.623**	-0.414	-.648**	-.577*	-.675**	-.610**	-.687**	-.645**	-.704**	-.604**	-.633**	-.564*	-.639**	-.598**	-.533*	-.497*	-.570*	-.550*	-0.496	-0.470	-.517*	-.520*	-.616**	-.627**
L6.CT_4	GLUTAMAergic	95	-0.418	-.657**	-.662**	-.657**	-0.367	-0.272	-0.411	-0.213	-.490*	-0.462	-.545*	-.477*	-.543*	-.486*	-.603**	-.590**	-.484*	-0.437	-.552*	-.550*	-0.333	-0.437	-0.350	-0.326	-0.358	-0.379	-.506*	-.539*	-.499*	-.535*
L6b_1	GLUTAMAergic	96	-0.406	-.657**	-.735**	-.657**	-0.324	-0.190	-.474*	-0.303	-.539*	-0.445	-.573*	-0.466	-.570*	-0.437	-.577*	-0.456	-.529*	-0.418	-.508*	-0.418	-0.408	-0.341	-0.383	-0.295	-.502*	-0.481	-0.456	-0.374	-.487*	-.493*
L6b_2	GLUTAMAergic	97	-0.437	-.584*	-.678**	-.584*	-0.307	-0.202	-.498*	-0.297	-.583*	-.514*	-.550*	-0.468	-.573*	-0.466	-.618**	-.566*	-0.422	-0.370	-.539*	-.516*	-0.328	-0.376	-0.354	-0.314	-0.452	-0.390	-0.462	-0.460	-.529*	-.556*
L6b_3	GLUTAMAergic	98	-0.258	-0.262	-0.418	-0.262	-0.254	-0.213	-.525*	-0.284	-.565*	-0.408	-0.378	-0.255	-0.436	-0.306	-0.468	-0.337	-0.230	-0.155	-0.458	-0.356	-0.187	-0.138	-0.388	-0.311	-0.419	-0.303	-0.353	-0.356	-.509*	-.506*
L6b_4	GLUTAMAergic	99	-0.206	-0.441	-.520*	-0.441	-0.457	-0.400	-.563*	-0.338	-.547*	-0.367	-0.428	-0.320	-.475*	-0.388	-.509*	-0.399	-0.322	-0.243	-.491*	-0.397	-0.245	-0.182	-0.428	-0.378	-.500*	-0.412	-0.419	-0.402	-.548*	-.537*
L6b_5	GLUTAMAergic	100	-0.259	-0.427	-.508*	-0.427	-0.451	-0.426	-.561*	-0.342	-.576*	-0.428	-0.443	-0.364	-.494*	-0.417	-.523*	-0.452	-0.305	-0.276	-.489*	-0.456	-0.249	-0.241	-0.417	-0.414	-0.442	-0.334	-0.365	-0.372	-.518*	-.504*
L6b_6	GLUTAMAergic	101	-0.259	-0.424	-.480*	-0.424	-0.391	-0.376	-.531*	-0.333	-.528*	-0.415	-0.415	-0.362	-0.467	-0.413	-.507*	-.488*	-0.284	-0.279	-.495*	-.488*	-0.205	-0.249	-0.356	-0.376	-0.406	-0.310	-0.365	-0.397	-.499*	-.504*
L6.IT.Car3_1	GLUTAMAergic	102	-0.198	-0.291	-0.423	-0.291	-0.328	-0.277	-0.448	-0.144	-.523*	-0.400	-0.271	-0.112	-0.306	-0.159	-0.370	-0.202	-0.145	-0.062	-0.368	-0.206	-0.236	-0.151	-0.402	-0.283	-0.430	-0.325	-0.409	-0.361	-.526*	-.504*
L6.IT.Car3_2	GLUTAMAergic	103	-0.257	-0.366	-0.446	-0.366	-0.463	-.483*	-.497*	-0.178	-.573*	-0.464	-0.373	-0.238	-0.389	-0.266	-0.452	-0.305	-0.275	-0.242	-.469*	-0.337	-0.398	-0.353	-.558*	-.484*	-.531*	-0.442	-.506*	-0.472	-.599**	-.537*
L6.IT.Car3_3	GLUTAMAergic	104	-0.206	-0.422	-0.467	-0.422	-0.453	-0.431	-.486*	-0.206	-.522*	-0.417	-0.386	-0.252	-0.402	-0.306	-.471*	-0.353	-0.307	-0.238	-.500*	-0.361	-0.327	-0.296	-.495*	-0.412	-.528*	-0.475	-.529*	-.511*	-.601**	-.602**
L5.6.NP_1	GLUTAMAergic	105	-.567*	-.686**	-.857**	-.686**	-0.373	-0.218	-.511*	-0.322	-.550*	-0.429	-.621**	-.542*	-.629**	-.526*	-.640**	-.498*	-.547*	-0.435	-.505*	-0.409	-.525*	-0.437	-.488*	-0.393	-0.493	-0.445	-0.452	-0.382	-.551*	-.494*
L5.6.NP_2	GLUTAMAergic	106	-.567*	-.728**	-.872**	-.728**	-0.346	-0.199	-.513*	-0.311	-.546*	-0.429	-.590*	-.505*	-.601**	-.481*	-.621**	-.500*	-.519*	-0.416	-.517*	-0.422	-.478*	-0.410	-0.450	-0.355	-0.484	-0.433	-.489*	-0.417	-.559*	-.496*
L5.6.NP_3	GLUTAMAergic	107	-.550*	-.688**	-.855**	-.688**	-0.407	-0.255	-.517*	-0.307	-.569*	-0.435	-.632**	-.540*	-.639**	-.530*	-.655**	-.500*	-.553*	-0.437	-.519*	-0.415	-.531*	-0.437	-.505*	-0.404	-.513*	-0.460	-0.472	-0.400	-.568*	-.507*
L5.6.NP_6	GLUTAMAergic	108	-0.444	-.740**	-.826**	-.740**	-0.321	-0.181	-0.424	-0.210	-0.462	-0.339	-.482*	-0.380	-.486*	-0.354	-.511*	-0.384	-0.428	-0.322	-0.424	-0.316	-0.384	-0.301	-0.358	-0.256	-0.426	-0.377	-0.440	-0.342	-.473*	-0.399
L5.6.NP_4	GLUTAMAergic	109	-0.358	-.642**	-.766**	-.642**	-0.471	-0.343	-.496*	-0.245	-.526*	-0.337	-.507*	-0.384	-.528*	-0.416	-.552*	-0.371	-0.418	-0.288	-0.448	-0.307	-0.394	-0.258	-0.462	-0.352	-.519*	-0.426	-0.412	-0.327	-.533*	-0.465
Astro_1	Non-Neuronal	110	-0.168	-0.216	-0.174	-0.216	-.577*	-.540*	-0.341	-0.302	-0.372	-0.458	-0.296	-0.247	-0.265	-0.263	-0.291	-0.302	-0.380	-0.372	-0.343	-0.328	-0.422	-0.427	-0.401	-0.404	-0.443	-.529*	-.490*	-.487*	-0.435	-.521*
Astro_2	Non-Neuronal	111	-0.191	-0.226	-0.200	-0.226	-.591*	-.548*	-0.368	-0.316	-0.406	-.482*	-0.315	-0.252	-0.286	-0.265	-0.312	-0.307	-0.393	-0.377	-0.364	-0.336	-0.440	-0.430	-0.427	-0.417	-0.479	-.554*	-.520*	-.508*	-0.466	-.545*
Astro_3	Non-Neuronal	112	-0.165	-0.234	-0.171	-0.234	-.574*	-.546*	-0.333	-0.323	-0.341	-0.438	-0.283	-0.255	-0.252	-0.270	-0.276	-0.315	-0.372	-0.377	-0.330	-0.336	-0.406	-0.432	-0.388	-0.411	-0.429	-.526*	-0.468	-0.477	-0.417	-.508*
Astro_4	Non-Neuronal	113	-0.160	-0.247	-0.185	-0.247	-.569*	-.526*	-0.345	-0.350	-0.317	-0.404	-0.257	-0.236	-0.234	-0.265	-0.255	-0.307	-0.357	-0.351	-0.317	-0.315	-0.383	-0.398	-0.359	-0.380	-0.436	-.533*	-0.465	-0.471	-0.419	-.513*
Astro_5	Non-Neuronal	114	-0.165	-0.234	-0.171	-0.234	-.574*	-.546*	-0.333	-0.323	-0.341	-0.438	-0.283	-0.255	-0.252	-0.270	-0.276	-0.315	-0.372	-0.377	-0.330	-0.336	-0.406	-0.432	-0.388	-0.411	-0.429	-.526*	-0.468	-0.477	-0.417	-.508*
OPC_1	Non-Neuronal	115	-0.324	-0.361	-0.436	-0.361	-0.356	-0.185	-0.234	-0.109	-0.358	-0.396	-0.161	-0.022	-0.133	0.046	-0.147	-0.029	-0.210	-0.137	-0.079	-0.020	-0.379	-0.271	-0.250	-0.133	-0.275	-0.308	-0.369	-0.257	-0.311	-0.262
OPC_2	Non-Neuronal	116	-0.319	-.544*	-.518*	-.544*	-0.478	-0.352	-0.268	-0.207	-0.376	-0.448	-0.361	-0.287	-0.304	-0.236	-0.326	-0.270	-0.409	-0.350	-0.247	-0.213	-.497*	-.472*	-0.375	-0.319	-0.445	-.525*	-0.449	-0.369	-0.377	-0.424
Oligo_1	Non-Neuronal	117	-0.091	0.009	-0.103	0.009	-0.390	-0.282	-0.174	-0.022	-0.278	-0.195	-0.168	-0.020	-0.166	-0.056	-0.187	-0.035	-0.182	-0.090	-0.121	-0.004	-0.388	-0.239	-0.307	-0.161	-0.340	-0.340	-0.324	-0.275	-0.364	-0.299
Oligo_2	Non-Neuronal	118	-0.167	-0.091	-0.133	-0.091	-0.225	-0.069	0.041	0.097	-0.154	-0.142	-0.043	0.086	-0.015	0.123	0.001	0.121	-0.112	-0.022	0.062	0.151	-0.269	-0.151	-0.034	0.086	-0.110	-0.139	-0.224	-0.129	-0.116	-0.076
Oligo_4	Non-Neuronal	119	-0.027	0.138	0.038	0.138	-0.027	0.080	0.150	0.230	-0.058	-0.133	0.043	0.157	0.088	0.230	0.091	0.227	0.021	0.061	0.198	0.238	-0.228	-0.129	-0.006	0.112	0.054	0.025	0.023	0.102	0.027	0.062
Oligo_3	Non-Neuronal	120	0.094	0.210	0.087	0.210	-0.212	-0.080	-0.043	0.085	-0.130	-0.085	0.067	0.230	0.064	0.181	0.043	0.203	0.045	0.157	0.078	0.214	-0.156	0.016	-0.102	0.064	-0.149	-0.149	-0.135	-0.082	-0.190	-0.179
Endo_1	Non-Neuronal	121	0.149	0.094	0.059	0.094	-0.026	0.013	0.164	0.440	-0.056	-0.129	0.040	0.170	0.071	0.170	0.011	0.148	0.043	0.081	0.043	0.059	0.119	0.179	0.094	0.214	0.289	0.289	0.050	0.112	0.148	0.173
Endo_2	Non-Neuronal	122	0.120	0.123	0.030	0.123	-0.121	-0.019	-0.037	0.154	-0.113	-0.157	-0.103	0.027	-0.100	-0.030	-0.132	-0.024	-0.186	-0.062	-0.214	-0.084	0.033	0.208	0.065	0.243	-0.022	-0.022	-0.170	-0.076	-0.081	-0.141
Endo_3	Non-Neuronal	123	0.031	0.041	-0.043	0.041	0.003	0.083	-0.105	0.105	-0.147	-0.173	-0.085	0.031	-0.112	-0.028	-0.163	-0.119	-0.118	-0.020	-0.292	-0.164	0.108	0.200	0.111	0.272	-0.048	-0.019	-0.237	-0.178	-0.167	-0.224
VLMC_1	Non-Neuronal	124	-0.071	-0.211	-0.258	-0.211	-0.205	-0.051	-0.304	-0.304	-0.117	-0.023	-0.070	-0.023	-0.117	-0.117	-0.117	-0.117	-0.164	-0.023	-0.210	-0.070	-0.023	0.117	-0.023	0.070	-0.308	-0.308	-0.306	-0.255	-0.304	-0.351
VLMC_2	Non-Neuronal	125	0.151	-0.235	-0.267	-0.235	-0.062	0.044	-0.011	0.061	-0.026	0.181	-0.210	-0.106	-0.206	-0.206	-0.242	-0.062	-0.094	0.110	-0.091	0.113	-0.070	0.062	-0.178	-0.006	-0.363	-0.320	-0.053	-0.018	-0.191	-0.259
Micro.PVM_1	Non-Neuronal	126																														
Micro.PVM_2	Non-Neuronal	127	-0.307	-0.351	-0.351	-0.351	0.154	0.257	0.070	-0.023	0.023	-0.117	-0.117	-0.164	-0.070	-0.023	-0.023	-0.070	-0.210	-0.210	0.023	-0.023	-0.164	-0.164	0.164	0.164	0.084	0.028	0.000	0.102	0.117	0.117

Negative Correlation, * $p < 0.05$; ** $p < 0.01$ ** (2-tailed).

Spearman's correlation analysis conducted in SPSS (see Methods).

Fig 6B. scRNA expression in human cells used for correlation analysis

Cell sample #	1	2	3	4	5	6	7	8	9	10	11	12	13	14	15	16
gene	Lamp5_Lhx6_1	Lamp5_1	Lamp5_2	Lamp5_3	Lamp5_4	Lamp5_5	Lamp5_6	Pax6_1	Pax6_2	Pax6_3	Pax6_4	Sncg_3	Sncg_6	Sncg_2	Sncg_1	Sncg_8
CDH1	0	0	0	0	0	0	0	0	0	0	0	0	0	0	0	0
CDH2	7.343714233	5.50388349	6.925550315	6.823134902	6.588792098	6.214927882	6.151910691	6.790817483	7.773346204	7.055831552	6.67971898	9.498044824	6.698771928	7.02280606	6.647704034	7.380776381
CDH3	0	0	0	0	0.175406662	0	0	0	0	0.728503147	0	0	0	0.493265961	0	0
CDH4	6.626599683	8.189493484	5.50183451	4.115871959	0	0	0	9.533197364	9.795994832	6.320302264	7.665666125	2.538883452	9.305391745	8.278786302	2.826094422	7.296412879
CDH5	0	0	0	0	0	0	0	0	0	0	0	0	0	0	0	0
CDH6	0	0	0	0	0	0	0	0	0	0	0	0	0	0	0	0
CDH7	0	0	0	0	0	0	0	0	0	0	0	1.621374509	5.630808979	3.48547045	0	6.910620029
CDH8	1.602397049	3.184552978	7.712189398	7.807379682	7.358276068	2.825315078	0.185536718	5.093612573	6.801252344	8.901709663	8.460967123	8.292404813	9.093644715	6.241425089	0	6.613610668
CDH9	3.203429173	0	0	4.054680807	8.275530018	8.562979646	9.111619491	0	5.879621803	0.944484357	0	10.10895365	9.33017323	9.220325794	9.338522696	9.558974992
CDH10	0	0	0	0	0.085952611	2.975758239	7.382805171	7.575943684	8.769458707	5.161274917	7.782820012	6.217891432	7.972679266	8.633327504	8.562314723	8.608736355
CDH11	0	0	0	2.228343661	2.383116862	5.462545342	6.426102612	1.274692132	0	6.327664511	8.112060531	7.993461043	8.276187259	2.458464818	3.66836232	1.102423546
CDH12	0.742237124	0	0	0	0	0	1.528433421	10.3360416	3.986602186	0	2.231422049	4.933615901	8.921466184	10.04070059	9.518420745	10.51001194
CDH13	8.575858323	5.365791499	11.07007821	8.705904065	0	0	0	8.784121476	0	9.072746886	7.944874494	8.675826811	7.051616719	0	0	3.104964919
CDH18	3.646474038	8.948705569	8.295696502	7.521607765	4.996568144	6.725440122	4.822787531	8.508859963	8.766456469	10.00099407	8.697136663	8.710967609	7.99875299	5.829785289	4.055022511	3.57759084
CDH19	0	0	0	0	0	0	0	0	0	0	0	0	0	0	0	0
CDH20	7.784888561	7.088351146	7.263491526	7.364801533	7.918081787	8.037326716	7.323381681	6.726095134	8.065717814	0	3.96022558	0	0.91357779	0	1.281737721	0
CDH22	1.550421736	0	0	0	2.283384276	0.500304858	0	2.103743737	2.405913562	0	0	2.11623524	0	0	0	0
CDH24	0	0	0	0	0	0	0	1.593536555	1.85021477	3.738721343	0.512878489	0	0	0	0	0

Fig 6B. scRNA expression in human cells used for correlation analysis. Cont.

Cell sample #	17	18	19	20	21	22	23	24	25	26	27	28	29	30	31	32
gene	Sncg_4	Sncg_5	Vip_14	Vip_15	Vip_16	Vip_4	Vip_5	Vip_6	Vip_9	Vip_2	Vip_11	Vip_13	Vip_1	Vip_12	Vip_18	Vip_19
CDH1	0	0	0	0	0	0	0	0	0	0	0	0	0	0	0	0
CDH2	3.894895817	4.663728631	5.629546309	8.109096078	8.067232748	7.071426581	7.686390166	5.736799175	5.309270433	7.628384112	7.772163094	7.705958783	8.332322413	7.968680659	8.255936745	7.770801701
CDH3	0	0	0	0	0.739945854	0	0	0	0	0	0	0	0	0	0	0
CDH4	8.617424753	9.190866727	5.868306487	0.509738873	6.452990596	3.557399975	2.848083465	6.363377716	6.857877149	5.923298563	7.551037165	6.919183641	5.695148191	5.628653494	5.149809743	1.617113245
CDH5	0	0	0	0	0	0	0	0	0	0	0	0	0	0	0	0
CDH6	0	0	0	0	0	0	0	0.265709659	0	0	0	0	0	0	0	0
CDH7	7.417682222	7.421177707	0	0	5.171906428	0	1.023301368	6.988040547	6.949585277	0	2.007253594	1.823946447	2.802852342	1.363153467	7.140483376	3.41441772
CDH8	8.779716929	8.608878752	1.654207066	0.530868529	8.161715664	9.725021991	9.507023685	7.902647316	1.8714779	1.454985144	0	0	3.838430029	7.510289259	7.08645773	7.576371205
CDH9	11.12080845	9.630435745	8.088004242	7.40436449	8.673766003	6.040038342	9.840737904	9.667590248	7.295838497	0	0	0	8.232162585	5.975673338	9.437656003	7.493606588
CDH10	7.955777293	8.024194487	9.449267185	8.767364769	9.102962774	8.886758887	8.698442443	8.380320171	8.239464174	7.963484987	8.533557831	9.072101344	9.330551084	9.328656495	9.229123197	9.00787083
CDH11	0.668806234	0.568195249	0	0	5.138947589	2.792208496	8.032550751	7.028443884	6.8984994	0	1.299950718	0	1.298263096	0	0	0.915939813
CDH12	10.4448121	10.09369524	0.223250478	0.06821148	2.771565578	0.408001298	3.225755801	0.66417379	1.666043602	5.889956137	7.735862661	8.964462742	5.966781673	1.438979192	9.017213042	5.387365911
CDH13	0	0	8.416626822	0.814473627	9.417505111	5.877369791	7.883658748	7.995481328	8.293403818	10.43683445	10.57937354	10.33960237	10.68710013	8.904229547	10.46626049	6.945924883
CDH18	0.959171164	4.357091725	7.723484964	7.897321229	10.07466968	7.576637121	8.423218143	10.04381065	9.312771468	7.274966814	5.686727397	5.242133963	7.872084973	7.516351451	8.412103407	6.400941345
CDH19	0	0	0	0	0	0	0	0	0	0	0	0	0	0	0	0
CDH20	3.563356147	0	2.80909504	4.774123521	0.074711072	7.882900965	4.08079637	7.973655191	8.724940479	7.859567415	5.44180343	1.386724825	0	0	0	0
CDH22	0	0	0	0	0.149099472	0	0	0	0	0	0	0	0	0	0	0
CDH24	0	0	0	0	0	0	0	0	0	0	0	0	0	0	0	0

Fig 6B. scRNA expression in human cells used for correlation analysis. Cont.

Cell sample #	33	34	35	36	37	38	39	40	41	42	43	44	45	46	47	48
gene	Vip_21	Vip_23	Sst.Chodl_1	Sst_1	Sst_4	Sst_5	Sst_7	Sst_3	Sst_19	Sst_9	Sst_10	Sst_13	Sst_12	Sst.Chodl_2	Sst_11	Sst_20
CDH1	0	0	0.70968701	0	0	0	0	0	0	0	0	0	0	0	0	0
CDH2	5.591932625	7.40134881	2.895254215	9.083251844	9.387620809	9.657594308	9.489500657	8.392903303	8.473047652	7.796956321	7.30571453	8.006699691	9.438538346	7.494814976	8.029573862	8.574482349
CDH3	0	0	0	0	0.655299383	0	0	2.441059585	1.429262714	0.540091341	1.883394469	2.670135893	1.606254099	0.910055274	1.684410419	1.639525399
CDH4	2.335970902	0	7.094485764	0	5.721672128	7.489600998	4.337106565	7.042862296	5.581240779	5.37110302	3.968152973	4.843540997	5.174163562	9.171736212	3.041953612	2.449535818
CDH5	0	0	0	0	0	0	0	0	0	0	0	0	0	0	0	0
CDH6	2.262025622	0	0	0	0	0	0	0	0	0	1.149083915	0	0	5.937773726	3.185701903	0.465381269
CDH7	0	0	7.062799686	0	1.626632489	3.342936617	2.350734868	8.059371499	6.812824151	2.05423148	0	0	2.033741409	7.715628106	4.912643896	7.62519571
CDH8	8.047912189	1.878550044	3.124115729	0	9.259149041	9.516719115	8.734035878	9.095334962	9.044119432	5.78320852	7.525519046	9.067696508	5.160859748	9.436398357	9.591288014	9.897373952
CDH9	1.85545969	5.098509007	1.961378486	11.38987332	11.21421224	10.49257184	7.20446086	9.605954125	9.977013066	8.887475908	9.163401711	10.96667408	10.68002684	9.666629931	10.30003425	10.22399517
CDH10	8.192767864	8.445643681	8.484012429	5.477413324	7.931031253	9.311855465	5.575120075	8.679905674	8.603682735	7.69985019	7.481478658	7.325556314	7.140992836	7.390878684	8.78075178	8.99516889
CDH11	5.493518098	0.464967457	7.635500299	0	5.577529936	3.388531884	0	4.403365705	7.391711402	3.139443517	7.723901039	7.224205672	1.256249088	6.649610463	1.44742152	4.244413444
CDH12	5.600639419	1.510738966	6.72852	8.507667146	4.24153472	7.132940191	8.216604579	8.452895405	0	10.30104259	5.026655176	0.570272179	0.623197883	10.24475232	11.69581607	11.76874325
CDH13	0	0	0	10.22832604	11.32557447	11.1453115	10.61082332	10.77847839	10.60623161	9.884219704	5.043684476	10.43025476	10.57060433	8.736218812	0	4.44329124
CDH18	8.872958336	7.788409442	7.150506525	8.001171583	7.337966091	5.921279825	5.621006072	7.991446985	7.347457965	9.258872634	9.005973835	7.663494889	8.232743716	8.087413569	9.177800155	8.341492549
CDH19	0	0	0	0	0	0	0	0	0	0	0	0	0	0	0	0
CDH20	8.39110816	8.541755856	0	0	0	0	0	0	0	0	0	0	0	0	0	0
CDH22	0	0	0	0	0	0	0	0	0	1.576081393	1.029563685	0	0	0	0	0
CDH24	0	0	0	0	0	0	0	0	0	0	0	0	0	0	0	0

Fig 6B. scRNA expression in human cells used for correlation analysis. Cont.

Cell sample #	49	50	51	52	53	54	55	56	57	58	59	60	61	62	63	64
gene	Sst_22	Sst_23	Sst_25	Sst_2	Pvalb_6	Pvalb_5	Pvalb_8	Pvalb_7	Pvalb_1	Pvalb_3	Pvalb_2	Pvalb_15	Pvalb_14	Pvalb_12	Pvalb_10	Pvalb_13
CDH1	0	0	0	0	0	0	0	0	0	0	0	0	0	0	0	0
CDH2	8.269060453	6.975597468	7.802466404	7.829012717	6.800856146	6.592404471	5.868520367	1.401896309	3.516549893	3.789314484	5.549082295	6.613530087	7.24474645	7.167344938	7.845104993	7.179597813
CDH3	1.847665219	1.82214019	1.45371632	0.967272283	0	0	0	0	0	0	0	0	0	0	0	0
CDH4	8.434494045	3.303270613	7.488847474	4.15187032	1.825862957	1.977985758	0	6.759091898	1.540816476	0	0.467732691	2.891564688	0	1.466736712	4.887741437	1.197929113
CDH5	0	0	0	0	0	0	0	0	0	0	0	0	0	0	0	0
CDH6	3.552566128	0	0.264414619	0	0	0	0.064949133	0	3.666744705	0	1.782232823	1.381056845	0	0	0	0
CDH7	6.535130997	7.351940524	7.424288668	8.240783509	7.634083685	7.572738717	0	0.506675235	4.387448567	7.094296198	6.952424845	3.840905792	6.743663351	0	2.326917393	3.272291027
CDH8	9.868176757	9.122805432	9.433292495	8.425511089	6.065332285	7.80492385	8.51787462	0.142678327	4.117797883	7.205423539	1.747651477	0	3.773477643	1.219109679	1.008383144	0
CDH9	9.682482148	9.692290168	9.79266723	9.654287917	9.648276392	9.003799079	9.092898194	10.57653024	7.865649978	8.981227689	7.931671924	8.443932542	4.805997158	9.296528525	8.555853631	9.486489206
CDH10	8.610662054	8.243482567	8.103027419	8.760366057	7.676293982	4.872076477	5.105003568	5.133389132	2.604438647	1.677749045	0.40716826	2.148596053	0	3.332470138	5.280787707	3.431007506
CDH11	4.20067578	0.692596988	0	2.106748524	0.62113467	0.188653102	6.675171058	2.871547477	7.817041972	4.674820096	5.628611111	7.278751289	4.105129519	5.299267538	0	0
CDH12	11.76281967	11.52514698	11.90809858	11.1870634	10.78317237	10.95646688	9.93906062	8.114643906	7.871578546	9.523276219	5.161630024	4.431089493	4.412922512	3.409713284	9.549787286	9.391922112
CDH13	5.827587753	0	3.183370027	10.28843951	10.25167642	1.395269781	0.954991152	7.423789819	4.937707572	8.271466373	8.089655351	8.597395701	6.794125164	9.164562983	9.53415911	9.528518378
CDH18	9.790684504	8.663110161	10.05182003	7.689034709	7.604196106	8.679978902	5.628441596	8.204017652	10.47912439	9.275417674	9.191451938	8.434019713	8.374059572	6.91436165	7.456531395	8.244681461
CDH19	0	0	0	0	0	0	0	0	0	0	0	0	0	0	0	0
CDH20	0	0	0	0	0	0	0	0	3.901471725	0	0	0	0	0	0	0
CDH22	0	0	0	0	0	0	0	0	0	0	0	0	0	0	0.128178983	0
CDH24	0	0	0	0	0	0	0	0	0	0	0	0	0	0	0	0

Fig 6B. scRNA expression in human cells used for correlation analysis. Cont.

Cell sample #	65	66	67	68	69	70	71	72	73	74	75	76	77	78	79	80
gene	Pvalb_9	Chandelier_1	Chandelier_2	L2.3.IT_1	L2.3.IT_2	L2.3.IT_6	L2.3.IT_7	L2.3.IT_5	L2.3.IT_13	L2.3.IT_10	L2.3.IT_8	L2.3.IT_12	L6.IT_1	L6.IT_2	L4.IT_2	L4.IT_3
CDH1	0	0	0	0	0	0	0	0	0	0	0	0	0	0	0	0
CDH2	0.704019309	6.096828524	7.028497569	7.640386767	7.796886468	7.983702407	8.069155143	8.14686588	7.7187763	7.782961944	7.570598238	7.921485771	8.93903837	8.608657039	8.06939495	8.281858237
CDH3	0	0	0	0	0	0.189382652	0.124989789	0.266452595	0.514365001	0.172248779	0.074802995	0.448538221	0.395017496	0.518241083	0	0
CDH4	1.007904872	2.457031201	3.073406265	1.375421706	0	4.807522408	1.990539802	6.308209265	6.628526675	6.002450519	4.905078932	6.056329018	3.607029228	6.207657483	0.661781743	2.915618863
CDH5	0	0	0	0	0	0	0	0	0	0	0	0	0	0	0	0
CDH6	0	7.157065127	8.001123737	0	0	3.232549573	0.259898351	0	1.437572988	1.627058351	1.63440509	2.251965205	0.288565878	6.277959606	0	0
CDH7	0.062786586	0.662456053	2.105635555	0	3.248530836	0	0	0	1.286156478	0	0	0	0.607737192	0	5.95673976	2.25509032
CDH8	8.89056108	0	0	7.224619244	0	8.234416081	8.594422739	8.939744362	8.212644912	8.263088749	8.195588895	8.247388128	6.552801809	6.662191493	7.397813303	9.296459662
CDH9	3.067432027	7.797586721	8.451405192	9.46105779	8.975603315	9.524693741	9.399426813	7.577560116	8.405190874	8.958250218	9.388025191	9.010217442	8.74495948	8.819845076	7.40146695	7.187635565
CDH10	5.328164374	2.561222672	7.051490615	6.115840201	4.998830974	7.7534765	8.1784936	8.742036921	8.113062978	8.173394325	8.613262561	8.960523505	8.032863705	8.302957127	8.86021783	8.437780923
CDH11	8.443814796	2.679263285	1.687349196	6.673908665	4.72468344	6.581310056	7.353905174	0	4.96440939	4.538532372	6.510091311	4.876087677	6.208740962	7.3125465	5.056871296	3.026901993
CDH12	0.606141093	10.28741268	10.1646453	10.32451438	9.938509922	10.52297073	10.73605263	9.280436187	10.18883218	10.05730961	10.02173053	9.397550432	10.74373756	10.39580577	9.791963447	9.711373022
CDH13	5.046284832	0	0	8.955089483	9.340774125	8.3908063	9.393796793	7.250643856	1.814344907	4.464359856	8.23143005	8.020400613	10.07059666	8.290508825	0	2.689654774
CDH18	9.464360761	8.058562629	6.473568218	10.34825415	10.26299619	10.30776492	10.60612351	9.62174975	10.37410132	10.05019058	10.48431585	9.501938041	8.618646192	8.927779468	9.85022675	9.708821013
CDH19	0	0	0	0	0	0	0	0	0	0	0	0	0	0	0	0
CDH20	0	8.439062741	9.257853549	0.108934648	3.582566466	1.495664969	4.079039831	6.228821138	6.163356207	2.805757031	5.268493883	3.9124872	0	0	0	0.679556402
CDH22	0	0	0	7.198039543	2.842320065	7.182393647	6.298442584	5.969236049	6.993854689	6.779547627	6.580133224	6.0715644	5.880661637	4.179328708	0.25560814	6.088586591
CDH24	0	0	0	0	0	0	0	0	0	0	0	0	0	0	0	0

Fig 6B. scRNA expression in human cells used for correlation analysis. Cont.

Cell sample #	81	82	83	84	85	86	87	88	89	90	91	92	93	94	95	96
gene	L4.IT_4	L5.IT_1	L5.IT_3	L2.3.IT_3	L4.IT_1	L5.IT_2	L5.IT_5	L5.IT_6	L5.IT_7	L5.ET_1	L5.ET_2	L6.CT_1	L6.CT_2	L6.CT_3	L6.CT_4	L6b_1
CDH1	0	0	0	0	0	0	0	0	0	0	0	0	0	0	0	0
CDH2	8.336522134	7.529836675	7.206795005	8.307592733	8.045706663	7.80765712	7.807674784	8.05405641	7.808633118	7.139241458	6.520522796	8.623085506	8.585763803	8.835166672	8.590567983	8.637284154
CDH3	0.020139152	0	0.139360104	0.601565449	0	0	0.25490682	0.621321811	0.470918149	0.431589781	0.236992444	0.782457522	0.189969075	0.27654442	0	0
CDH4	1.12387111	2.892792939	5.966176703	5.47477422	5.105596071	6.160896298	6.876423471	6.793285647	6.907779212	6.024148344	5.732194912	7.185524478	6.665145942	6.615195691	8.17792693	0.998307879
CDH5	0	0	0	0	0	0	0	0	0	0	0	0	0	0	0	0
CDH6	0	0	3.845192107	0	0	1.560701691	2.619202666	1.101635145	5.225405064	0	5.935326369	6.449719842	6.504249538	6.45528936	7.207171163	8.371248737
CDH7	5.268428988	0	0	0	7.375393629	5.961064165	7.00055408	6.725092529	6.116239588	7.040625221	0	3.50855546	3.315411149	6.74351285	5.189058556	0.931606319
CDH8	9.313864583	7.895013378	7.550065573	8.931332081	9.263423765	8.165678072	8.903871349	9.049775735	8.409101568	9.163378309	7.790039749	8.461476503	8.296076786	7.782276106	7.848269731	1.361931864
CDH9	2.422821585	5.938751101	1.903172504	6.760088577	0	3.645274396	0.542264434	0	5.09586165	0	0	2.925551599	7.241743138	7.81143428	6.661265738	8.327035476
CDH10	8.87533343	9.28639165	9.079469342	8.996407473	8.898131251	9.690032565	9.105224883	9.022628562	8.930069425	7.785270118	6.891989096	2.878352045	4.341018931	6.97128697	7.654502014	10.05573717
CDH11	4.426965733	7.480975282	7.810134514	0	4.401878511	6.659845459	6.782055736	6.356783455	7.030124137	4.519634516	6.200246699	8.520145137	8.112155965	7.954497473	8.326451353	9.045952439
CDH12	1.991307038	9.191139196	9.425713905	2.974964095	8.614077267	8.148884796	1.736875051	0.790364216	8.222705716	2.818961807	4.199305874	4.335911897	1.94258097	1.394513441	8.970298115	7.341272897
CDH13	8.340654858	0	0	8.383081279	3.878680155	2.435387812	2.045314272	3.625918696	0.231443538	6.108180583	9.063348017	2.717262393	6.566155972	4.569510192	7.553103494	10.42017714
CDH18	9.061962899	9.604192106	9.267240463	9.710478814	8.202304437	8.575447099	8.723152577	8.858241922	8.755177134	10.17223418	8.617764575	10.64410752	10.14585077	10.04161393	10.03055698	8.6740025
CDH19	0.900114411	0	0	0	0	0	0	0	0	0	0	0	0	0	0	0
CDH20	0	3.703108948	3.128497561	7.596855204	7.377602577	4.410427282	7.861084642	8.15274069	7.332041926	9.937891937	9.382282089	0	0	0	0	0
CDH22	3.998234846	2.518308845	3.641362306	4.235759674	0	0.214274296	3.470063701	4.0380181	5.698062731	7.281187573	7.165153557	0	2.321611806	2.209639934	0	1.357133008
CDH24	0	0	0	0	0	0	0	0	0	0	0	0	0	0	0	5.914435046

Fig 6B. scRNA expression in human cells used for correlation analysis. Cont.

**Cell sample #**	97	98	99	100	101	102	103	104	105	106	107	108	109	110	111	112
gene	L6b_2	L6b_3	L6b_4	L6b_5	L6b_6	L6.IT.Car3_1	L6.IT.Car3_2	L6.IT.Car3_3	L5.6.NP_1	L5.6.NP_2	L5.6.NP_3	L5.6.NP_6	L5.6.NP_4	Astro_1	Astro_2	Astro_3
CDH1	0	0	0	0	0	0	0	0	0	0	0	0	0	0	0	0
CDH2	8.875623817	8.355144559	8.114995629	8.739264802	9.068909042	8.56623867	8.923444664	8.829976286	6.552171719	6.374784281	5.649246816	4.943653938	4.24563235	8.014645791	7.941503498	5.376235761
CDH3	0.588051743	0	0.347140039	0.23840597	0.691235027	0.032088293	0.483736034	0.008928378	0.096927033	0.380483919	0.205290616	1.527157881	0.403855119	0	0	0
CDH4	6.772307293	0	0.55002772	3.962023541	6.328616133	0	0.174606124	1.257866172	0	0	0	0	0	7.29730502	6.082323482	5.609687698
CDH5	0	0	0	0	0	0	0	0	0	0	0	0	0	0	0	0
CDH6	8.216134908	7.365621053	6.792560755	6.664492529	3.95896841	3.44165367	3.42325262	5.760092743	8.511934317	7.870368189	7.9115617	7.317594588	8.010879616	0	0	0
CDH7	2.714788809	3.779283201	0	0	0	0	0	0	7.065399961	6.1271234	4.149749894	0	0	0	0	0
CDH8	6.794012833	0	4.204075495	6.775239851	6.839857995	0	2.323693738	0.561649985	9.72096262	9.71818861	9.658312426	9.653163235	8.956869954	0	0	0
CDH9	8.758868484	9.005474177	8.40928234	8.995174382	9.701003871	2.630580568	2.189494713	1.173756298	7.645804298	6.669035596	6.406132595	4.711301187	5.336868547	0	0	0
CDH10	9.989292049	10.81789236	10.54809659	10.45321655	9.878037917	8.607390569	8.47067373	8.606215145	8.335207139	8.218797704	6.927939915	7.390219659	6.808938159	5.455593975	6.880777081	1.502665728
CDH11	8.766091176	8.933524661	8.93572671	8.472327504	8.206265048	4.660857324	1.880659095	6.625935829	8.955260209	8.487119566	8.635728252	8.918478645	9.275808626	3.032964224	3.143355399	1.451093051
CDH12	10.06673306	9.859488933	7.85927851	8.828585722	10.41759443	10.64329241	10.94435298	10.54714015	3.877228274	7.711831383	2.909309168	7.215542779	1.413541358	0	0	0
CDH13	8.243514218	0	0	0	0	0	0	0	9.960863552	10.27691236	8.965936963	9.960254096	3.603973222	0	0	0
CDH18	9.978156203	9.155754475	8.807838332	8.831339295	9.390202486	7.243890699	5.67592681	6.491192899	10.37125387	10.47374659	10.65870867	10.60417571	10.48641257	0	0	0
CDH19	0	0	0	0	0	0	0	0	0	0	0	0	0	0	0	0
CDH20	0	0	0	0	0	3.332304547	5.640439739	3.633819222	0	0	0	0	0	10.91563786	10.6972513	10.65514898
CDH22	0	0.809472062	1.19389921	0.381282333	0.28782974	1.481124061	3.579715118	1.897735623	2.150207343	1.622714533	0.946321543	3.543432661	3.012016506	0	0	0
CDH24	5.373570073	4.498243872	4.069757289	4.405236212	2.581657157	0	0	0	0	0	0	0	0	0	0	0

Fig 6B. scRNA expression in human cells used for correlation analysis. Cont.

**Cell sample #**	113	114	115	116	117	118	119	120	121	122	123	124	125	126	127
gene	Astro_4	Astro_5	OPC_1	OPC_2	Oligo_1	Oligo_2	Oligo_4	Oligo_3	Endo_1	Endo_2	Endo_3	VLMC_1	VLMC_2	Micro.PVM_1	Micro.PVM_2
CDH1	0	0	0	0	0	0.270403406	0	0	0	0	0	0	0	0	0
CDH2	3.158416822	7.182268963	2.986361316	6.288530822	0.900425009	1.073981443	0.568246032	0.319479627	3.144763564	4.07415426	6.69120028	0	0	0	0
CDH3	0	0	0	0	0	0	0	0	0	0	0	0	0	0	0
CDH4	5.905147264	8.378027359	0	4.681506047	0	0	0	0	0	0	0	0	0	0	0
CDH5	0	0	0	0	0	0	0	0	1.593591137	2.726356578	1.801192046	0	0	0	0
CDH6	0	0	0	0.131399128	0	0	0	0	0	0	0	0	8.04452499	0	0
CDH7	0	0	0	0	0	0	0	0	0	0	0	0	0	0	0
CDH8	0	0	0	0	3.760660301	0.739364317	0	0	0	0	0	0	0	0	0
CDH9	0	0	0	0	0	0	0	0	0	0	0	0	0	0	0
CDH10	2.634114809	4.706086705	3.468288609	8.739271866	0	0	0	0	0	0	0	0	0	0	0
CDH11	3.670003402	2.926548566	1.246284363	5.128266455	0.230919828	0.391530819	0	1.896030235	0	3.678406873	9.263986323	9.53705449	4.853776998	0	0
CDH12	0	0	0	0	0	0	0	0	0	0	2.088460781	0	0	0	0
CDH13	0	0	6.169183353	8.803493106	0	2.319998046	1.797460761	0	0	0	0	0	0	0	0.275892623
CDH18	0	0	3.284761507	4.614918395	0	0	0	0	1.605891796	0	0	0	0	0	0
CDH19	0	0	0.227049578	0	2.587008543	5.197361259	4.693031474	5.93582532	0	0	0	0	0	0	0
CDH20	10.96147098	10.91329649	8.627957843	9.45805911	9.935550579	9.698508874	9.432792868	6.476738977	0	0	0	0	0	0	0
CDH22	0	0	0	0	0	0	0	0	0	0	0	0	0	0	0
CDH24	0	0	0	0	0	0	0	0	0	0	0	0	0	0	0

Fig 6B. dN and dN/dS values between H. sapiens vs. non-human primates used for the correlation analysis shown in Fig 6B

	Pan troglodyte	*Pan paniscus*	*Gorilla gorilla*	*Pongo abelii*	*Nomascus leucogenys*	*Macaca mulatta*	*Macaca fascicularis*	*Papio anubis*	*Chlorocebus sabaeus*	*Rhinopithecus roxellana*	*Saimiri boliviensis*	*Callithrix jacchus*	*Tarsius syrichta (Carlito*	*Otolemur garnettii*	*Microcebus murinus*	*Mus musculus*
Myr ago	7.0	7.0	8.1	14.5	17.6	27.5	27.5	27.5	27.5	27.5	39.9	39.9	49.6	57.2	57.2	81.0
**dN**																
CDH1	0.00128176	0.001853673		0.008154438	0.006131397	0.017933923	0.017939818	0.018311104	0.014951574	0.01248539	0.042700182	0.033657195	0.050534672		0.069033226	0.089551781
CDH2	0.001293244	0.001293244	0.000646204	0.003002466	0.000646831	0.001822592	0.001822592	0.001822592	0.001822731	0.001822591	0.002999181	0.003527741	0.007604412	0.005124973	0.005839584	0.007117592
CDH3	0.001935368	0.0040134	0.014403675	0.005130405	0.016929078	0.018076609	0.018226179	0.017230531	0.018374027	0.015951233	0.027878171	0.028087645	0.04190465	0.043623954	0.040546234	0.069061274
CDH4	0.001297859	0.001297859	0.000648298	0.00379003	0.007722983	0.002598252	0.003250987	0.003252044	0.002779715	0.003137207	0.006180154	0.006835854	0.015042159	0.016068821	0.018613678	0.026476004
CDH5	0.001331116	0.001868494	0.003072702	0.006166676	0.008123064	0.006168603	0.00616918	0.006841641	0.004956119	0.005625995	0.041264576	0.031773309	0.090110905	0.076852555	0.072273452	0.141989547
CDH6	0.00133556	0.00066756	0.00186971	0.00521825	0.00374394	0.00186940	0.00186940	0.00186867	0.00307455	0.00374361	0.00576042	0.00307416	0.00455762	0.02044913	0.01130829	0.01081731
CDH7	0.0000000000	0.0011950721	0.0013275813	0.0006633500	0.0013271408	0.0013284631	0.0013284631	0.0013284631	0.0013284631	0.0013280220	0.0025252760	0.0019920345	0.0047895282	0.0054673175	0.0037241494	0.0100886334
CDH8	0.0000000000	0.0000000000	0.0000000000	0.0006631301	0.0011955192	0.0000000000	0.0000000000	0.0000000000	0.0000000000	0.0006635701	0.0006633500	0.0013284631	0.0019940206	0.0019914583	0.0029898299	0.00696671
CDH9	0.00000000	0.00066181	0.00000000	0.00000000	0.00052394	0.00209698	0.00157275	0.00209753	0.00289867	0.00303990	0.00556661	0.00490008	0.00935932	0.03955587	0.00849096	0.02996500
CDH10	0	0	0	0	0.000534055	0.001198225	0.001198225	0.000533302	0.000533302	0.000533728	0.002801932	0.000533301	0.001329346	0.001333034	0.00136334	0.007283212
CDH11	0.001185056	0.000528508	0.000528508	0.000528508	0.001714158	0.002499816	0.002499816	0.002499816	0.002499537	0.002498889	0.005943169	0.005942079	0.002536876	0.004224064	0.003069074	0.009974199
CDH12	0.00066291	0.000859845	0.003090008	0.002656931	0.001991373	0.003050854	0.003050854	0.002521004	0.00318717	0.002520622	0.006785983	0.008787516	0.009639241	0.008649747	0.00598135	0.018305513
CDH13	0.0000000000	0.0000000000	0.0029220042	0.0035431847	0.0035483350	0.0021525560	0.0029200480	0.0027734228	0.0021525560	0.0036902975	0.0040663368	0.0102037062	0.0116279267	0.0155121963	0.0154750020	0.0223358139
CDH18	0.0012041253	0.0005365552	0.0012046204	0.0032146882	0.0032155846	0.0056382125	0.0056377805	0.0056382125	0.0056377805	0.0063101624	0.0063169131	0.0063139171	0.0152519767	0.0156561859	0.0133883510	0.0186845500
CDH19	0.00385922	0.00438781	0.00424256	0.01280547	0.01324235	0.01531816	0.01478461	0.01478132	0.01632016	0.01682055	0.03252465	0.02535125	0.06003488	0.09036372	0.05542422	0.13368967
CDH20	0.000529628	0.001187902	0	0.001717556	0.00131579	0.003827367	0.003827367	0.003297078	0.003166211	0.004359486	0.00237724	0.00225003	0.004324673	0.008147339	0.005823181	0.010880221
CDH22	0.001304632	0.001304632	0.001297859	0.002613531	0.004557323	0.004079685	0.004079685	0.005942062	0.002895992	0.002896413	0.006042875	0.003666273		0.010435929	0.008597661	0.019658974
CDH24	0.006220924	0.004964157	0.004396641	0.004275448	0.006883218	0.008863837	0.008863837	0.008171604	0.007479768	0.009554988	0.012591204	0.013609651		0.019430248	0.019758407	0.034352243
**dNdS**																
CDH1	0.047784346	0.060059282	0	0.125939941	0.162310272	0.217933952	0.218187579	0.238977695	0.200802324	0.158037129	0.273957006	0.214530097	0.165070074		0.212828957	0.143113638
CDH2	0.105265695	0.105301138	0.051303438	0.140112367	0.02334438	0.04695303	0.04999448	0.04654641	0.041855727	0.051422646	0.036394112	0.054903261	0.038687865	0.026837881	0.024605432	0.013496884
CDH3	0.134614842	0.283663207	1.104514548	0.105962621	0.298373929	0.210829535	0.20895365	0.176228519	0.20841695	0.182658705	0.213529625	0.208171021	0.144990091	0.153297528	0.121453861	0.127576464
CDH4	0.063758831	0.056221413	0.022044563	0.053938468	0.12149342	0.027850448	0.035695482	0.03020459	0.026856609	0.029670775	0.032414106	0.037698836	0.038994918	0.044748336	0.050984373	0.042210515
CDH5	0.096129941	0.123358448	0.163986549	0.140144988	0.12766785	0.065409202	0.065438065	0.068259789	0.056962966	0.063363351	0.346371117	0.254916956	0.341258447	0.254458756	0.253396832	0.313188626
CDH6	0.104033694	0.057834965	0.097378552	0.214131579	0.162405964	0.04226667	0.045106071	0.047937537	0.0763427	0.098849514	0.04763341	0.042767582	0.025346361	0.073439595	0.040438732	0.028727838
CDH7	0	0.116879883	0.08082276	0.01885833	0.031192647	0.017144384	0.016330668	0.017142319	0.016256456	0.019080048	0.019314881	0.016466769	0.022211524	0.010445837	0.014185786	0.020786268
CDH8	0	0	0	0.027232403	0.034554722	0	0	0	0	0.011659425	0.007269505	0.013273578	0.010350102	0.008728019	0.015969953	0.020948744
CDH9	0	0.07225532	0	0	0.014103548	0.027369636	0.020186465	0.032285516	0.04456919	0.044989534	0.047492138	0.037419854	0.048046934	0.117548952	0.035683363	0.058378581
CDH10	0	0	0	0	0.014468648	0.020576739	0.020576739	0.007850223	0.008060659	0.008959322	0.030340103	0.005205746	0.006650226	0.003845213	0.006027232	0.014771751
CDH11	0.086202996	0.047498032	0.059771197	0.015304689	0.060763512	0.053258406	0.048896745	0.044089616	0.047212	0.051970261	0.057229213	0.064880001	0.011806139	0.015904247	0.012276287	0.025219826
CDH12	0.065416398	0.058199127	0.171470682	0.083583436	0.059492665	0.0536793	0.0536793	0.04234212	0.048949613	0.04542446	0.053910557	0.072204488	0.043919392	0.029136045	0.029651798	0.045836316
CDH13	0	0	0.174059901	0.079221681	0.051600294	0.034178986	0.051676191	0.045212562	0.034395151	0.052236076	0.034750707	0.091608038	0.041743097	0.062537615	0.059806916	0.050122026
CDH18	0.077057656	0.034336699	0.079230549	0.110834782	0.080936607	0.083253483	0.088430246	0.079833479	0.082386468	0.093124495	0.054670179	0.056831485	0.082198593	0.055132972	0.077824439	0.041640729
CDH19	0.420222981	0.670740411	0.35679768	0.565808976	0.622184539	0.286201516	0.262414882	0.275654604	0.252509374	0.271534703	0.27721496	0.236971113	0.254646085	0.24789367	0.245997838	0.239116903
CDH20	0.032083267	0.066660106	0	0.047795776	0.02710841	0.057753036	0.059018972	0.050311597	0.047694091	0.068434006	0.025551924	0.020182166	0.019280728	0.034687705	0.027426898	0.02621629
CDH22	0.09065961	0.099549984	0.033449458	0.059752843	0.140707198	0.064580814	0.064672276	0.087386293	0.044995936	0.052845641	0.064680414	0.038238273		0.045432663	0.056117759	0.050033816
CDH24	0.504632405	0.283467338	0.174356952	0.092789835	0.231386039	0.147231823	0.143918808	0.126762513	0.117426735	0.154615223	0.12472878	0.137027123		0.069799457	0.08763669	0.089199725

S5 File. Fig 6C, D. Plotted data. Spearman's correlation:dN, dN/dS (human to mouse), and LOEUF values vs. cadherins scRNA expression in 386 cells from mouse cerebral cortex. (2-tailed p < 0.05*, p < 0.01)**

					H. sapiens vs. M. musculus								*H. sapiens vs. M. musculus*		
**Sample #**	**Cell type**				**dN**	**dNdS**	**LOEUF**	**Sample #**	**Cell type**				**dN**	**dNdS**	**LOEUF**
1	158_L2/3 IT AI	NEURONAL	GLU	ISOCORTEX	-.594**	-.616**	-0.249	41	240_L5 PT CTX	NEURONAL	GLU	ISOCORTEX	-.669**	-.634**	-0.365
2	159_L2/3 IT AI	NEURONAL	GLU	ISOCORTEX	-.614**	-.561*	-0.21	42	241_L5 PT CTX	NEURONAL	GLU	ISOCORTEX	-.636**	-.585*	-.491*
3	162_L2/3 IT CTX	NEURONAL	GLU	ISOCORTEX	-0.467	-.520*	-0.356	43	242_L5 PT CTX	NEURONAL	GLU	ISOCORTEX	-.607**	-.601**	-0.403
4	163_L2/3 IT CTX	NEURONAL	GLU	ISOCORTEX	-.756**	-.750**	-0.461	44	243_L5 PT CTX	NEURONAL	GLU	ISOCORTEX	-.830**	-.771**	-.475*
5	164_L2/3 IT CTX	NEURONAL	GLU	ISOCORTEX	-.492*	-.541*	-0.382	45	244_L5 PT CTX	NEURONAL	GLU	ISOCORTEX	-.802**	-.733**	-0.468
6	165_L2/3 IT CTX	NEURONAL	GLU	ISOCORTEX	-.596**	-.572*	-.506*	46	245_L5 PT CTX	NEURONAL	GLU	ISOCORTEX	-.764**	-.736**	-.476*
7	166_L2/3 IT CTX	NEURONAL	GLU	ISOCORTEX	-.775**	-.733**	-.539*	47	246_L5 PT CTX	NEURONAL	GLU	ISOCORTEX	-.669**	-.658**	-0.235
8	167_L2/3 IT CTX	NEURONAL	GLU	ISOCORTEX	-.687**	-.657**	-0.445	48	247_L5 PT CTX	NEURONAL	GLU	ISOCORTEX	-.716**	-.669**	-0.204
9	168_L2/3 IT CTX	NEURONAL	GLU	ISOCORTEX	-.854**	-.801**	-0.331	49	248_L5 PT CTX	NEURONAL	GLU	ISOCORTEX	-.851**	-.776**	-0.432
10	169_L2/3 IT CTX	NEURONAL	GLU	ISOCORTEX	-.794**	-.746**	-.500*	50	249_L5 PT CTX	NEURONAL	GLU	ISOCORTEX	-.789**	-.673**	-.564*
11	170_L2/3 IT CTX	NEURONAL	GLU	ISOCORTEX	-.758**	-.727**	-0.441	51	250_L5 PT CTX	NEURONAL	GLU	ISOCORTEX	-.771**	-.666**	-.489*
12	171_L2/3 IT CTX	NEURONAL	GLU	ISOCORTEX	-.684**	-.669**	-.565*	52	251_L5 PT CTX	NEURONAL	GLU	ISOCORTEX	-.641**	-.652**	-.483*
13	178_L4 IT CTX	NEURONAL	GLU	ISOCORTEX	-.729**	-.654**	-0.37	53	252_L5 PT CTX	NEURONAL	GLU	ISOCORTEX	-.769**	-.744**	-.607**
14	179_L4 IT CTX	NEURONAL	GLU	ISOCORTEX	-.849**	-.764**	-0.344	54	253_L5 PT CTX	NEURONAL	GLU	ISOCORTEX	-.741**	-.733**	-.611**
15	180_L4 IT CTX	NEURONAL	GLU	ISOCORTEX	-.789**	-.690**	-0.39	55	254_L5 PT CTX	NEURONAL	GLU	ISOCORTEX	-.796**	-.760**	-.572*
16	181_L4 IT CTX	NEURONAL	GLU	ISOCORTEX	-.731**	-.640**	-0.337	56	255_L5 PT CTX	NEURONAL	GLU	ISOCORTEX	-.696**	-.683**	-.601**
17	261_L4 RSP-ACA	NEURONAL	GLU	ISOCORTEX	-.735**	-.644**	-0.429	57	256_L5 PT CTX	NEURONAL	GLU	ISOCORTEX	-.764**	-.687**	-.641**
18	262_L4 RSP-ACA	NEURONAL	GLU	ISOCORTEX	-.724**	-.627**	-0.406	58	257_L5 PT CTX	NEURONAL	GLU	ISOCORTEX	-.700**	-.643**	-.619**
19	182_L4/5 IT CTX	NEURONAL	GLU	ISOCORTEX	-.775**	-.671**	-0.422	59	258_L5 PT CTX	NEURONAL	GLU	ISOCORTEX	-.837**	-.735**	-.551*
20	183_L4/5 IT CTX	NEURONAL	GLU	ISOCORTEX	-.727**	-.581*	-0.388	60	259_L5 PT CTX	NEURONAL	GLU	ISOCORTEX	-.791**	-.723**	-.559*
21	184_L4/5 IT CTX	NEURONAL	GLU	ISOCORTEX	-.828**	-.769**	-.503*	61	260_L5 PT CTX	NEURONAL	GLU	ISOCORTEX	-.856**	-.745**	-.570*
22	185_L4/5 IT CTX	NEURONAL	GLU	ISOCORTEX	-.765**	-.742**	-.521*	62	263_L5 PPP	NEURONAL	GLU	ISOCORTEX	-.536*	-.634**	-0.065
23	186_L4/5 IT CTX	NEURONAL	GLU	ISOCORTEX	-.682**	-.604**	-0.41	63	203_L5/6 IT CTX	NEURONAL	GLU	ISOCORTEX	-.744**	-.708**	-0.332
24	187_L4/5 IT CTX	NEURONAL	GLU	ISOCORTEX	-.864**	-.828**	-0.339	64	204_L5/6 IT CTX	NEURONAL	GLU	ISOCORTEX	-.661**	-.600**	-0.333
25	188_L4/5 IT CTX	NEURONAL	GLU	ISOCORTEX	-.875**	-.788**	-0.359	65	205_L5/6 IT CTX	NEURONAL	GLU	ISOCORTEX	-.547*	-.537*	-0.287
26	189_L4/5 IT CTX	NEURONAL	GLU	ISOCORTEX	-.770**	-.676**	-0.353	66	206_L5/6 IT CTX	NEURONAL	GLU	ISOCORTEX	-.626**	-.623**	-0.322
27	190_L4/5 IT CTX	NEURONAL	GLU	ISOCORTEX	-.740**	-.623**	-0.26	67	207_L5/6 IT CTX	NEURONAL	GLU	ISOCORTEX	-.550*	-.513*	-0.332
28	191_L4/5 IT CTX	NEURONAL	GLU	ISOCORTEX	-.811**	-.739**	-0.442	68	208_L5/6 IT CTX	NEURONAL	GLU	ISOCORTEX	-.479*	-.476*	-0.241
29	192_L4/5 IT CTX	NEURONAL	GLU	ISOCORTEX	-.724**	-.645**	-0.355	69	209_L5/6 IT CTX	NEURONAL	GLU	ISOCORTEX	-.503*	-0.458	-0.275
30	193_L4/5 IT CTX	NEURONAL	GLU	ISOCORTEX	-.745**	-.654**	-0.362	70	210_L5/6 IT TPE-ENT	NEURONAL	GLU	ISOCORTEX	-.624**	-.610**	-0.177
31	194_L5 IT RSP-ACA	NEURONAL	GLU	ISOCORTEX	-.759**	-.679**	-0.339	71	211_L5/6 IT TPE-ENT	NEURONAL	GLU	ISOCORTEX	-.600**	-.607**	-.498*
32	195_L5 IT RSP-ACA	NEURONAL	GLU	ISOCORTEX	-.682**	-.607**	-0.229	72	212_L5/6 IT TPE-ENT	NEURONAL	GLU	ISOCORTEX	-.624**	-.554*	-0.455
33	196_L5 IT CTX	NEURONAL	GLU	ISOCORTEX	-.722**	-.614**	-0.259	73	213_L5/6 IT TPE-ENT	NEURONAL	GLU	ISOCORTEX	-.638**	-.583*	-0.419
34	197_L5 IT CTX	NEURONAL	GLU	ISOCORTEX	-.762**	-.667**	-0.424	74	214_L5/6 IT PFC	NEURONAL	GLU	ISOCORTEX	-.579*	-.559*	-0.447
35	198_L5 IT CTX	NEURONAL	GLU	ISOCORTEX	-.675**	-.595**	-0.335	75	215_L5/6 IT TPE-ENT	NEURONAL	GLU	ISOCORTEX	-.713**	-.619**	-0.332
36	199_L5 IT CTX	NEURONAL	GLU	ISOCORTEX	-.718**	-.654**	-0.336	76	216_L5/6 IT TPE-ENT	NEURONAL	GLU	ISOCORTEX	-.557*	-.486*	-0.37
37	200_L5 IT CTX	NEURONAL	GLU	ISOCORTEX	-.674**	-.630**	-0.262	77	264_L5/6 NP CTX	NEURONAL	GLU	ISOCORTEX	-.651**	-.572*	-0.242
38	201_L5 IT CTX	NEURONAL	GLU	ISOCORTEX	-.678**	-.636**	-0.226	78	265_L5/6 NP CTX	NEURONAL	GLU	ISOCORTEX	-.597**	-.485*	-0.344
39	202_L5 IT CTX	NEURONAL	GLU	ISOCORTEX	-.652**	-.605**	-0.377	79	266_L5/6 NP CTX	NEURONAL	GLU	ISOCORTEX	-.633**	-.579*	-.538*
40	239_L5 PT CTX	NEURONAL	GLU	ISOCORTEX	-.724**	-.669**	-0.365	80	267_L5/6 NP CTX	NEURONAL	GLU	ISOCORTEX	-.600**	-.503*	-0.419

S5 File. Fig 6C, D. Cont.

					H. sapiens vs. M. musculus								*H. sapiens vs. M. musculus*		
Sample #	Cell type				dN	dNdS	LOEUF	Sample #	Cell type				dN	dNdS	LOEUF
81	268_L5/6 NP CTX	NEURONAL	GLU	ISOCORTEX	-.495*	-0.386	-0.405	121	305_L6b CTX	NEURONAL	GLU	ISOCORTEX	-.504*	-.508*	-0.098
82	269_L5/6 NP CTX	NEURONAL	GLU	ISOCORTEX	-.623**	-.537*	-0.319	122	306_L6b CTX	NEURONAL	GLU	ISOCORTEX	-0.463	-0.457	-0.154
83	270_L5/6 NP CT CTX	NEURONAL	GLU	ISOCORTEX	-.592**	-.503*	-0.403	123	307_L6b CTX	NEURONAL	GLU	ISOCORTEX	-0.452	-0.445	-0.106
84	217_L6 IT CTX	NEURONAL	GLU	ISOCORTEX	-.670**	-.593**	-0.445	124	311_L6b CTX	NEURONAL	GLU	ISOCORTEX	-.557*	-.506*	-0.282
85	218_L6 IT CTX	NEURONAL	GLU	ISOCORTEX	-.772**	-.694**	-0.313	125	312_L6b CTX	NEURONAL	GLU	ISOCORTEX	-.528*	-.509*	-0.286
86	219_L6 IT CTX	NEURONAL	GLU	ISOCORTEX	-.640**	-.541*	-0.366	126	313_L6b CTX	NEURONAL	GLU	ISOCORTEX	-.550*	-.486*	-0.314
87	220_L6 IT CTX	NEURONAL	GLU	ISOCORTEX	-.619**	-.506*	-0.425	127	314_L6b CTX	NEURONAL	GLU	ISOCORTEX	-0.404	-0.386	-0.195
88	221_L6 IT CTX	NEURONAL	GLU	ISOCORTEX	-.515*	-0.443	-0.419	128	315_L6b CTX	NEURONAL	GLU	ISOCORTEX	-.541*	-.491*	-0.275
89	222_L6 IT CTX	NEURONAL	GLU	ISOCORTEX	-.535*	-0.444	-0.431	129	316_L6b CTX	NEURONAL	GLU	ISOCORTEX	-.510*	-.484*	-0.303
90	223_L6 IT CTX	NEURONAL	GLU	ISOCORTEX	-.502*	-0.450	-0.436	130	317_L6b CTX	NEURONAL	GLU	ISOCORTEX	-0.412	-0.383	-0.29
91	224_L6 IT CTX	NEURONAL	GLU	ISOCORTEX	-0.457	-0.433	-0.427	131	124_L2 IT APr	NEURONAL	GLU	HPF	-.682**	-.661**	-0.277
92	225_L6 IT CTX	NEURONAL	GLU	ISOCORTEX	-0.458	-0.451	-.528*	132	125_L2 IT APr	NEURONAL	GLU	HPF	-.730**	-.718**	-0.116
93	226_L6 IT CTX	NEURONAL	GLU	ISOCORTEX	-0.385	-0.374	-0.438	133	126_L2 IT APr	NEURONAL	GLU	HPF	-.646**	-.600**	-0.178
94	227_L6 IT CTX	NEURONAL	GLU	ISOCORTEX	-.498*	-0.437	-0.38	134	127_L2 IT APr	NEURONAL	GLU	HPF	-.547*	-.518*	-0.233
95	228_L6 IT CTX	NEURONAL	GLU	ISOCORTEX	-0.445	-0.403	-0.375	135	128_L2 IT APr	NEURONAL	GLU	HPF	-.702**	-.647**	-0.283
96	229_L6 IT CTX	NEURONAL	GLU	ISOCORTEX	-0.438	-0.431	-0.333	136	132_L2 IT RSPv-POST-	NEURONAL	GLU	HPF	-0.464	-0.405	-0.318
97	230_L6 IT CTX	NEURONAL	GLU	ISOCORTEX	-0.445	-0.409	-0.43	137	133_L2 IT RSPv-POST-	NEURONAL	GLU	HPF	-.689**	-.607**	-0.319
98	231_L6 IT CTX	NEURONAL	GLU	ISOCORTEX	-0.370	-0.344	-0.372	138	134_L2 IT RSP-ACA	NEURONAL	GLU	HPF	-.783**	-.726**	-0.297
99	232_L6 IT CTX	NEURONAL	GLU	ISOCORTEX	-0.266	-0.255	-0.236	139	146_L2 IT ENTm	NEURONAL	GLU	HPF	-.609**	-.565*	-0.462
100	275_NP PPP	NEURONAL	GLU	ISOCORTEX	-0.384	-0.381	-0.262	140	147_L2 IT ENTm	NEURONAL	GLU	HPF	-0.464	-0.464	-.476*
101	276_NP PPP	NEURONAL	GLU	ISOCORTEX	-0.406	-0.453	-0.237	141	148_L2 IT ENTm	NEURONAL	GLU	HPF	-.536*	-.538*	-0.468
102	277_NP PPP	NEURONAL	GLU	ISOCORTEX	-0.344	-0.370	-0.358	142	149_L2 IT ENTm	NEURONAL	GLU	HPF	-0.424	-0.372	-0.278
103	278_L6 CT CTX	NEURONAL	GLU	Isocortex	-.650**	-.578*	-0.354	143	150_L2 IT ENTm	NEURONAL	GLU	HPF	-.623**	-.579*	-0.392
104	279_L6 CT CTX	NEURONAL	GLU	Isocortex	-0.466	-0.428	-0.237	144	151_L2 IT ENTl	NEURONAL	GLU	HPF	-.583*	-.570*	-0.182
105	280_L6 CT CTX	NEURONAL	GLU	Isocortex	-.555*	-.510*	-0.337	145	152_L2/3 IT ENTl	NEURONAL	GLU	HPF	-.757**	-.700**	-0.369
106	281_L6 CT CTX	NEURONAL	GLU	Isocortex	-0.460	-0.400	-0.414	146	153_L2/3 IT ENTl	NEURONAL	GLU	HPF	-.754**	-.728**	-0.422
107	282_L6 CT CTX	NEURONAL	GLU	Isocortex	-.499*	-.478*	-0.376	147	154_L2/3 IT ENTl	NEURONAL	GLU	HPF	-.775**	-.752**	-.496*
108	283_L6 CT CTX	NEURONAL	GLU	Isocortex	-0.450	-0.417	-0.361	148	155_L2/3 IT ENTl	NEURONAL	GLU	HPF	-.626**	-.567*	-.518*
109	284_L6 CT CTX	NEURONAL	GLU	Isocortex	-0.448	-0.409	-0.41	149	156_L2/3 IT ENTl	NEURONAL	GLU	HPF	-.554*	-.521*	-0.444
110	285_L6 CT CTX	NEURONAL	GLU	Isocortex	-0.458	-0.451	-0.452	150	157_L2/3 IT ENTl	NEURONAL	GLU	HPF	-.705**	-.633**	-0.378
111	286_L6 CT CTX	NEURONAL	GLU	Isocortex	-0.465	-0.374	-0.457	151	160_L2/3 IT ENTl	NEURONAL	GLU	HPF	-.592**	-.552*	-0.459
112	287_L6 CT CTX	NEURONAL	GLU	Isocortex	-0.330	-0.264	-0.13	152	161_L2/3 IT ENTl	NEURONAL	GLU	HPF	-.581*	-.540*	-0.453
113	288_L6 CT CTX	NEURONAL	GLU	Isocortex	-.483*	-0.390	-0.448	153	172_L2/3 IT ProS	NEURONAL	GLU	HPF	-.733**	-.639**	-.616**
114	289_L6 CT CTX	NEURONAL	GLU	Isocortex	-0.421	-0.383	-0.374	154	129_L2/3 IT POST-PRE	NEURONAL	GLU	HPF	-.696**	-.633**	-0.32
115	290_L6 CT CTX	NEURONAL	GLU	Isocortex	-.499*	-0.438	-0.46	155	130_L2/3 IT POST-PRE	NEURONAL	GLU	HPF	-.736**	-.658**	-0.422
116	291_L6 CT CTX	NEURONAL	GLU	Isocortex	-.524*	-.471*	-0.379	156	131_L2/3 IT POST-PRE	NEURONAL	GLU	HPF	-.722**	-.656**	-0.334
117	292_L6 CT CTX	NEURONAL	GLU	Isocortex	-.569*	-.517*	-0.313	157	141_L2/3 IT PAR	NEURONAL	GLU	HPF	-.725**	-.654**	-0.392
118	293_L6 CT CTX	NEURONAL	GLU	Isocortex	-.544*	-.489*	-0.312	158	142_L2/3 IT PAR	NEURONAL	GLU	HPF	-.766**	-.658**	-0.392
119	303_L6b CTX	NEURONAL	GLU	ISOCORTEX	-.529*	-.513*	-0.355	159	143_L2/3 IT PAR	NEURONAL	GLU	HPF	-.751**	-.667**	-0.449
120	304_L6b CTX	NEURONAL	GLU	ISOCORTEX	-0.461	-0.435	-0.364	160	144_L2/3 IT PAR	NEURONAL	GLU	HPF	-.698**	-.603**	-0.365

S5 File. Fig 6C, D. Cont.					*H. sapiens vs. M. musculus*								*H. sapiens vs. M. musculus*		
**Sample #**	**Cell type**				**dN**	**dNdS**	**LOEUF**	**Sample #**	**Cell type**				**dN**	**dNdS**	**LOEUF**
161	145_L2/3 IT PAR	NEURONAL	GLU	HPF	-.781**	-.713**	-.480*	201	327_ProS	NEURONAL	GLU	HPF	-0.462	-0.461	-.520*
162	173_L2/3 IT ProS	NEURONAL	GLU	HPF	-.555*	-.497*	-.628**	202	328_ProS	NEURONAL	GLU	HPF	-0.460	-0.420	-.498*
163	135_L3 IT ENTm	NEURONAL	GLU	HPF	-.684**	-.640**	-0.254	203	329_CA1-ProS	NEURONAL	GLU	HPF	-.706**	-.637**	-.515*
164	136_L3 IT ENTm	NEURONAL	GLU	HPF	-.670**	-.612**	-0.309	204	330_CA1-ProS	NEURONAL	GLU	HPF	-.656**	-.629**	-.584*
165	137_L3 IT ENTm	NEURONAL	GLU	HPF	-.733**	-.707**	-0.379	205	331_CA1-ProS	NEURONAL	GLU	HPF	-.594**	-.551*	-0.343
166	138_L3 IT ENTm	NEURONAL	GLU	HPF	-.702**	-.665**	-0.336	206	332_CA1-ProS	NEURONAL	GLU	HPF	-.681**	-.635**	-.508*
167	139_L3 IT ENTl	NEURONAL	GLU	HPF	-.701**	-.633**	-0.321	207	333_CA1-ProS	NEURONAL	GLU	HPF	-.685**	-.607**	-.613**
168	140_L3 IT ENTl	NEURONAL	GLU	HPF	-.768**	-.760**	-0.345	208	334_CA1-ve	NEURONAL	GLU	HPF	-.533*	-0.460	-.572*
169	174_IT HATA	NEURONAL	GLU	HPF	-0.454	-.469*	-0.267	209	335_CA1-ve	NEURONAL	GLU	HPF	-.666**	-.574*	-0.41
170	175_IT HATA	NEURONAL	GLU	HPF	-.520*	-.508*	-.554*	210	336_CA1-ve	NEURONAL	GLU	HPF	-.661**	-.590**	-0.354
171	176_IT HATA	NEURONAL	GLU	HPF	-.515*	-.539*	-.612**	211	337_CA1	NEURONAL	GLU	HPF	-.727**	-.707**	-0.416
172	177_IT HATA	NEURONAL	GLU	HPF	-.693**	-.694**	-.678**	212	338_CA1	NEURONAL	GLU	HPF	-.764**	-.682**	-0.402
173	233_L6 IT ENTl	NEURONAL	GLU	HPF	-0.365	-0.420	-0.458	213	339_CA1	NEURONAL	GLU	HPF	-.628**	-.562*	-0.307
174	234_L6 IT ENTl	NEURONAL	GLU	HPF	-.488*	-.508*	-.575*	214	340_CA1	NEURONAL	GLU	HPF	-.665**	-.590**	-0.412
175	235_L6 IT ENTl	NEURONAL	GLU	HPF	-0.431	-0.432	-.502*	215	341_CA1	NEURONAL	GLU	HPF	-.674**	-.606**	-0.422
176	271_NP SUB	NEURONAL	GLU	HPF	-.503*	-0.376	-0.426	216	342_CA1	NEURONAL	GLU	HPF	-.643**	-.590**	-.508*
177	272_NP SUB	NEURONAL	GLU	HPF	-.511*	-0.396	-0.399	217	343_CA1	NEURONAL	GLU	HPF	-.657**	-.638**	-0.432
178	273_NP SUB	NEURONAL	GLU	HPF	-.726**	-.630**	-0.363	218	344_CA1	NEURONAL	GLU	HPF	-.610**	-.594**	-0.343
179	274_NP SUB	NEURONAL	GLU	HPF	-.642**	-.597**	-0.34	219	345_CA1	NEURONAL	GLU	HPF	-.644**	-.607**	-0.394
180	294_CT SUB	NEURONAL	GLU	HPF	-0.385	-0.435	-0.241	220	346_CA1-do	NEURONAL	GLU	HPF	-.735**	-.698**	-0.451
181	295_CT SUB	NEURONAL	GLU	HPF	-.481*	-.483*	-0.421	221	347_CA1-do	NEURONAL	GLU	HPF	-.735**	-.682**	-0.397
182	296_CT SUB	NEURONAL	GLU	HPF	-0.396	-0.407	-0.416	222	348_CA1-do	NEURONAL	GLU	HPF	-.723**	-.632**	-0.378
183	297_CT SUB	NEURONAL	GLU	HPF	-0.426	-0.394	-0.397	223	349_Mossy	NEURONAL	GLU	HPF	-.532*	-0.445	-0.439
184	298_L6 CT ENT	NEURONAL	GLU	HPF	-0.269	-0.296	-0.287	224	350_Mossy	NEURONAL	GLU	HPF	-.525*	-.497*	-.568*
185	299_L6 CT ENT	NEURONAL	GLU	HPF	-0.364	-0.392	-0.291	225	351_CA3-ve	NEURONAL	GLU	HPF	-.582*	-.515*	-0.266
186	300_L6b ENT	NEURONAL	GLU	HPF	-.561*	-.562*	-0.241	226	352_CA3-ve	NEURONAL	GLU	HPF	-.536*	-.487*	-0.343
187	301_L6b ENT	NEURONAL	GLU	HPF	-.719**	-.659**	-0.255	227	353_CA3-ve	NEURONAL	GLU	HPF	-.527*	-.489*	-0.327
188	302_L6b ENT	NEURONAL	GLU	HPF	-.546*	-.536*	-0.295	228	354_CA3-ve	NEURONAL	GLU	HPF	-.524*	-0.453	-0.433
189	308_L6b RHP	NEURONAL	GLU	HPF	-.495*	-.513*	-0.342	229	355_CA3-ve	NEURONAL	GLU	HPF	-.674**	-.590**	-0.439
190	309_L6b RHP	NEURONAL	GLU	HPF	-.536*	-.525*	-0.395	230	356_CA3-do	NEURONAL	GLU	HPF	-.496*	-0.427	-0.275
191	310_L6b RHP	NEURONAL	GLU	HPF	-0.452	-.508*	-0.276	231	357_CA3-do	NEURONAL	GLU	HPF	-.496*	-0.427	-0.275
192	318_SUB	NEURONAL	GLU	HPF	-.879**	-.830**	-0.289	232	358_CA3-do	NEURONAL	GLU	HPF	-.485*	-0.409	-0.272
193	319_SUB	NEURONAL	GLU	HPF	-.852**	-.813**	-0.386	233	359_CA2-IG-FC	NEURONAL	GLU	HPF	-.603**	-.521*	-.545*
194	320_SUB	NEURONAL	GLU	HPF	-.815**	-.803**	-0.365	234	360_CA2-IG-FC	NEURONAL	GLU	HPF	-.749**	-.685**	-0.456
195	321_SUB	NEURONAL	GLU	HPF	-.719**	-.662**	-.673**	235	361_DG	NEURONAL	GLU	HPF	-.515*	-0.434	-0.361
196	322_ProS	NEURONAL	GLU	HPF	-.604**	-.478*	-.483*	236	362_DG	NEURONAL	GLU	HPF	-0.440	-0.313	-.490*
197	323_ProS	NEURONAL	GLU	HPF	-.592**	-.607**	-.576*	237	363_DG	NEURONAL	GLU	HPF	-0.419	-0.274	-.517*
198	324_ProS	NEURONAL	GLU	HPF	-.630**	-.607**	-.656**	238	364_DG	NEURONAL	GLU	HPF	-0.363	-0.234	-0.434
199	325_ProS	NEURONAL	GLU	HPF	-.497*	-0.435	-.531*	239	1_CR	NEURONAL	GLU	Cajal-Retzius	-0.108	-0.227	-0.27
200	326_ProS	NEURONAL	GLU	HPF	-.489*	-0.437	-.476*	240	236_Car3	NEURONAL	GLU	UNIQUE	-.568*	-.501*	-0.453

S5 File. Fig 6C, D. Cont.					*H. sapiens vs. M. musculus*								*H. sapiens vs. M. musculus*		
**Sample #**	**Cell type**				**dN**	**dNdS**	**LOEUF**	**Sample #**	**Cell type**				**dN**	**dNdS**	**LOEUF**
241	237_Car3	NEURONAL	GLU	UNIQUE	-.677**	-.561*	-.584*	281	65_Sst Chodl	NEURONAL	GABA	ISOCORTEX	-0.455	-0.455	-.509*
242	238_Car3	NEURONAL	GLU	UNIQUE	-.631**	-.563*	-.630**	282	66_Sst	NEURONAL	GABA	ISOCORTEX	-0.385	-0.305	-0.42
243	19_Pax6	NEURONAL	GABA	ISOCORTEX	-0.324	-0.406	-0.32	283	67_Sst	NEURONAL	GABA	ISOCORTEX	-0.391	-0.330	-0.419
244	20_Pax6	NEURONAL	GABA	ISOCORTEX	-0.358	-0.431	-0.242	284	68_Sst	NEURONAL	GABA	ISOCORTEX	-0.332	-0.261	-0.442
245	21_Sncg	NEURONAL	GABA	ISOCORTEX	-.723**	-.618**	-0.405	285	69_Sst	NEURONAL	GABA	ISOCORTEX	-0.410	-0.360	-0.389
246	22_Sncg	NEURONAL	GABA	ISOCORTEX	-.652**	-.548*	-0.409	286	70_Sst	NEURONAL	GABA	ISOCORTEX	-.611**	-.601**	-.565*
247	23_Sncg	NEURONAL	GABA	ISOCORTEX	-.567*	-.534*	-0.289	287	71_Sst	NEURONAL	GABA	ISOCORTEX	-.541*	-.595**	-0.384
248	24_Sncg	NEURONAL	GABA	ISOCORTEX	-.606**	-.549*	-.501*	288	72_Sst	NEURONAL	GABA	ISOCORTEX	-0.377	-0.445	-0.434
249	25_Sncg	NEURONAL	GABA	ISOCORTEX	-.560*	-.520*	-0.447	289	73_Sst	NEURONAL	GABA	ISOCORTEX	-.613**	-.633**	-.644**
250	31_Sncg	NEURONAL	GABA	ISOCORTEX	-.649**	-.630**	-.664**	290	74_Sst	NEURONAL	GABA	ISOCORTEX	-.497*	-.475*	-.532*
251	32_Sncg	NEURONAL	GABA	ISOCORTEX	-.738**	-.684**	-.601**	291	75_Sst	NEURONAL	GABA	ISOCORTEX	-.495*	-.510*	-.606**
252	33_Sncg	NEURONAL	GABA	ISOCORTEX	-.724**	-.661**	-.629**	292	76_Sst	NEURONAL	GABA	ISOCORTEX	-0.257	-0.266	-0.254
253	34_Sncg	NEURONAL	GABA	ISOCORTEX	-.719**	-.674**	-0.356	293	79_Sst	NEURONAL	GABA	ISOCORTEX	-0.337	-0.316	-0.355
254	35_Sncg	NEURONAL	GABA	ISOCORTEX	-.535*	-.494*	-0.411	294	80_Sst	NEURONAL	GABA	ISOCORTEX	-0.467	-0.451	-0.314
255	36_Sncg	NEURONAL	GABA	ISOCORTEX	-.614**	-.573*	-.560*	295	81_Sst	NEURONAL	GABA	ISOCORTEX	-0.414	-0.421	-0.138
256	37_Sncg	NEURONAL	GABA	ISOCORTEX	-.478*	-.489*	-.470*	296	82_Sst	NEURONAL	GABA	ISOCORTEX	-.515*	-.552*	-0.263
257	38_Sncg	NEURONAL	GABA	ISOCORTEX	-.521*	-.530*	-.654**	297	83_Sst	NEURONAL	GABA	ISOCORTEX	-.476*	-.506*	-0.237
258	39_Sncg	NEURONAL	GABA	ISOCORTEX	-.632**	-.603**	-.689**	298	84_Sst	NEURONAL	GABA	ISOCORTEX	-.482*	-.511*	-0.219
259	40_Vip	NEURONAL	GABA	ISOCORTEX	-.571*	-.478*	-.554*	299	85_Sst	NEURONAL	GABA	ISOCORTEX	-0.366	-0.377	-0.241
260	41_Vip	NEURONAL	GABA	ISOCORTEX	-.532*	-0.455	-.554*	300	86_Sst	NEURONAL	GABA	ISOCORTEX	-0.422	-0.431	-0.38
261	42_Vip	NEURONAL	GABA	ISOCORTEX	-.561*	-.473*	-.515*	301	87_Sst	NEURONAL	GABA	ISOCORTEX	-.579*	-.567*	-0.215
262	43_Vip	NEURONAL	GABA	ISOCORTEX	-.499*	-0.435	-.533*	302	88_Sst	NEURONAL	GABA	ISOCORTEX	-0.407	-0.431	-0.444
263	44_Vip	NEURONAL	GABA	ISOCORTEX	-.793**	-.676**	-.566*	303	89_Sst	NEURONAL	GABA	ISOCORTEX	-0.255	-0.353	-0.404
264	45_Vip	NEURONAL	GABA	ISOCORTEX	-.761**	-.725**	-.517*	304	90_Sst	NEURONAL	GABA	ISOCORTEX	-0.222	-0.342	-0.221
265	46_Vip	NEURONAL	GABA	ISOCORTEX	-.715**	-.708**	-0.295	305	91_Sst	NEURONAL	GABA	ISOCORTEX	-0.385	-.473*	-0.426
266	47_Vip	NEURONAL	GABA	ISOCORTEX	-.494*	-.497*	-0.361	306	92_Sst	NEURONAL	GABA	ISOCORTEX	-0.177	-0.285	-0.294
267	48_Vip	NEURONAL	GABA	ISOCORTEX	-.643**	-.636**	-.569*	307	93_Sst	NEURONAL	GABA	ISOCORTEX	-0.422	-.495*	-.477*
268	49_Vip	NEURONAL	GABA	ISOCORTEX	-.730**	-.786**	-.484*	308	94_Sst	NEURONAL	GABA	ISOCORTEX	-0.300	-0.411	-0.372
269	50_Vip	NEURONAL	GABA	ISOCORTEX	-.864**	-.832**	-.604**	309	95_Sst	NEURONAL	GABA	ISOCORTEX	-0.252	-0.332	-0.411
270	51_Vip	NEURONAL	GABA	ISOCORTEX	-.609**	-.665**	-0.334	310	96_Sst	NEURONAL	GABA	ISOCORTEX	-.537*	-.543*	-.475*
271	52_Vip	NEURONAL	GABA	ISOCORTEX	-.758**	-.721**	-0.457	311	97_Sst	NEURONAL	GABA	ISOCORTEX	-0.422	-0.422	-0.422
272	53_Vip	NEURONAL	GABA	ISOCORTEX	-.744**	-.716**	-0.559	312	98_Sst	NEURONAL	GABA	ISOCORTEX	-0.410	-0.456	-0.459
273	57_Vip Igfbp6	NEURONAL	GABA	ISOCORTEX	-.547*	-.606**	-0.368	313	99_Sst	NEURONAL	GABA	ISOCORTEX	-0.339	-0.401	-0.288
274	58_Vip Igfbp6	NEURONAL	GABA	ISOCORTEX	-.487*	-.599**	-0.396	314	100_Sst	NEURONAL	GABA	ISOCORTEX	-0.439	-0.458	-0.233
275	59_Vip Igfbp6	NEURONAL	GABA	ISOCORTEX	-0.466	-.589*	-0.379	315	101_Sst	NEURONAL	GABA	ISOCORTEX	-0.228	-0.211	-0.036
276	60_Vip Igfbp6	NEURONAL	GABA	ISOCORTEX	-0.367	-.477*	-0.145	316	108_Pvalb	NEURONAL	GABA	ISOCORTEX	-0.314	-0.280	-0.23
277	61_Vip Igfbp6	NEURONAL	GABA	ISOCORTEX	-.628**	-.710**	-0.413	317	109_Pvalb	NEURONAL	GABA	ISOCORTEX	-.501*	-0.421	-0.282
278	62_Vip Igfbp6	NEURONAL	GABA	ISOCORTEX	-.482*	-.590**	-0.24	318	110_Pvalb	NEURONAL	GABA	ISOCORTEX	-.588*	-.529*	-0.316
279	63_Sst Chodl	NEURONAL	GABA	ISOCORTEX	-.517*	-.515*	-.574*	319	111_Pvalb	NEURONAL	GABA	ISOCORTEX	-.473*	-0.407	-0.324
280	64_Sst Chodl	NEURONAL	GABA	ISOCORTEX	-0.458	-0.462	-.485*	320	112_Pvalb	NEURONAL	GABA	ISOCORTEX	-.663**	-.603**	-0.391

S5 File. Fig 6C, D. Cont.					*H. sapiens vs. M. musculus*								*H. sapiens vs. M. musculus*		
**Sample #**	**Cell type**				**dN**	**dNdS**	**LOEUF**	**Sample #**	**Cell type**				**dN**	**dNdS**	**LOEUF**
321	113_Pvalb	NEURONAL	GABA	ISOCORTEX	-.612**	-.600**	-0.178	361	17_Lamp5	NEURONAL	GABA		-0.375	-.510*	-0.306
322	114_Pvalb	NEURONAL	GABA	ISOCORTEX	-.492*	-.526*	-0.18	362	18_Lamp5	NEURONAL	GABA		-0.381	-0.417	-0.387
323	115_Pvalb	NEURONAL	GABA	ISOCORTEX	-0.466	-.528*	-0.265	363	2_Meis2	NEURONAL	GABA		-0.204	-0.174	0.391
324	116_Pvalb	NEURONAL	GABA	ISOCORTEX	-.576*	-.581*	-0.443	364	3_Meis2	NEURONAL	GABA		-0.281	-0.382	-0.115
325	117_Pvalb	NEURONAL	GABA	ISOCORTEX	-.496*	-.488*	-0.256	365	365_Oligo	NON-NEURONAL	OLIGO		-.515*	-.536*	-0.435
326	118_Pvalb	NEURONAL	GABA	ISOCORTEX	-0.310	-0.326	-0.196	366	366_Oligo	NON-NEURONAL	OLIGO		-.485*	-.544*	-.494*
327	119_Pvalb	NEURONAL	GABA	ISOCORTEX	-0.369	-0.334	-0.312	367	367_Oligo	NON-NEURONAL	OLIGO		-0.443	-.492*	-0.283
328	120_Pvalb	NEURONAL	GABA	ISOCORTEX	-0.413	-0.378	-0.244	368	368_Oligo	NON-NEURONAL	OLIGO		-0.427	-0.461	-0.365
329	121_Pvalb	NEURONAL	GABA	ISOCORTEX	-0.316	-0.349	-0.239	369	369_Oligo	NON-NEURONAL	OLIGO		-.530*	-.542*	-.521*
330	4_Meis2 HPF	NEURONAL	GABA	HPF	-.529*	-0.443	-.561*	370	370_Oligo	NON-NEURONAL	OLIGO		-.557*	-.564*	-.587*
331	5_Lamp5 Lhx6	NEURONAL	GABA	HPF	-.500*	-0.406	-.518*	371	371_Oligo	NON-NEURONAL	OLIGO		-.541*	-.589*	-0.427
332	6_Lamp5 Lhx6	NEURONAL	GABA	HPF	-.677**	-.605**	-0.424	372	372_Oligo	NON-NEURONAL	OLIGO		-0.328	-0.380	-0.133
333	7_Lamp5 Lhx6	NEURONAL	GABA	HPF	-.468*	-.495*	-0.315	373	373_Oligo	NON-NEURONAL	OLIGO		-0.307	-0.362	-0.106
334	8_Lamp5 Lhx6	NEURONAL	GABA	HPF	-.500*	-.515*	-0.351	374	374_Oligo	NON-NEURONAL	OLIGO		-0.239	-0.317	0.027
335	9_Lamp5 Lhx6	NEURONAL	GABA	HPF	-0.429	-0.417	-0.358	375	375_Oligo	NON-NEURONAL	OLIGO		-0.075	-0.135	0.185
336	54_Vip HPF	NEURONAL	GABA	HPF	-.779**	-.824**	-.554*	376	376_Astro	NON-NEURONAL	ASTRO		-0.372	-0.432	-0.17
337	55_Vip HPF	NEURONAL	GABA	HPF	-.740**	-.779**	-.553*	377	377_Astro	NON-NEURONAL	ASTRO		-0.351	-0.397	-0.351
338	56_Vip HPF	NEURONAL	GABA	HPF	-.623**	-.705**	-0.437	378	378_Astro	NON-NEURONAL	ASTRO		-0.273	-0.354	-0.054
339	26_Ntng1 HPF	NEURONAL	GABA	HPF	-.547*	-0.461	-.593**	379	379_Endo	NON-NEURONAL	Other		0.397	0.397	0.094
340	27_Ntng1 HPF	NEURONAL	GABA	HPF	-.578*	-.552*	-0.443	380	380_SMC-Peri	NON-NEURONAL	Other		-0.164	-0.117	-0.187
341	28_Ntng1 HPF	NEURONAL	GABA	HPF	-.619**	-.562*	-.543*	381	382_SMC-Peri	NON-NEURONAL	Other		-0.351	-0.397	-0.351
342	29_Ntng1 HPF	NEURONAL	GABA	HPF	-.607**	-.576*	-.607**	382	384_VLMC	NON-NEURONAL	Other		0.397	0.397	0.094
343	30_Ntng1 HPF	NEURONAL	GABA	HPF	-.633**	-.649**	-0.388	383	385_VLMC	NON-NEURONAL	Other		0.128	0.161	-0.202
344	77_Sst HPF	NEURONAL	GABA	HPF	-.655**	-.659**	-0.41	384	386_Micro-PVM	NON-NEURONAL	Other		N/A	N/A	N/A
345	78_Sst HPF	NEURONAL	GABA	HPF	-0.442	-0.442	-.564*	385	387_Micro-PVM	NON-NEURONAL	Other		N/A	N/A	N/A
346	103_Sst HPF	NEURONAL	GABA	HPF	-.526*	-.486*	-.697**	386	388_Micro-PVM	NON-NEURONAL	Other		N/A	N/A	N/A
347	104_Sst HPF	NEURONAL	GABA	HPF	-.617**	-.560*	-0.388								
348	105_Sst HPF	NEURONAL	GABA	HPF	-.509*	-.514*	-0.25								
349	106_Sst HPF	NEURONAL	GABA	HPF	-.539*	-.605**	-0.459								
350	107_Sst HPF	NEURONAL	GABA	HPF	-.654**	-.720**	-0.401								
351	102_Sst HPF	NEURONAL	GABA	HPF	-.495*	-0.420	-0.381								
352	122_Pvalb Vipr2	NEURONAL	GABA		-0.407	-0.433	-0.328								
353	123_Pvalb Vipr2	NEURONAL	GABA		-0.420	-0.444	-0.202								
354	10_Lamp5	NEURONAL	GABA		-.494*	-.558*	-0.442								
355	11_Lamp5	NEURONAL	GABA		-.473*	-.534*	-0.44								
356	12_Lamp5	NEURONAL	GABA		-0.457	-0.452	-0.314								
357	13_Lamp5	NEURONAL	GABA		-0.290	-0.381	-0.422								
358	14_Lamp5	NEURONAL	GABA		-0.245	-0.342	-0.356								
359	15_Lamp5	NEURONAL	GABA		-0.336	-0.416	-0.314								
360	16_Lamp5	NEURONAL	GABA		-0.455	-.544*	-0.288								

S5 File. Fig 6C, D. dN, dN/dS and scRNA expression values in mouse cortex used for correlation analaysis

	Gene	*CDH1*	*CDH2*	*CDH3*	*CDH4*	*CDH5*	*CDH6*	*CDH7*	*CDH8*	*CDH9*	*CDH10*	*CDH11*	*CDH12*	*CDH13*	*CDH18*	*CDH19*	*CDH20*	*CDH22*	*CDH24*
**dN (Human - Mouse)**		0.089552	0.007118	0.069061	0.026476	0.14199	0.01081731	0.0100886334	0.00696671	0.02996500	0.007283	0.009974	0.018306	0.0223358139	0.0186845500	0.13368967	0.01088	0.019659	0.034352
**dNdS (Human - Mouse)**		0.143114	0.013497	0.127576	0.042211	0.313189	0.028727838	0.020786268	0.020948744	0.058378581	0.014772	0.02522	0.045836	0.050122026	0.041640729	0.239116903	0.026216	0.050034	0.0892
**LOEUF score**		0.51	0.34	0.82	0.53	0.66	0.51	0.65	0.37	0.58	0.66	0.25	0.67	0.46	0.74	1.1	0.53	0.78	1.15
**Cell type**	**Cell #**																		
158_L2/3 IT AI	1	0	6.518462	0	2.803684	0	7.3207693	3.6194737	0.9021053	8.883077	8.921538	0.879474	7.828462	0	0	0	3.962632	1.183684	0
159_L2/3 IT AI	2	0	7.149908	0	0.315793	0	0	0	2.2820482	0.7637805	7.588364	2.484207	7.640455	0	1.8372561	0	0	1.303537	0
162_L2/3 IT CTX	3	0	6.963458	0	4.466642	0	0.4781884	7.2117934	1.3367392	8.2132435	7.60413	3.207464	2.436631	10.207826	3.8565469	0	0	1.371336	0
163_L2/3 IT CTX	4	0	7.892495	0	1.490609	0	2.0641384	2.5507317	1.7452033	0.206038	8.09628	2.404369	7.82872	9.325374	2.2831166	0	0.52332	0.849865	0
164_L2/3 IT CTX	5	0	7.534272	0	2.150567	0	0.6304804	3.385669	1.2387104	7.5219045	7.355982	1.868016	1.254911	10.157107	3.6833363	0	0	1.447867	0
165_L2/3 IT CTX	6	0	7.122013	0	3.579709	0	0	3.596936	7.5893874	7.9931464	7.872811	6.96105	3.866623	9.89094	2.20226	0	1.255623	1.022618	0
166_L2/3 IT CTX	7	0	7.043956	0	1.926881	0	2.5363846	3.5989723	4.2976646	0	8.098937	7.050877	3.840572	7.515873	0.80278903	0	0	0.201054	0
167_L2/3 IT CTX	8	0	6.923822	0	3.831965	0	2.261766	6.581467	4.506941	1.960597	7.931811	7.215813	7.272733	8.125675	2.4629967	0	0	2.27229	0
168_L2/3 IT CTX	9	0	4.217309	0	1.632363	0	7.065357	2.6133683	7.2145195	0	7.886231	7.318973	8.922604	0	2.4064093	0	0.335286	1.99244	0
169_L2/3 IT CTX	10	0	6.954061	0	2.546267	0	0.85897285	3.264881	7.739544	2.591647	7.988943	7.585153	8.45838	4.1265454	2.4052677	0	1.156349	0.390729	0
170_L2/3 IT CTX	11	0	7.579526	0	1.642064	0	0.33474103	1.2506773	2.5819046	2.0089242	8.232485	7.561976	9.131737	2.1366534	2.906454	0	1.76131	0.18506	0
171_L2/3 IT CTX	12	0	3.327049	0	7.237247	0	2.09903	1.4408972	3.984618	0	4.612382	7.64573	3.155849	4.5913286	0.12696765	0	0	0.94323	0
178_L4 IT CTX	13	0	4.419474	0	0	0	1.6456127	0	3.2073545	0	8.18062	3.98855	9.994075	0.4653282	4.203379	0	0.07452	0	0
179_L4 IT CTX	14	0	7.615286	0	0	0	7.6175704	0.22345454	4.488948	0	8.142595	3.702263	9.800937	0.1468487	2.0345447	0	1.670297	0.232253	0
180_L4 IT CTX	15	0	7.613267	0	0	0	4.385025	0	8.093413	0	7.986114	4.492532	9.520249	0	0.30612278	0	0.603445	0	0
181_L4 IT CTX	16	0	8.028179	0	0	0	7.6152506	0	7.7187037	0	7.95012	4.084428	9.309097	0	2.2797053	0	0	0	0
261_L4 RSP-ACA	17	0	7.732956	0	0	0	0	0	9.001553	0	7.617445	6.309252	8.462214	0.7476566	0.7905417	0	2.619052	0	0
262_L4 RSP-ACA	18	0	7.27	0	0	0	0	0	8.49	0	7.27	5.71	6.69	0	0	0	0	0	0
182_L4/5 IT CTX	19	0	7.858007	0	0.536044	0	7.7733717	0	8.43345	0	8.340793	7.094622	8.862564	0.90481573	1.7972416	0	0.103899	0.62135	0
183_L4/5 IT CTX	20	0	7.943307	0	0	0	8.109217	0	8.486784	0	7.915432	7.014674	8.772932	1.2944162	1.7019888	0	0	0.123051	0
184_L4/5 IT CTX	21	0	8.158537	0	2.375887	0	4.342501	1.7577711	4.3624973	0	8.426224	3.422867	8.871109	4.304228	2.0983396	0	2.705952	0.906651	0
185_L4/5 IT CTX	22	0	8.425916	0	4.23644	0	4.1711874	0.14089645	8.079237	0	8.345735	3.433151	7.938903	8.185543	3.5998957	0	1.020758	0.998542	0
186_L4/5 IT CTX	23	0	7.977693	0	0.710653	0	4.3809123	0	7.4978333	0	8.150974	4.228524	7.99459	7.8417716	1.9989738	0	0	0.590189	0
187_L4/5 IT CTX	24	0	7.61738	0	1.781222	0	2.5260603	2.184205	7.829093	0	9.005766	7.539791	9.019403	0	3.6831346	0	3.03511	1.716063	0
188_L4/5 IT CTX	25	0	6.985044	0	0	0	2.6991398	0.44124356	8.27119	0	8.831026	8.087601	8.989151	0.022230601	3.6514986	0	0.845146	0.108782	0
189_L4/5 IT CTX	26	0	7.105583	0	0	0	0.505213	0	8.46971	0	8.343366	8.017249	8.756107	0	2.2711742	0	1.340847	0	0
190_L4/5 IT CTX	27	0	6.702747	0	0	0	6.928604	0	8.003744	0	8.651198	7.953365	8.281916	0	3.444247	0	0	0.549576	0
191_L4/5 IT CTX	28	0	7.543608	0	2.548212	0	3.1642044	0.52015704	8.040654	0	8.714277	7.807427	7.550328	2.6690998	7.2932796	0	0	1.335671	0
192_L4/5 IT CTX	29	0	7.62833	0	0.712147	0	1.8374243	0	7.689267	0	7.964809	6.886678	7.867075	0.9882243	7.28035	0	0	0.522949	0
193_L4/5 IT CTX	30	0	6.594236	0	0	0	1.9515558	0	7.1170564	0	8.500451	7.789693	7.722703	0	1.857726	0	0	0	0
194_L5 IT RSP-ACA	31	0	3.68183	0	0	0	0.94390196	0	7.738543	0	8.482464	7.835077	8.784893	0	3.0964348	0	2.315866	0	0
195_L5 IT RSP-ACA	32	0	2.559893	0	0	0	0.73894805	0	3.3490279	0	8.844821	8.350949	8.803954	0	8.683747	0	0	0	0
196_L5 IT CTX	33	0	6.864301	0	0	0	8.07607	0	7.5046635	0	8.528434	8.146843	8.516032	0	3.2724605	0	0	0.139033	0
197_L5 IT CTX	34	0	3.038717	0	0	0	7.433456	0	3.3948584	0	8.76434	8.465412	8.534143	0.059894424	2.691465	0	0.957865	0	0
198_L5 IT CTX	35	0	3.720441	0	0	0	2.0775418	0	2.007256	0	8.917421	8.142609	8.493457	0.08456311	4.237544	0	0	0	0
199_L5 IT CTX	36	0	3.747593	0	0	0	2.588477	0	1.8060441	0	8.922826	8.129479	8.858521	0.66275746	8.250132	0	1.494329	0	0
200_L5 IT CTX	37	0	6.459406	0	0.342488	0	6.5571647	0	2.5276017	0	8.954065	8.235142	8.144648	0	7.7948246	0	0	0.205142	0
201_L5 IT CTX	38	0	3.020357	0	0	0	3.1168644	0	2.0582113	0	8.895409	7.919488	8.843757	0	8.320587	0	3.838044	0	0
202_L5 IT CTX	39	0	6.387853	0	1.022684	0	0.501299	0	1.9253186	0	8.719511	7.79479	8.487124	1.4040686	6.8051925	0	0	0	0
239_L5 PT CTX	40	0	7.411168	0	1.719584	0	1.9560881	0	8.543607	0	2.661035	1.208439	0.981413	0	0.16396087	0	0	2.378694	0
240_L5 PT CTX	41	0	7.520879	0	7.261222	0	7.0845556	0	8.195	0	3.267778	1.014338	1.978296	0	1.5520741	0	0	3.086815	0
241_L5 PT CTX	42	0	7.654568	0	2.489167	0	8.6035	0	8.077439	0	3.175417	1.228	0	1.560909	0.71758336	0	0	3.757667	0
242_L5 PT CTX	43	0	7.822051	0	8.041606	0	1.0970383	0	7.5464396	0	6.702188	2.568223	2.842788	2.6128125	3.2154355	0	0	2.522388	0
243_L5 PT CTX	44	0	7.419259	0	2.70475	0	3.6585	0.94975	9.027408	0	1.29175	6.458889	3.232	0	0.90875	0	0.125	6.118889	0
244_L5 PT CTX	45	0	6.37504	0	2.054278	0	7.81792	6.1461906	8.700551	0	0.462567	6.676984	5.95624	1.3183422	2.170107	0	7.450953	7.0968	0.302727
245_L5 PT CTX	46	0	6.708412	0	6.737886	0	6.922171	3.882934	8.909722	0	0.171527	7.134374	6.258834	0	2.701535	0	7.317131	6.598295	0.100351
246_L5 PT CTX	47	0	6.599046	0	6.842298	0	3.5554452	3.3994007	7.573209	0	3.650146	4.039917	7.082997	0	8.467267	0	6.896605	6.513395	0
247_L5 PT CTX	48	0	5.950989	0	1.870549	0	7.119135	6.788791	7.8087025	0	3.381685	6.546722	7.491978	0	8.778962	0	7.574121	7.118956	0
248_L5 PT CTX	49	0	3.819286	0	0.677884	0	7.293785	6.9455557	8.03498	0	6.733572	7.451098	6.564269	1.59781	6.5675697	0	6.639326	6.952402	0
249_L5 PT CTX	50	0	6.163077	0	1.848	0	7.5046153	2.32	7.372308	0.749	6.376923	7.03	6.907692	7.482308	3.0525	0	6.084615	6.115385	0.2505
250_L5 PT CTX	51	0	6.828966	0	0	0	8.620689	2.8365116	7.42	1.0395349	7.626333	6.915173	6.85	8.034483	6.405862	0	7.109655	3.677675	0.203953
251_L5 PT CTX	52	0	6.441594	0	1.869029	0	7.902899	2.544272	0.5757282	0	6.593913	3.790192	0.911346	9.973286	0.9524272	0	0	1.116019	0
252_L5 PT CTX	53	0	6.921266	0	3.496897	0	7.5620155	3.2391264	4.2430105	0.16720906	6.865448	6.883051	2.23591	9.460708	3.7844799	0	1.402268	2.389907	0.43066
253_L5 PT CTX	54	0	7.197508	0	1.250913	0	7.2578144	3.9983168	3.1619558	0.17349964	3.796957	7.146627	1.11798	9.886854	4.0808268	0	0.283091	0.796797	0
254_L5 PT CTX	55	0	6.945656	0	3.465334	0	4.097854	6.382109	7.4608397	0.00939394	6.413871	7.761266	1.591211	9.159106	6.934805	0	2.684682	6.222899	0.328749
255_L5 PT CTX	56	0	7.595186	0	3.331816	0	2.9864986	3.919191	6.7524834	0.6072768	2.446815	7.443296	0.406638	9.622846	7.120574	0	0.070145	3.391852	0
256_L5 PT CTX	57	0	7.037895	0	0	0	7.866667	2.2541177	7.286842	0.63	3.295529	7.621754	0.827765	8.694036	3.0148234	0	7.33579	1.858118	0
257_L5 PT CTX	58	0	6.771856	0	4.180047	0	7.94348	3.563298	7.886649	0	3.6548	7.161467	1.328867	8.621259	4.1260304	0	7.227593	6.309329	0.096741
258_L5 PT CTX	59	0	4.260476	0	0	0	8.30708	0.58819	8.617542	0	7.752755	6.950678	0.077421	7.4326777	1.752647	0	2.978032	3.679501	0
259_L5 PT CTX	60	0	6.514609	0	0.516088	0	8.285652	2.7823071	8.102372	0	7.41608	7.22778	1.349617	8.397995	3.9329407	0	6.821455	3.819437	0.334359
260_L5 PT CTX	61	0	6.366605	0	0	0	7.381376	2.5214148	7.763946	0	7.444045	7.510454	6.717707	7.312819	3.201676	0	6.79633	2.045424	0
263_L5 PPP	62	0	2.156266	0	0	0	1.481849	7.710804	0	0	8.814151	0	0	0.93596846	7.5544276	0	8.668971	1.833074	0
203_L5/6 IT CTX	63	0	7.204389	0	0.594955	0	0.10087613	0.7476923	3.2379003	2.699304	9.219709	7.463977	9.255775	1.1141328	7.9879866	0	1.94855	0.676662	0
204_L5/6 IT CTX	64	0	6.916524	0	0	0	1.067066	0	1.9619044	1.6214979	8.574751	7.548713	9.4168	1.1422658	8.530423	0	2.417338	0	0
205_L5/6 IT CTX	65	0	7.016244	0	6.854672	0	0.88392556	0.21072759	1.0405076	7.836658	8.454506	7.483645	7.54944	6.925736	8.1576395	0	0	0.750322	0
206_L5/6 IT CTX	66	0	6.924928	0	3.33177	0	1.0871207	0.24926214	0.9017704	6.4505234	8.800116	7.546058	7.517813	0.79904854	8.639971	0	0	0.413735	0
207_L5/6 IT CTX	67	0	7.177137	0	1.443101	0	0	0	3.9898324	3.0906146	8.9785	8.112017	8.93774	4.319471	7.5664434	0	0	0	0
208_L5/6 IT CTX	68	0	7.016552	0	6.732674	0	2.6110888	0.6950923	0.30228585	8.24458	8.095821	7.344574	8.560032	2.9190083	7.895911	0	0	1.857353	0
209_L5/6 IT CTX	69	0	7.042321	0	1.048675	0	2.4993794	0	0.7075016	7.3230953	8.418742	7.442641	9.044538	2.8734999	8.651928	0	0	0.553742	0
210_L5/6 IT TPE-ENT	70	0	7.744822	0	0.04144	0	0	0	2.386627	0	8.801065	7.90375	2.72088	0	8.413018	0	0	1.07728	0

S5 File. Fig 6C, D. Cont.																			
	Gene	*CDH1*	*CDH2*	*CDH3*	*CDH4*	*CDH5*	*CDH6*	*CDH7*	*CDH8*	*CDH9*	*CDH10*	*CDH11*	*CDH12*	*CDH13*	*CDH18*	*CDH19*	*CDH20*	*CDH22*	*CDH24*
211_L5/6 IT TPE-ENT	71	0	6.689048	0	4.162903	0	0	1.4209677	7.013	1.3096774	6.6195	3.346129	0	6.971	3.379355	0	0	2.878387	0
212_L5/6 IT TPE-ENT	72	0	6.779151	0	0.227444	0	1.5455267	0	1.4048878	1.3643351	8.341731	6.844005	4.45846	7.9601808	3.264387	0	0	0.443804	0
213_L5/6 IT TPE-ENT	73	0	7.561147	0	0	0	0.276805	0	3.4114661	0.4372453	8.501884	6.965066	4.080227	4.547396	7.110395	0	0	0	0
214_L5/6 IT PFC	74	0	7.544808	0	0.508709	0	0	0	2.7611704	0.6787608	8.749128	7.502802	4.213115	7.275244	7.1555014	0	0	0	0
215_L5/6 IT TPE-ENT	75	0	7.253702	0	0	0	6.9317417	0	3.2615	0	8.443373	7.090149	7.526547	3.22338	6.7399106	0	0	0.11908	0
216_L5/6 IT TPE-ENT	76	0	7.150636	0	0	0	2.3962295	0	1.1736629	1.9911638	8.537078	6.800597	7.770929	8.1232605	6.873649	0	0	0	0
264_L5/6 NP CTX	77	0	4.157527	0	0	0	0.4963441	0	7.4029837	0	8.290726	8.53432	8.57129	0	11.30904	0	0	0	0
265_L5/6 NP CTX	78	0	4.764721	0	0	0	4.202766	0	8.32406	0	0	8.846471	0.463587	0	10.84178	0	0	0.149788	0
266_L5/6 NP CTX	79	0	7.367628	0	0	0	2.4294083	0	1.7571144	0	0.06229	8.842071	0	1.6045538	9.218197	0	0	0	0
267_L5/6 NP CTX	80	0	4.903921	0	0	0	3.1922965	0	7.7191534	0	0	8.471127	0.312716	0	10.133354	0	0	0	0
268_L5/6 NP CTX	81	0	7.643708	0	0	0	0	0	7.9243565	0	0	8.951614	8.172308	4.1680713	8.594813	0	0	0	0
269_L5/6 NP CTX	82	0	4.626333	0	0	0	0.1425714	0	4.761381	1.063619	1.515166	8.534	8.204719	0	10.189357	0	0	0	0
270_L5/6 NP CT CTX	83	0	7.675948	0	0	0	0.1960013	0	4.206987	0	0	8.540935	1.86279	0	10.837998	0	0	0	0
217_L6 IT CTX	84	0	7.608422	0	0	0	1.8716729	0.18278994	4.067374	1.9153157	8.700887	7.142714	8.351039	8.3934355	2.8678937	0	0	0	0
218_L6 IT CTX	85	0	7.19003	0	0	0	3.5707881	0.623833	6.751445	0	8.930775	7.591982	8.874262	1.7809708	7.475449	0	0	0.190307	0
219_L6 IT CTX	86	0	7.554478	0	0	0	0.5673466	0	4.1823716	1.6314163	9.082842	7.593769	8.432532	7.2205544	6.656453	0	0	0.511567	0
220_L6 IT CTX	87	0	6.979012	0	0	0	1.9662058	0	3.3786275	2.7513003	8.819473	6.816914	8.454992	8.510377	1.4843549	0	0	0.398873	0
221_L6 IT CTX	88	0	7.68155	0	0	0	0	0	3.6236188	7.095019	9.017805	7.200191	7.559751	9.915278	3.3628225	0	0	0	0
222_L6 IT CTX	89	0	7.714016	0	0	0	0.2146585	0	1.562234	7.5024457	8.40615	3.082339	7.80267	9.931439	1.5149777	0	0	0	0
223_L6 IT CTX	90	0	7.465696	0	0.176129	0	2.44308	0	1.9218041	7.4005113	8.486676	7.116962	7.969374	8.815564	2.6138628	0	0	0	0
224_L6 IT CTX	91	0	7.23968	0	2.803807	0	0	0	1.2109939	7.2262473	7.984795	7.415734	3.240092	8.656697	2.7842507	0	0	1.021768	0
225_L6 IT CTX	92	0	7.772726	0	4.179558	0	0	0	1.4793617	7.219173	7.746772	6.807193	1.469388	8.462094	1.444316	0	0	0	0
226_L6 IT CTX	93	0	7.375304	0	3.296112	0	0	0	1.6238414	8.771035	7.933659	4.696219	3.692499	9.6223955	2.9239676	0	0	0	0
227_L6 IT CTX	94	0	6.97831	0	0.479625	0	2.944995	0	0.9575629	7.6087575	8.341932	7.458504	8.461305	7.454129	4.646283	0	0	0	0
228_L6 IT CTX	95	0	7.029046	0	4.241627	0	1.3290102	0	1.6079772	8.609088	8.136025	7.313795	7.920029	8.087888	6.7734375	0	0	0.501037	0
229_L6 IT CTX	96	0	7.492586	0	4.296027	0	0	0.5113607	2.4237554	8.458864	8.048208	7.377349	8.279311	7.805419	7.300382	0	0	0	0
230_L6 IT CTX	97	0	4.27013	0	1.310813	0	1.1483464	0	0.9591615	8.29074	8.332555	7.475321	7.364499	8.789507	1.2980751	0	0	0.063966	0
231_L6 IT CTX	98	0	3.837697	0	2.839096	0	0	0	0.2448397	8.525305	4.875802	4.00718	3.515394	10.100043	0.51055396	0	0	1.117238	0
232_L6 IT CTX	99	0	7.170192	0	0.337006	0	0	0	0	8.189619	8.146698	0.887452	7.404528	10.343238	0.06566879	0	0	0.693734	0
275_NP PPP	100	0	8.016762	0	0	0	0	0	0	5.055208	4.505759	8.935509	0.072352	0.005072	10.593421	0	0	0	0
276_NP PPP	101	0	8.442114	0	0	0	0	0	0	3.765277	7.693834	9.033986	0	0	11.263586	0	0	0	0
277_NP PPP	102	0	8.964474	0	0	0	0	0	0	4.5077586	2.743793	8.72	0	8.183948	11.629474	0	0	0	0
278_L6 CT CTX	103	0	7.532255	0	0	0	0.7205163	0	2.851898	0.4216159	1.842362	8.623383	2.922252	0	10.06374	0	0	0	0
279_L6 CT CTX	104	0	7.467563	0	0	0	0	0	0.7112645	8.632952	1.003082	8.133892	3.091167	0	10.015799	0	0	0	0
280_L6 CT CTX	105	0	7.689242	0	0	0	0.2792473	0	3.480732	9.036286	7.565897	8.888463	0.227497	0	10.371382	0	0	0	0
281_L6 CT CTX	106	0	7.817832	0	0	0	7.2979913	0	1.1815133	8.045289	7.837753	8.847904	2.377389	8.9867115	10.491184	0	0	0	0
282_L6 CT CTX	107	0	8.520941	0	0	0	3.6865945	0	0	0	0.77412	8.484341	0.647188	4.5489397	9.221029	0	0	0	0
283_L6 CT CTX	108	0	8.571565	0	0	0	7.973436	0	0	0	1.983055	8.116411	3.061729	9.838277	9.850846	0	0	0	0
284_L6 CT CTX	109	0	8.047252	0	0.85651	0	1.6144923	0	0.2870625	3.3388522	0.554707	8.169055	8.392209	3.6058784	10.49269	0	0	0	0
285_L6 CT CTX	110	0	7.429265	0	0	0	7.332	0	0	0	0.287476	8.994203	0	7.823913	8.656177	0	0	0	0
286_L6 CT CTX	111	0	7.349959	0	0	0	1.890489	0	0.3573967	0.437741	0	8.68333	2.253677	1.0918639	9.570586	0	0	0	0
287_L6 CT CTX	112	0	7.647759	0	0	0	0	0	0.0174891	2.3090675	0	8.086615	8.030166	0	10.399532	0	0	0	0.658885
288_L6 CT CTX	113	0	7.961468	0	0	0	1.6552525	0	0.8201993	0.9601908	0	8.582371	3.355146	0.03952212	9.787535	0	0	0	0
289_L6 CT CTX	114	0	7.632734	0	0	0	4.139783	0	0	3.8559234	3.235474	8.313191	4.592768	7.3461804	10.66104	0	0	0	0
290_L6 CT CTX	115	0	7.997932	0	0	0	3.2303493	0	0.4893355	4.1085763	1.996077	8.673345	2.976561	3.3472133	9.702484	0	0	0	0
291_L6 CT CTX	116	0	7.344111	0	0	0	2.0930529	0	0.3158414	7.89232	4.63909	8.663997	4.544742	0.43171874	10.4611845	0	0	0	0
292_L6 CT CTX	117	0	7.337795	0	0	0	1.0880775	0	0.6706997	3.3492837	2.821178	8.4061	4.532161	0	10.530234	0	0	0	0
293_L6 CT CTX	118	0	7.616848	0	0	0	0.3762652	0	1.6692216	7.979071	2.565585	8.777843	3.541465	0	9.633066	0	0	0	0
303_L6b CTX	119	0	8.691015	0	0	0	7.8732576	0	0	0	7.302507	8.019872	3.919214	5.127656	10.578867	0	0	0	0
304_L6b CTX	120	0	8.470273	0	0	0	8.24506	0	0	0	7.778596	8.329969	2.008141	9.906226	10.60417	0	0	0	0
305_L6b CTX	121	0	8.647133	0	0	0	7.0630875	0	0	0	8.586974	8.552	2.728924	0	11.184094	0	0	0	2.268341
306_L6b CTX	122	0	8.154923	0	0	0	7.200968	0	0	0	8.91141	8.447547	0.579916	0.8511662	11.2154045	0	0	0	2.553855
307_L6b CTX	123	0	8.360756	0	0	0	2.7412806	0	0	0	9.007652	8.674087	0.227395	0.011362049	11.36113	0	0	0	3.986601
311_L6b CTX	124	0	8.390117	0	0	0	7.1344867	0	0.2172798	0	9.09156	8.799884	0.138395	8.420088	11.178396	0	0	0	4.019178
312_L6b CTX	125	0	7.825393	0	0	0	2.8915331	0	1.4326376	0	9.134062	8.619898	0	7.560825	11.212862	0	0	0	3.315527
313_L6b CTX	126	0	7.948462	0	0	0	4.4547987	0	0.4022701	0	8.892242	8.566738	3.315201	10.482415	11.249456	0	0	0	0.359598
314_L6b CTX	127	0	7.538117	0	0	0	3.0246303	0	0	0	9.005	8.620471	0.256265	10.070877	10.965059	0	0	0	1.044805
315_L6b CTX	128	0	7.7196	0	0	0	6.818943	0	0.4895229	0	8.977564	8.794314	0.272366	8.935828	11.501396	0	0	0	2.773924
316_L6b CTX	129	0	7.623736	0	0	0	4.0540323	0	0.9888224	0	9.040907	8.821142	0	9.032812	11.255331	0	0	0	3.518723
317_L6b CTX	130	0	8.327076	0	0	0	6.947396	0	0	0.8845899	7.859271	7.822532	8.001787	10.715	10.882709	0	0	0	0
124_L2 IT APr	131	0	3.656108	0	0	0	0.8522728	2.5981534	1.0830966	0	8.429237	0.689517	9.868432	8.130339	5.0084944	0	8.201822	0	0
125_L2 IT APr	132	0	3.191023	0	0	0	0.5249425	4.649885	1.645977	0	8.008966	0.29954	9.522759	1.084023	4.1705747	0	7.743621	1.68115	0
126_L2 IT APr	133	0	1.777239	0	0	0	0.8098639	2.4596033	4.0549736	0	8.095251	0	9.993798	5.021318	2.3286283	0	9.01237	0.006963	0
127_L2 IT APr	134	0	2.826652	0	0	0	2.9379203	8.331457	2.975708	2.994823	8.698934	0	10.2396	8.3874	2.1204867	0	9.114067	0	0
128_L2 IT APr	135	0	2.115549	0	0	0	1.645	7.983302	9.644637	0	8.808412	0	9.876361	4.9160714	0.46993685	0	9.120598	0	0
132_L2 IT RSPv-POST-PRE	136	0	1.034618	0	0	0	0	0	0.8839493	1.3168	3.811594	3.167309	9.185082	4.2709093	2.699309	0	4.231739	0	0
133_L2 IT RSPv-POST-PRE	137	0	1.981993	0	0	0	0	0	8.069878	0	2.955496	4.172114	8.382789	0	0.917755	0	4.735295	0	0
134_L2 IT RSP-ACA	138	0	4.539131	0	0	0	0	0.1444127	8.048264	0	7.598215	4.8309	9.200452	0	1.7619824	0	0.572124	0	0
146_L2 IT ENTm	139	0	8.274216	0	2.063423	0	0	0	7.4276767	3.5847652	7.581616	3.792384	4.319	8.3349495	6.6857576	0	2.125101	0.279733	0
147_L2 IT ENTm	140	0	7.798954	0	0.442364	0	0	0	3.8667533	8.217159	7.593682	3.005403	1.48761	10.140078	3.93413	0	3.9457	0	0
148_L2 IT ENTm	141	0	7.821964	0	1.140706	0	0	0	6.6173215	3.8075294	7.822679	3.619059	1.543294	10.133393	7.8148212	0	7.857719	0	0
149_L2 IT ENTm	142	0	7.715752	0	0	0	0	0	6.9499855	8.8522625	8.077109	0	3.941056	9.092105	1.476458	0	4.264467	0.873611	0
150_L2 IT ENTm	143	0	8.144211	0	0	0	0	0.7732143	7.311053	7.824737	7.68	0.967857	7.898947	7.1484213	3.1882143	0	6.916316	0	0
151_L2 IT ENTl	144	0	8.818308	0	2.910934	0	0	0	7.202677	0.4032246	8.76142	0.135353	8.754447	0	3.299837	0	0	0.515878	0
152_L2/3 IT ENTl	145	0	7.77449	0	0	0	0	2.2886207	8.638437	0.4389655	7.14433	6.9625	9.605938	1.9599305	7.174896	0	4.098472	0	0
153_L2/3 IT ENTl	146	0	8.506196	0	0.381218	0	0	1.0168679	8.485853	0.5620374	7.049451	3.580947	8.641171	3.3762114	6.5433607	0	6.594905	0	0
154_L2/3 IT ENTl	147	0	8.213082	0	2.841065	0	0	0.07034722	9.248374	0	6.740903	6.933897	7.837128	1.6443518	3.4172747	0	2.062171	0	0
155_L2/3 IT ENTl	148	0	7.26721	0	2.783231	0	0.1741539	1.9133846	3.2375386	2.148	2.532308	3.522121	8.990931	4.2058463	1.7127693	0	0	2.104923	0
156_L2/3 IT ENTl	149	0	8.193738	0	0.549723	0	0	0.119468085	3.270383	7.8234177	7.564377	1.290894	7.674537	8.941195	1.9245957	0	1.157489	0.436858	0
157_L2/3 IT ENTl	150	0	8.412774	0	0	0	0	0.95770735	4.3823414	2.1726341	7.422574	1.984806	9.242682	1.7467476	1.691256	0	1.325512	0.402049	0
160_L2/3 IT ENTl	151	0	7.501164	0	3.430954	0	0	0.9891451	3.5860438	3.3703785	4.138586	3.456044	3.874701	8.125401	2.2190437	0	0	2.394183	0

S5 File. Fig 6C, D. Cont.																			
	Gene	*CDH1*	*CDH2*	*CDH3*	*CDH4*	*CDH5*	*CDH6*	*CDH7*	*CDH8*	*CDH9*	*CDH10*	*CDH11*	*CDH12*	*CDH13*	*CDH18*	*CDH19*	*CDH20*	*CDH22*	*CDH24*
161_L2/3 IT ENTl	152	0	8.319309	0	1.731336	0	0	2.1647274	7.1751857	7.3067985	3.365197	3.177057	7.872161	9.515956	3.938416	0	2.485898	1.046185	0
172_L2/3 IT ProS	153	0	7.691606	0	0.501724	0	2.713793	0.039377164	8.656632	2.0921454	2.128552	7.652319	8.446823	8.039323	7.4753647	0	4.403711	0	0
129_L2/3 IT POST-PRE	154	0	3.704197	0	0	0	0	0	8.2799225	0	8.025231	7.83324	8.124164	0	1.2986765	0	8.431304	0	0
130_L2/3 IT POST-PRE	155	0	3.796435	0	0	0	1.517522	0	4.820982	0	1.603526	7.770726	8.0895	0	0.88016987	0	8.318064	0	0
131_L2/3 IT POST-PRE	156	0	3.733171	0	0	0	0	0	9.163434	0	8.085032	7.833024	7.996337	0	0.85130465	0	8.367821	0	0
141_L2/3 IT PAR	157	0	4.717216	0	0	0	1.6002353	0	3.9567842	0	2.842745	7.253918	9.212	0	1.6864706	0	7.648235	0	0
142_L2/3 IT PAR	158	0	7.180922	0	0	0	7.281475	0	7.7931714	0	7.022691	6.8366	9.429613	0	3.4257455	0	3.021665	0	0
143_L2/3 IT PAR	159	0	7.083387	0	0.553743	0	7.2846665	0	8.464213	0	4.495778	7.220824	8.987399	0	3.9201431	0	2.600877	0	0
144_L2/3 IT PAR	160	0	7.02757	0	0	0	7.0563617	0	7.415012	2.821872	3.900297	3.225639	9.523651	0	4.5140967	0	7.056385	0	0
145_L2/3 IT PAR	161	0	7.755442	0	0.536135	0	2.507598	0	8.595896	0	8.048725	7.209713	8.370801	0.057278797	2.3268363	0	7.96005	0	0
173_L2/3 IT ProS	162	0	7.753346	0	0.452187	0	0	0	8.921081	7.72	1.501555	8.042871	1.213608	9.555545	2.360945	0	1.641696	0	0
135_L3 IT ENTm	163	0	8.510398	0	0	0	0	0	8.483227	0	8.877886	4.281107	9.296451	0	2.0454044	0	8.553022	0	0
136_L3 IT ENTm	164	0	8.92262	0	0	0	0	0	4.186875	0	9.18639	3.053111	9.70679	0	0	0	9.006522	0	0
137_L3 IT ENTm	165	0	8.612889	0	0.434705	0	1.3031949	0	8.01473	0	8.998487	7.509232	9.501218	0	0.18425548	0	8.695529	0	0
138_L3 IT ENTm	166	0	7.957902	0	0.786584	0	0	0	8.966098	0	8.648081	7.081095	8.894599	0	2.1526744	0	8.111639	0	0
139_L3 IT ENTl	167	0	7.395017	0	0	0	0	0	9.329933	1.2546413	8.87204	7.173067	8.644682	0	4.021076	0	8.324209	0	0
140_L3 IT ENTl	168	0	7.897562	0	1.771296	0	0	0.6415873	9.115257	0	7.74229	6.616343	8.204264	0	6.706697	0	7.172459	0	0
174_IT HATA	169	0	7.409444	0	0	0	0	7.8188887	0	3.0767858	4.347857	3.934286	7.493333	9.230556	3.377143	0	0	0	0
175_IT HATA	170	0	7.442714	0	3.62781	0	0	1.8229524	6.838	4.133714	3.728571	4.095094	0	7.667	4.033019	0	0	1.876857	0
176_IT HATA	171	0	7.781624	0	2.598864	0	0	0.031136364	4.559034	7.880812	3.862557	3.75452	0	9.404488	1.9830682	0	1.033456	0	0
177_IT HATA	172	0	7.788841	0	2.126399	0	0.1019614	0.05045016	6.7571015	3.6599042	7.66686	3.908141	0.032412	8.738551	2.1798391	0	0.689132	0	0
233_L6 IT ENTl	173	0	8.095193	0	1.856232	0	0	0	0	4.6719594	7.922877	2.216562	0.318484	9.395233	0.22247232	0	1.609613	0	0
234_L6 IT ENTl	174	0	7.876747	0	2.349518	0	0	0	2.822249	7.0918674	8.096506	4.02548	0	8.635654	1.8283534	0	0.6112	0	0
235_L6 IT ENTl	175	0	7.360507	0	3.452203	0	0	0	2.1419492	4.7923727	8.432531	4.614	0	8.386666	0	0	0	0.670848	0
271_NP SUB	176	0	7.40875	0	0	0	0	0	2.8074648	0.4285916	0	9.357732	7.371895	1.8374126	2.636831	0	0	0.827394	0
272_NP SUB	177	0	3.395262	0	0	0	0	0	4.759622	0	0	9.331759	0.996531	1.658309	7.527249	0	0	1.337781	0
273_NP SUB	178	0	2.787965	0	0	0	0.6194109	0	4.846519	0	0.562115	9.087854	0.484838	0	8.525144	0	0	0.395643	0
274_NP SUB	179	0	2.571694	0	0	0	3.5621161	0	0.1755187	0	0.466058	8.66775	0	0	8.236544	0	0	0.457718	0
294_CT SUB	180	0	8.014072	0	0	0	0	0	0	2.1078212	0.536546	7.982549	0	0	8.577446	0	0	0	0
295_CT SUB	181	0	7.710449	0	0	0	1.607985	0	0	0	0.292015	7.896854	0	1.5583582	7.942809	0	0	0	0
296_CT SUB	182	0	8.284859	0	0	0	0.5256176	0	0	0.9850236	0.649175	8.101281	0	2.2616196	8.649858	0	0	0	0
297_CT SUB	183	0	7.890175	0	0	0	2.9728236	0	0	1.9017441	0.527294	8.484465	1.022941	2.0071764	9.011525	0	0	0	0
298_L6 CT ENT	184	0	8.625181	0	0	0	0	0	0	3.8242762	0	8.768107	0	0	9.113449	0	0	0	0
299_L6 CT ENT	185	0	8.69727	0	0	0	0	0	0	0	0	8.711038	0	0	10.542681	0	0	0	0
300_L6b ENT	186	0	8.666252	0	0	0	0.9929344	0	0	0	4.743097	8.456559	0.934933	0	10.9813595	0	0	0	0
301_L6b ENT	187	0	8.725064	0	0	0	1.4323504	0	2.9580343	0	8.876076	8.319554	0.288178	0	10.617707	0	0	0.02235	0
302_L6b ENT	188	0	8.786276	0	0	0	3.2808852	0	0	0	8.153716	8.10858	1.930584	1.7937298	10.570569	0	0	0	0
308_L6b RHP	189	0	8.440303	0	0	0	4.0397825	0	0	0	8.944839	8.622258	0	5.0478263	11.202258	0	0	0	0
309_L6b RHP	190	0	8.378788	0	0	0	0	0	0.645102	0.3702041	8.955152	8.055454	0	2.5071428	10.027059	0	0	0	0
310_L6b RHP	191	0	7.61027	0	0	0	0	1.9318519	0	0	9.060902	8.780714	0	9.621899	10.018524	0	0	0	0
318_SUB	192	0	6.227469	0	0	0	7.3469353	6.612073	8.909028	0	8.314817	2.794333	2.420763	0	2.1422615	0	6.963429	3.923297	1.042452
319_SUB	193	0	3.834522	0	0	0	7.735895	3.7201838	9.041315	0	8.332672	4.213718	0	0	2.8957353	0	3.928606	3.461792	0.079228
320_SUB	194	0	4.475781	0	0	0	7.5168514	7.305491	8.921571	1.3527349	9.077658	4.71702	0	0.3509916	7.689225	0	7.124318	1.444118	0
321_SUB	195	0	6.967863	0	2.422051	0	4.022536	0.39592594	7.7996154	0	1.489031	6.67346	0.383105	8.329445	1.7200285	0	1.630653	2.845157	0
322_ProS	196	0	7.057143	0	0	0	9.165417	0	1.63875	1.4044445	8.693542	7.31	7.967084	9.3354	0	0	1.57625	3.875556	0
323_ProS	197	0	7.893026	0	2.578771	0	1.8996918	0	0.8774403	0	1.763562	7.326	0.743733	7.7031794	3.6617808	0	3.42774	1.673151	0
324_ProS	198	0	7.227972	0	6.663611	0	3.6521962	0	3.6082244	0	0.28028	7.738951	0.225327	6.585944	1.2446977	0	0.276542	2.178692	0
325_ProS	199	0	8.238246	0	0	0	4.68069	0	0	0.282093	0.84686	7.397544	1.424768	9.123103	0	0	2.806628	0.624651	0
326_ProS	200	0	7.870135	0	0	0	9.49932	0	0	2.5782192	4.264747	6.876552	4.560645	9.852828	0.6354378	0	7.624207	2.119312	0
327_ProS	201	0	7.868318	0	3.888758	0	8.734167	0	0	0	0.855901	7.10486	1.609627	8.907757	0.70503104	0	6.670374	2.505342	0
328_ProS	202	0	8.422638	0	0.322573	0	8.601438	0	0	1.7847718	0.943843	7.046312	3.407967	9.745	0.48190084	0	6.959125	2.547469	0
329_CA1-ProS	203	0	6.959333	0	2.183441	0	2.701417	0.41364372	4.248259	0	4.329595	7.194303	7.872849	7.8	1.1852226	0	7.372317	2.334332	0
330_CA1-ProS	204	0	6.91354	0	6.754071	0	2.087219	0.91059	3.3005295	0	0.685828	7.490578	7.356432	4.1170206	2.583432	0	3.43432	1.583373	0
331_CA1-ProS	205	0	7.303448	0	0	0	0	0.6723846	0.5729231	0	7.048023	7.597674	8.732675	9.313932	1.0493846	0	8.820465	1.140692	0
332_CA1-ProS	206	0	7.560826	0	0.653833	0	3.039162	2.438	1.4677222	0	1.651222	8.008991	8.472774	8.813109	2.550838	0	7.867167	1.122961	0
333_CA1-ProS	207	0	7.611116	0	0.287197	0	0.6056395	1.099398	7.0140696	0.3801749	0	8.064502	7.896981	4.2295732	3.0706582	0	2.148873	0.02035	0
334_CA1-ve	208	0	7.68	0	0	0	0	0	3.5526357	7.5954022	0.660769	4.051308	3.256769	4.878837	0.043100774	0	0.710155	0	0
335_CA1-ve	209	0	7.452433	0	0	0	0	0	7.6720514	2.7845454	3.551637	7.332703	7.218108	0	1.0026315	0	0	0	0
336_CA1-ve	210	0	7.279532	0	0	0	0	0	7.6453123	2.7120833	8.371538	7.847344	3.419792	0	1.345625	0	0	0	0
337_CA1	211	0	7.160344	0	0.986086	0	0	0.3508388	7.5425863	0	2.414737	8.181891	4.567652	0	2.6058292	0	0	0	0
338_CA1	212	0	7.013666	0	0	0	0.1434791	0.29304183	7.67047	0.5222645	7.279033	8.022012	7.342094	0	1.7633365	0	0	0	0
339_CA1	213	0	7.411799	0	0	0	0	0	7.4110537	1.7830889	8.536099	8.168967	7.954182	0	1.1829503	0	0	0	0
340_CA1	214	0	7.439474	0	0	0	0	0	7.243841	2.314115	4.158584	7.910729	7.715099	0	0.28433627	0	1.705354	0	0
341_CA1	215	0	7.767931	0	0	0	0	0	7.175517	2.5811627	4.438605	8.01931	7.288	0	0.27930233	0	0.13907	0	0
342_CA1	216	0	7.319177	0	0.182885	0	0.1373352	0	4.370687	2.870824	0.722951	7.918461	1.236575	0	0.339863	0	0	0	0
343_CA1	217	0	7.223333	0	3.941579	0	0	0	6.7775	0	2.486316	8.838333	8.801666	0	1.2663158	0	0.285263	0	0
344_CA1	218	0	4.542639	0	1.764552	0	0	0	4.4956346	0.6746642	8.297207	7.645419	7.470829	0	1.6143657	0	0	0	0
345_CA1	219	0	6.93087	0	0.169429	0	0	0	7.129348	0.9104226	7.942766	8.273913	4.088572	0	1.3114285	0	0	0	0
346_CA1-do	220	0	4.46966	0	2.13945	0	1.8227996	0	7.4478254	0	8.180283	7.924455	3.611873	0	1.3408917	0	0	0	0
347_CA1-do	221	0	4.145099	0	0.288298	0	0.3357692	0	7.6094637	0	8.453136	8.085724	7.360612	0	1.2830024	0	0	0	0
348_CA1-do	222	0	6.897344	0	0	0	0.7078655	0	7.7765226	0.2928124	7.992693	7.938948	7.388355	0	1.3689506	0	0	0	0
349_Mossy	223	0	7.925	0	0	0	0	0	6.840667	4.4165215	9.19	2.603913	6.977334	9.071333	0	0	0	0	0
350_Mossy	224	0	8.064493	0	0	0	0	0	3.8948076	7.9252176	8.983334	4.402596	0	8.707971	0	0	0.106538	0	0
351_CA3-ve	225	0	7.868462	0	0	0	0	0	6.2991667	1.8473685	7.8575	7.894167	6.8675	0	0.5668421	0	0	0	1.201579
352_CA3-ve	226	0	7.947847	0	0	0	0	0	6.6874824	8.136389	7.54869	7.874552	6.86	0	0.36948597	0	0	0	0

S5 File. Fig 6C, D. Cont.																			
	Gene	***CDH1***	***CDH2***	***CDH3***	***CDH4***	***CDH5***	***CDH6***	***CDH7***	***CDH8***	***CDH9***	***CDH10***	***CDH11***	***CDH12***	***CDH13***	***CDH18***	***CDH19***	***CDH20***	***CDH22***	***CDH24***
353_CA3-ve	227	0	7.071429	0	1.090938	0	0	0	6.54	6.5538096	7.795238	4.17125	7.969524	0	0	0	0	0	0
354_CA3-ve	228	0	8.198268	0	0	0	0	1.4867924	7.680339	8.263239	4.111805	4.591203	8.036477	7.938268	1.666868	0	0	0	0
355_CA3-ve	229	0	7.753947	0	0	0	0	1.4604465	7.0717335	0	1.064375	7.906667	8.6024	3.5639286	1.5659821	0	0	0	0
356_CA3-do	230	0	7.989203	0	0	0	0	0	7.758633	8.219565	6.767592	8.196594	6.718202	0	0.6798058	0	0	0	2.879561
357_CA3-do	231	0	7.954964	0	0	0	0	0	7.6372857	8.5988655	6.998428	8.07036	6.732714	0	0.987109	0	0	0	2.386619
358_CA3-do	232	0	7.778958	0	0	0	0	0	7.5860415	8.30375	6.934375	7.936531	6.961021	0	1.3938888	0	0	0	1.616806
359_CA2-IG-FC	233	0	4.03798	0	0	0	0	1.1825253	3.899596	1.2738384	0.104848	7.836061	2.136768	7.8297014	1.4936364	0	0	0	0
360_CA2-IG-FC	234	0	4.125034	0	0	0	0	1.0106803	7.6413264	0	0.584014	8.03707	2.359396	0.5447651	2.4730613	0	0	0	0
361_DG	235	0	8.391736	0	0	0	0	0	3.2862356	9.582509	0.8694	0.027506	4.518453	0	0	0	0	0	0
362_DG	236	0	7.624593	0	0	0	0	0	8.361929	9.279188	1.7704	3.386357	8.091087	8.192298	0	0	0	0	0
363_DG	237	0	3.81373	0	0	0	0	0	8.973078	8.554446	0	2.727597	4.239628	0.9863198	0	0	0	0	0
364_DG	238	0	4.029735	0	0	0	0	0	7.994242	9.342416	0	1.358666	4.971254	4.2157507	0.9227168	0	0	0	0
1_CR	239	0	3.571394	0	9.435036	0	0	0	0	0	0	0	0	0.27346155	0.97392344	0	0	0	0
236_Car3	240	0	4.476397	0	0	0	1.4585222	0	1.5257677	1.8480059	7.158613	7.000691	7.048902	8.974598	1.554024	0	4.563403	0	0
237_Car3	241	0	7.159438	0	1.545492	0	1.7174797	0	8.107569	3.7413948	4.261629	7.348179	7.600358	7.9551697	3.1429605	0	2.609096	0.197182	0
238_Car3	242	0	7.047442	0	2.122978	0	1.3942956	0	7.8512306	4.0094457	7.20356	7.521643	2.088247	8.858331	2.0999022	0	0.391886	1.062033	0
19_Pax6	243	0	4.790437	0	1.897584	0	0	0	0	2.2134185	3.066585	4.107556	0	0.52356416	2.1797042	0	0	0	0.230633
20_Pax6	244	0	7.414342	0	0.793597	0	0	0	0	0	2.240351	7.412368	0	0.94894737	7.19	0	0	0	1.262544
21_Sncg	245	0	7.824483	0	0	0	0.3989449	0	4.275046	0	4.512981	1.875388	8.361862	0	0	0	0	0	0
22_Sncg	246	0	8.277297	0	0	0	0.3132432	0	0.8900901	0	3.882857	6.663649	7.655541	2.957027	0.14585586	0	0	0	0.196667
23_Sncg	247	0	8.421655	0	1.551923	0	0	0	7.2405753	0	2.394423	1.298702	7.7159	0	0	0	0	0	0.07899
24_Sncg	248	0	7.496832	0	2.144366	0	0.047027	0	2.5577848	1.9370124	2.087069	2.782091	8.810779	0	0	0	0	0	0
25_Sncg	249	0	7.872742	0	0	0	0	0	0.3528261	0	2.164891	6.891967	0	2.4425807	0.23663044	0	0	0	0.113913
31_Sncg	250	0	8.421865	0	0.840099	0	6.312564	0	3.3833334	1.8631095	6.432089	7.983801	0	6.170336	2.0055225	0	0	0	0
32_Sncg	251	0	8.385206	0	0	0	1.6098334	0	2.455083	0	2.735667	7.354794	0	1.3705	1.5104444	0	0	0	0
33_Sncg	252	0	7.802301	0	0	0	2.6839962	0	2.4787893	0	2.133731	6.652003	0	0.4844561	1.1459122	0	0	0	0
34_Sncg	253	0	8.130301	0	0	0	0	0	3.4561422	0	7.494504	4.244721	1.26702	0	1.2837564	0	0	0	0
35_Sncg	254	0	7.884554	0	0	0	8.349033	0	0	0.6819058	7.3891	7.338094	4.425605	2.638204	0.85013455	0	0	0	0
36_Sncg	255	0	7.586822	0	1.326839	0	7.577951	0	1.1191034	4.36325	7.926126	8.013015	7.519575	2.3404558	0.503819	0	0	0	0
37_Sncg	256	0	7.665031	0	3.118059	0	0.6310476	0	0.5593136	7.9698987	8.528846	7.884699	1.779953	2.4095197	0.99987984	0	0	0	0
38_Sncg	257	0	8.478079	0	3.058439	0	4.05127	0	2.0001323	7.2038736	7.477628	8.420437	0	7.9269567	2.7270713	0	0	0	0
39_Sncg	258	0	8.29926	0	1.480714	0	2.0338967	0	2.6394842	1.5649503	4.031505	8.16147	0	7.1000295	1.9592658	0	0	0	0
40_Vip	259	0	1.498566	0	0.992143	0	3.970717	0	3.0233207	0.4904528	1.809321	0.532491	8.37678	7.473977	0	0	2.782868	0	0
41_Vip	260	0	2.792945	0	3.163529	0	8.450501	0	3.417905	1.0221739	2.73073	0.535779	9.767676	5.066067	0	0	2.386634	0	0
42_Vip	261	0	1.706411	0	3.51678	0	8.368424	0	7.773006	0	3.974801	0.861093	9.548914	8.712321	0	0	5.050608	0	0
43_Vip	262	0	1.504655	0	4.473724	0	8.53936	0	4.410799	0	3.21795	0.363233	9.019712	9.287141	0	0	3.358856	0	0
44_Vip	263	0	2.679174	0	0	0	0.8107548	0	8.324856	0	7.835695	4.15875	3.055772	0.29921597	0	0	2.197394	0	0
45_Vip	264	0	2.486338	0	1.645168	0	0.5681732	0.10725894	7.7959146	0	8.302775	2.508714	8.233175	1.9408249	0	0	7.83921	0	0
46_Vip	265	0	2.7944	0	0.264679	0	3.030408	1.2120808	4.761735	0	8.574101	0	8.826984	0.59348446	1.02212	0	8.881975	0	0
47_Vip	266	0	3.649936	0	2.055258	0	0	0	2.7896774	0	8.481165	0	3.643974	5.1920643	0	0	4.446141	0	0
48_Vip	267	0	2.777531	0	5.104281	0	0.4670622	0.63081586	4.4741664	0	8.21372	1.792389	4.085407	8.548891	0	0	1.729042	0	0
49_Vip	268	0	3.196177	0	1.659226	0	0	3.2328002	0.8886375	0	8.094844	3.592804	1.694603	3.1018393	0.5410759	0	2.30404	0	0
50_Vip	269	0	3.873593	0	0.923345	0	1.0995333	0.079836	3.7452762	0	9.411308	3.619063	3.014134	2.8035367	0.6846954	0	2.810756	0	0
51_Vip	270	0	3.424348	0	1.740156	0	0	2.8813083	0.891539	0	8.512653	1.487824	4.622864	8.40895	1.6042595	0	2.284848	0	0
52_Vip	271	0	7.516305	0	0.896424	0	3.2257268	0	4.0071964	0	8.535502	1.566192	5.052122	0	0	0	2.193304	0	0
53_Vip	272	0	4.508489	0	2.841038	0	2.9814656	0	3.998849	0	8.714341	1.031295	2.960908	1.8487896	0	0	3.429755	0	0
57_Vip Igfbp6	273	0	3.668935	0	0	0	0	0	0	0	4.620049	3.53555	0	0	0	0	8.909524	0	0
58_Vip Igfbp6	274	0	3.2516	0	0.367886	0	0	0	0	0	8.128803	2.322057	0	0	0	0	8.348814	0	0
59_Vip Igfbp6	275	0	3.404135	0	0.895749	0	0	0	0	0	8.095799	0.549634	0	0	0	0	8.677254	0	0
60_Vip Igfbp6	276	0	2.83159	0	3.996095	0	0	0	0	0	8.435208	0	0.882348	0	0	0	8.958236	0	0
61_Vip Igfbp6	277	0	3.473525	0	2.208908	0	0	0	0.6910955	0	8.539719	0.51142	0	0	0.009499217	0	4.164319	0	0
62_Vip Igfbp6	278	0	3.338242	0	3.870797	0	0	0	0	0	8.416061	0.787865	0.317105	0	0.21826291	0	4.531155	0	0
63_Sst Chodl	279	0	1.307722	0	2.520127	0	0	0	7.8009434	0.7745569	7.432642	4.060127	0	8.439815	1.6193671	0	0.156329	0	0
64_Sst Chodl	280	0	0.132812	0	7.707464	0	0.1649481	0	7.307506	0	4.318309	2.300088	0	8.986753	8.3772545	0	0	0	0
65_Sst Chodl	281	0	0.036857	0	7.746774	0	1.603688	0	7.437957	0	3.794214	6.839785	0	8.827419	8.646596	0	0	0	0
66_Sst	282	0	2.347014	0	1.806309	0	0	0	3.8626256	9.409079	3.510046	4.144943	9.028152	10.678581	2.8663242	0	0	0	0
67_Sst	283	0	2.955355	0	2.783269	0	0	0	3.347472	10.375855	4.286137	3.646439	7.404121	10.458535	0.7169499	0	0	0	0
68_Sst	284	0	2.725712	0	4.135737	0	0	0	7.299507	10.034686	1.264011	4.263118	7.748396	10.180295	4.0721135	0	0	0	0
69_Sst	285	0	3.202666	0	3.182371	0	0	3.386685	7.7344155	10.220242	0.567707	7.065948	7.918267	9.823714	7.3432474	0	0	0	0
70_Sst	286	0	8.686105	0	2.013003	0	0	2.9855156	8.513928	4.2462425	7.721577	3.12694	1.07848	10.752966	2.0970588	0	0	0	0
71_Sst	287	0	9.20281	0	0.874846	0	0	2.8012226	2.990413	0	8.004314	0	0	10.157367	1.2432334	0	0	0	0
72_Sst	288	0	9.141789	0	1.517871	0	0	1.326983	0.924611	4.077738	7.634729	0	0	10.122788	0	0	0	0	0
73_Sst	289	0	9.557982	0	7.044435	0	0.4134186	0.9669535	8.059182	1.0602093	7.914069	3.020731	0	10.6788	2.0989523	0	0	0	0
74_Sst	290	0	8.618055	0	7.604445	0	0	0	8.064111	7.622752	8.133249	7.092662	0.992419	10.239769	0.7084769	0	0	0	0
75_Sst	291	0	9.149553	0	4.288405	0	0.043103	0	3.4807577	3.2623713	7.731663	0.86229	0	9.394615	0.6929133	0	0	0	0
76_Sst	292	0	2.03328	0	4.711383	0	0	0	0.3190087	9.420457	7.666283	0	4.259475	3.0783672	0	0	0	0	0
79_Sst	293	0	2.645676	0	3.717027	0	0	0	7.895306	9.312245	4.18446	0	0.907703	2.810946	0	0	0	0	0
80_Sst	294	0	2.148284	0	4.000714	0	0	0	7.5967035	2.2809606	1.701133	0.280985	2.968054	0	1.309656	0	0	0	0
81_Sst	295	0	2.953285	0	4.737601	0	0	0	4.280615	0.0546383	3.347678	0	9.654209	0	2.6022294	0	0	0	0
82_Sst	296	0	2.976809	0	4.285828	0	0	0.74354523	1.1289412	0	3.919098	1.424444	9.542842	2.3839958	3.5684674	0	0	0	0
83_Sst	297	0	2.880025	0	7.436037	0	0	1.0782045	1.0128678	0	2.789002	2.471396	9.150149	3.8783293	7.313172	0	0	0.138703	0
84_Sst	298	0	3.211725	0	7.529319	0	0	0.3757296	0.6462383	1.3196787	4.543891	1.462771	8.97304	1.0757965	7.705964	0	0	0.282758	0
85_Sst	299	0	3.839522	0	8.402556	0	0	0	2.0519466	1.0064627	8.202751	2.786233	4.576902	8.5100565	7.4458857	0	0	3.08708	0
86_Sst	300	0	3.412617	0	8.236082	0	0	0	4.090155	2.4823437	7.81422	2.630859	0.816719	9.274361	6.9799414	0	0	2.07938	0
87_Sst	301	0	4.564136	0	4.474737	0	0	0.21759398	7.3212223	4.068346	7.954045	1.784179	7.621477	2.0484962	7.4895453	0	0	0.30806	0
88_Sst	302	0	3.916235	0	7.979739	0	0	0	2.7149181	9.09711	7.862268	1.956217	0.332219	2.5272992	0.58992356	0	0	0	0
89_Sst	303	0	3.830295	0	4.454412	0	0	3.3879118	0	9.433392	7.802838	0.511357	0	8.926359	0	0	0	0	0
90_Sst	304	0	6.775222	0	3.07085	0	0	7.083256	0	8.976102	8.066583	0	0	9.827797	0.6202546	0	0	0	0
91_Sst	305	0	2.937445	0	3.961541	0	0	1.5074952	0.6841651	8.609871	7.895478	2.149942	0	3.3799043	1.9173155	0	0	0	0
92_Sst	306	0	4.462337	0	7.28459	0	0	2.8204372	0	9.2567215	8.219268	0	0	9.522	0	0	0	0	0

S5 File. Fig 6C, D. Cont.																			
	Gene	*CDH1*	*CDH2*	*CDH3*	*CDH4*	*CDH5*	*CDH6*	*CDH7*	*CDH8*	*CDH9*	*CDH10*	*CDH11*	*CDH12*	*CDH13*	*CDH18*	*CDH19*	*CDH20*	*CDH22*	*CDH24*
93_Sst	307	0	3.639303	0	3.472834	0	0	7.4075537	3.1819146	9.360903	3.481709	3.335889	0	7.9951067	0.9361306	0	0	0	0
94_Sst	308	0	6.534672	0	1.720436	0	0	8.5301695	0	9.567199	3.15296	1.19685	0	9.439294	1.1088068	0	0	0	0
95_Sst	309	0	1.765714	0	7.164	0	0	6.642	0	9.206	0.915714	1.712857	0.775714	8.546	0	0	0	0	0
96_Sst	310	0	6.772395	0	1.691631	0	0	8.978328	3.8102329	8.920456	1.927786	3.24747	0.823804	9.811559	2.588336	0	0	0.111479	0
97_Sst	311	0	3.973944	0	7.529422	0	0	7.637749	8.103772	9.108456	1.773289	7.003638	3.824104	8.820438	7.092974	0	0	1.605893	0
98_Sst	312	0	3.786513	0	7.891084	0	0	7.4179716	7.7373033	7.8341	7.698245	2.934365	0	9.482402	2.6163106	0	0	0.899511	0
99_Sst	313	0	1.784749	0	8.222479	0	0	3.273583	1.9340146	3.9262395	8.159765	2.139236	2.805837	8.3869915	3.657179	0	0	0	0
100_Sst	314																		
101_Sst	315	0	0	0	0	0	0	8.09	0	8.06	7.1	7.1	9.9	8.68	8.19	0	0	0	0
108_Pvalb	316	0	1.83354	0	1.044124	0	0	0	1.5515329	8.756973	3.494745	0	8.920055	3.8883212	0.12251825	0	0	0	0
109_Pvalb	317	0	0.647438	0	0	0	0	0	7.6665597	0	0.868187	0	9.403406	9.603734	0.49189863	0	2.888943	0	0
110_Pvalb	318	0	1.631255	0	0	0	0	0.59132844	8.1166115	0	1.096074	0	1.596593	9.655304	0.10439114	0	0	0	0
111_Pvalb	319	0	7.9029	0	0	0	0	0	7.118503	0.8075578	3.317013	0	1.276551	9.295714	2.9912987	0	0	0	0
112_Pvalb	320	0	1.474007	0	0	0	0.9112665	2.6885855	1.356292	0	0.590098	2.849105	8.995658	4.112937	8.113227	0	0.028561	0	0
113_Pvalb	321	0	1.466874	0	0	0	0	6.7602444	1.6892985	2.0459902	3.301097	2.088396	7.561614	1.4616013	7.0914145	0	1.476743	0	0
114_Pvalb	322	0	1.190544	0	1.924159	0	0	3.098612	1.3386252	2.6294332	6.874663	3.133694	4.995764	1.7578965	7.4180183	0	0	0	0
115_Pvalb	323	0	2.285151	0	0	0	0	7.3788605	0	0.4363309	3.11689	2.656057	0.773499	7.977717	6.9933076	0	4.254663	0	0
116_Pvalb	324	0	2.607797	0	0	0	0	1.2769053	0.5399694	2.266329	3.364749	3.905231	0.805958	8.303997	3.3174467	0	1.059221	0	0
117_Pvalb	325	0	3.124456	0	0	0	0	2.841361	2.2869089	6.794239	6.827024	1.17199	3.217539	8.019832	7.0750265	0	2.22167	0	0
118_Pvalb	326	0	2.15708	0	1.201443	0	0	0	0	1.0803103	2.64166	2.289256	7.831302	4.5497756	8.097909	0	0	0	0
119_Pvalb	327	0	1.856689	0	0.919185	0	0	0	1.4801673	7.64853	4.002207	1.163317	3.490179	7.5297494	3.433402	0	0	0	0
120_Pvalb	328	0	1.625302	0	0	0	0	3.7517061	1.126335	7.455748	2.995564	2.805013	8.132117	9.105764	3.5618062	0	0	0	0
121_Pvalb	329	0	2.385035	0	0.495849	0	0	1.679831	0	4.677448	2.498169	1.198806	3.988683	7.9541097	1.8916469	0	0	0	0
4_Meis2 HPF	330	0	7.636575	0	0	0	0	0	9.75452	0	0	8.098784	0	0.9540367	7.8858905	0	0	0	0
5_Lamp5 Lhx6	331	0	7.305263	0	0	0	0	0	0.0710917	0	0	1.135328	7.503618	2.4477293	0	0	3.308865	0	0
6_Lamp5 Lhx6	332	0	4.073582	0	0	0	0	0	1.4805102	0	1.212571	3.43522	7.415692	0	0	0	2.369776	0	0
7_Lamp5 Lhx6	333	0	7.275402	0	0	0	0	0	0	2.8865767	3.863791	2.736926	1.870702	0	0	0	7.4281	0	0
8_Lamp5 Lhx6	334	0	7.21007	0	0	0	0	0	0	0.8282791	1.672791	2.120326	1.387907	0	0	0	7.755664	0	0
9_Lamp5 Lhx6	335	0	3.771539	0	0	0	0	0	0	0	0	2.096154	2.654615	0	0	0	7.87375	0	0
54_Vip HPF	336	0	4.1485	0	0.939625	0	0	1.184125	8.515	0	8.678302	8.844339	0	0.14425	1.73725	0	3.92025	0	0
55_Vip HPF	337	0	4.456986	0	0.868904	0	0	0.25739726	4.3143835	0	8.872245	7.875102	0	2.9224324	3.469863	0	2.630685	0	0
56_Vip HPF	338	0	4.322667	0	2.433819	0	0	0	1.1231027	0	8.331815	4.854392	0	0	2.032328	0	2.211238	0	0
26_Ntng1 HPF	339	0	6.85353	0	0	0	0	0	8.027925	0	0	7.334314	0	0.9074026	1.4710389	0	0	0	0
27_Ntng1 HPF	340	0	4.39885	0	0	0	0	0	3.959375	3.622857	1.303451	4.675	0	0	4.332589	0	0	0	0
28_Ntng1 HPF	341	0	6.841299	0	0	0	0	0	7.680253	1.9246956	4.323131	8.157792	0	0.24617392	3.6760345	0	0	0	0
29_Ntng1 HPF	342	0	7.55128	0	0.893627	0	0	0	8.135897	0	1.948431	7.965498	0	2.9662132	2.02552	0	0	0	0
30_Ntng1 HPF	343	0	7.766154	0	1.43	0	0	0	1.456	0	8.32923	8.413846	0	0	1.16	0	0	1.076	0
77_Sst HPF	344	0	1.955	0	0	0	0	3.1660345	8.3205	0	2.513966	0	0	2.322069	0	0	0.752759	0	0
78_Sst HPF	345	0	2.967576	0	3.2398	0	0	0	8.503181	4.689495	4.2733	2.872828	0	9.59	1.629697	0	0.167273	0	0
103_Sst HPF	346	0	6.507778	0	2.1405	0	0.4095	0	7.0633335	2.2935	2.147	7.078518	0	8.610741	1.78175	0	0	0	0
104_Sst HPF	347	0	6.634	0	0.081029	0	0	0	7.2233334	4.753382	8.410444	7.381111	1.506912	3.6575	7.157778	0	0	0.505441	0
105_Sst HPF	348	0	7.557586	0	3.223529	0	0	1.0587059	4.3624706	8.544655	8.519123	3.193412	4.381177	4.214941	7.8957896	0	0	0.345529	0
106_Sst HPF	349	0	7.624404	0	0.832901	0	0	7.8648148	1.207963	7.714537	4.692099	2.074198	0	3.819136	1.0014198	0	0	0	0
107_Sst HPF	350	0	7.373717	0	2.617212	0	0	2.9497576	2.734491	8.739909	7.668559	2.664364	0.22303	0.03533333	2.7337575	0	0.228909	0	0
102_Sst HPF	351	0	6.971905	0	0	0	2.3159375	0	0.800625	7.94	4.152813	4.050313	7.822857	3.1940625	2.6271875	0	0	0	0
122_Pvalb Vipr2	352	0	3.442581	0	2.598226	0	3.252258	0	0	0	0	1.845397	9.163415	0	1.2516129	0	7.161463	0	0
123_Pvalb Vipr2	353	0	2.145181	0	0.63307	0	2.7105176	0	0	1.6799926	1.132175	0.567095	9.420012	0	2.4166346	0	0.195392	0	0
10_Lamp5	354	0	1.957989	0	0	0	0	2.1461306	0	0.1707372	7.647584	3.772741	0	2.464806	0	0	8.007272	0	0
11_Lamp5	355	0	2.231488	0	0	0	0	0.993841	0	2.310023	7.978152	7.696952	0	1.359404	0	0	8.317203	0	0
12_Lamp5	356	0	2.486385	0	0	0	0	0	0	0	3.204903	3.924682	0.598286	1.2661536	0	0	4.155433	0	0.137753
13_Lamp5	357	0	3.909244	0	7.631388	0	0	0	0	2.3628979	2.414147	8.043309	0	2.4482186	7.579146	0	0	0	0
14_Lamp5	358	0	7.238737	0	8.530018	0	0	0	0	4.270289	4.766007	8.072473	0	7.700973	9.320883	0	0	0	0
15_Lamp5	359	0	3.050697	0	4.761831	0	0	0	0	3.327201	7.389899	7.590587	0.033304	4.435402	9.424221	0	3.040216	0	0
16_Lamp5	360	0	2.392064	0	0.784908	0	0	0	0	2.0872421	8.025096	4.608937	0.162965	0	1.5089442	0	8.207344	0	0
17_Lamp5	361	0	3.899972	0	4.484246	0	0	0	0	1.2866201	7.878963	3.132989	0	2.8848882	4.8559217	0	3.473315	0	0
18_Lamp5	362	0	2.074935	0	0	0	0	0	0	3.2324722	4.568312	7.415534	0	4.163188	2.6917331	0	7.454827	0	0
2_Meis2	363	0	0	0	0	0	0	0	0	0	3.99	0	9.4475	0	9.0725	0	0	1.258333	0
3_Meis2	364	0	1.717778	0	0.822222	0	0	0	0	0	2.755556	0	0	4.632222	4.772222	0	8.403334	0.822222	0
365_Oligo	365	0	1.9737	0	0	0	1.9824215	0	0	0	5.710392	1.365941	0	5.0849304	1.0060294	0	3.847505	0	0
366_Oligo	366	0	2.94124	0	0.058843	0	2.8341322	0	0	0	5.123554	2.621157	0	8.823457	0.75206614	0	4.654262	0	0
367_Oligo	367	0	1.555391	0	0	0	1.0966406	0	0	0	4.84721	0	0	3.992248	0.058359373	0	4.707969	0	0
368_Oligo	368	0	1.071622	0	0	0	0.5108754	0	0	0	5.717162	0	0	3.1822636	0	0	9.0892	0	0
369_Oligo	369	0	1.722872	0	0	0	0.823391	0	0	0	3.610554	3.448793	0	2.5175173	0	0	9.649081	0	0
370_Oligo	370	0	4.196598	0	0	0	1.3610309	0	0	0	1.03732	9.088	0	0.066185564	0	0	9.186615	0	0
371_Oligo	371	0	7.551404	0	0	0	0	0	0	0	5.048721	8.943448	0	0	0	0	9.864561	0	0
372_Oligo	372	0	2.713548	0	0	0	0	0	0	0	8.926585	4.286613	0	0	0	7.763415	8.392927	0	0
373_Oligo	373	0	1.770271	0	0	0	0	0	0	0	4.81	1.18389	0	0	0	3.883383	2.153234	0	0
374_Oligo	374	0	0.3768	0	0	0	0	0	0	0	4.316324	0	0	0	0	2.522617	2.655557	0	0
375_Oligo	375	0	0	0	0	0	0	0	0	0	4.260854	0	0	0	0	2.298574	4.58116	0	0
376_Astro	376	0	3.785969	0	0	0	0	0	0	0	9.092862	0.12456	0	0	0	3.4392087	5.154804	0	0
377_Astro	377	0	0.623077	0	0	0	0	0	0	0	0	0	0	0	0	0	0	0	0
378_Astro	378	0	1.91205	0	0	0	0	0	0	0	1.835714	0	0	0	0	1.332963	2.254286	0	0
379_Endo	379	0	0	0	0	5.243694	0	0	0	0	0	0	0	0	0	0	0	0	0
380_SMC-Peri	380	0	0	0	0	0	2.253211	0	0	0	0	0	0	0	0	0	0	0	0
382_SMC-Peri	381	0	0.560849	0	0	0	0	0	0	0	0	0	0	0	0	0	0	0	0
384_VLMC	382	0	0	0	0	1.677755	0	0	0	0	0	0	0	0	0	0	0	0	0
385_VLMC	383	0	0	0	0	1.829231	0	0	0	0	0	0.553846	0	0	0	0	0	0	0
386_Micro-PVM	384	0	0	0	0	0	0	0	0	0	0	0	0	0	0	0	0	0	0
387_Micro-PVM	385	0	0	0	0	0	0	0	0	0	0	0	0	0	0	0	0	0	0
388_Micro-PVM	386	0	0	0	0	0	0	0	0	0	0	0	0	0	0	0	0	0	0
