# Supplementary material for: Classical cadherins evolutionary constraints in primates is associated with their expression in the central nervous system
Source: PLoS One. 2024 Nov 21;19(11):e0313428. doi: 10.1371/journal.pone.0313428 (PMC11581309; doi:10.1371/journal.pone.0313428)
Supplement: Attachment — Submitted filename: Bruses Responses to Reviewers.docx [file pone.0313428.s006.docx]

**Responses to the Reviewer's Comments**:

Line numbers mentioned below correspond to the final Unmarked version of the manuscript.

**Reviewer 1**

1*.Comment: "First, the abstract can be dramatically shortened."*

Response: As requested, the Abstract has been edited and shortened from 422 to 339 words.

2.*Comment: "Second (and minor), I would avoid definitive statements."*

Response: Adjectives suggesting the involvement of the molecule in a particular process rather than a direct participation have been used throughout the manuscript.

3. *Comment: "The final point is that I was surprised that the authors did not include genomic sequencing data from human, for example the ClinVar data available through gNomad."*

Response: Data from the gnmad database has been processed and added to the manuscript. In particular, the LOEUF scores of cadherins were correlated with the dN/dS ratios and with the level of CDH expression in 387 brain mouse cells. In both cases, the results agree with our previous results in that LOEUF scores positively correlate with dN/dS values, and negatively correlate with scRNA expression levels. The results of these analyses are presented in S4 Table, Fig 6 pane D, and S5 File. The raw data used for the plot is included in the S5 File. A description of these results starts in line 519.

Unfortunately, the number of clinical variants available in ClinVar varies substantially among CDH from over 500 in CDH1 to none in various type II CDH, making it difficult to conduct comparisons among CDS.

Finaly, in addition to the Supplementary Information presented in Tables S1 to S4 mentioned in the text, tables containing all raw data, data plotted in the figures, and results of the statistical analyses are compiled and included in the S5 File.

**Reviewer 2:**

*1.Comment: "On page 11, line 231, the authors discuss that “EC5 has been under higher negative selection in CDH2 and CDH4, possibly due to a functional role of this domain in these cadherins.” However, they do not provide supporting data for this statement regarding the function of this EC in those cadherins. The authors should elaborate on this issue according to the literature.*

Response: A paragraph addressing the possible role of EC5 in CDH2 function has been added to the Discussion starting in line 608.

*2.Comment: "On page 32, line 657, they discuss the association of CDH9 and CDH10 with ASD and, in line 660, how Cdh8 microdeletions and polymorphisms in humans are associated with the same syndrome. In both cases, they cite two papers (references 101 and 102). However, reference 101 relates only to CDH8, and 102 to CDH9 and CDH10."*

Response: The error has been corrected starting in line 656. Wang et al 2009 is used as a reference for the association CDH9 and CDH10 with ASD, and Pagnamenta et al 2011 for the association of CDH8 microdeletions with ASD.

3. *Comment: "Authors should clarify the election of the different statistical test for the different analyses."*

Response: A description of the selection of the statistical tests is described in the Methods section line 169.
